# Supplementary material for: Prevalence, associated factors and perspectives of HIV testing among men in Uganda
Source: PLoS One. 2020 Aug 7;15(8):e0237402. doi: 10.1371/journal.pone.0237402 (PMC7413494; doi:10.1371/journal.pone.0237402)
Supplement: S1 Table — (DOCX) [file pone.0237402.s001.docx]

| **S1 Table: HIV-related risky behaviors among male survey participants from selected villages in Mpigi district, August to September 2018** | |
| --- | --- |
| **Variable** | **Measure** |
| **Median number of sexual partners in last 6 months, (min, max)** | 2 (1,3) |
| **Median number of current sexual partners, (min, max)** | 2 (1,3) |
| **Circumcised (No),** n (%**)** | 621 (46.3) |
| **Sexual intercourse with non-partner in last 6 months (yes)**, n (%) | 326 (24.3) |
| **Consistent condom use,** n (%) |  |
| No | 848 (63.3) |
| Yes | 452 (33.7) |
| Sometimes | 40 (3.0) |
| **Condom use in last sexual encounter (No), n (%)** | 913 (68.1) |
| **Involvement in transactional** **sex (yes)**, n (%) | 68 (5.1) |
| **Drinking alcoholic beverages (yes)**, n (%) | 533 (39.8) |
| **Alcoholic beverage consumed*,** n (%) |  |
| Beer | 307 (57.6) |
| Spirit/ whisky | 10 (1.9) |
| Vodka/gin/waragi | 147 (27.6) |
| Local brew | 63 (11.8) |
| Other | 6 (1.1) |
| **Time of consuming alcoholic beverages***, n (%) |  |
| Early in the morning | 151 (28.3) |
| During my lunch break | 207 (38.8) |
| In the evening after work | 96 (18.0) |
| All day as I work | 74 (13.9) |
| Other | 5 (1.0) |
| **Weekly frequency of consuming alcoholic beverages***, n (%) |  |
| Once a week | 151 (28.3) |
| 2-4 times a week | 207 (38.8) |
| Every day of the week | 96 (18.0) |
| Once a month | 74 (13.9) |
| Other | 5 (1.0) |
| **Use of intoxicants (yes)**, n (%) | 63 (4.7) |
| **Type of intoxicants used^+^**, n (%) |  |
| Marijuana | 23 (36.5) |
| Kubba | 10 (15.9) |
| Khat | 3 (4.8) |
| Tobacco/cigarette | 27 (42.8) |
| **Desire for sex after using intoxicant or drinking alcohol (yes)**, n (%) | |
| No | 390 (71.9) |
| Yes | 142 (26.2) |
| Sometimes | 10 (1.9) |
| **Condom use in sex after using intoxicant or alcohol (No)**, n (%) |  |
| No | 435 (80.3) |
| Yes | 97 (17.9) |
| Sometimes | 10 (1.8) |
| *****Among those who drink alcohol (N=533), ^+^Among those who use intoxicants (N=63) | |
