# Supplementary material for: Prevalence, associated factors and perspectives of HIV testing among men in Uganda
Source: PLoS One. 2020 Aug 7;15(8):e0237402. doi: 10.1371/journal.pone.0237402 (PMC7413494; doi:10.1371/journal.pone.0237402)
Supplement: S1 File — (ZIP) [file pone.0237402.s002.zip › manuscript data/FGD Kayabwe 1 Eng.docx]

R: you can get the virus let us say like when you have used sharp instruments and other stuff.

M: Mr. XX!

R: Me what I know is that you can get the virus so easily most especially if you take alcohol because if you find someone that has it, and your drunk, and they call you uncle money, you don’t even remember about the condom, at this point they can kiss you so fast, when even the lips can be easily bruised from the waragi burns, so these kisses that we get, can be total sickness even when you have not directly engaged into sex.

M: Anyone else!

R: Me! You can get the virus for example when you get involved in an accident, say when you are helping your friends, most especially when they have a wound on their body. Even when you take alcohol, you always forget. You can even get the virus when you have not used a condom, or when you use a bad condom because it can burst, more so if it’s small, because it does not fit well.

R: I will also not defer so much from what these ones have said about alcohol, a person that takes alcohol stands very higher chances of contracting the HIV/AIDs virus because depending on the rate they use, the speed can be so high to the extent that they even forget to use the condom The other thing that is so common especially with the youths, I have specifically lost hope in youths, because they can tell a friend that I went to that woman and I enjoyed, and the friend also starts to feel that they should also go and enjoy. And that’s why there’s a very high rate of the spread of HIV in some parts of the country especially the lake areas. Now like me, I stay at Lukaya but from the conversations I listen to among youths are all about women, for example, I finished the other one, you can also go and try her out, that’s why I say that fighting HIV is still a very long journey.

M: I have a question specifically for men seated here, if someone goes and has sex with a woman, is it alcohol that forces them or it’s their thoughts?

R: Not at all, it is always the sexual appetite that is always very high.

R: It’s two sided, I can be alcohol, but there are some people who can’t control it, for example it can force someone to go and take marijuana, to go and do this, it forces him to do something that they had not wished to do at that particular time. Among people who take alcohol, you can only find two percent who are not talking about women. We do take alcohol but it always tastes sweeter when there is someone you refer to as mummy, so that even the others can all wish to be like you. Then after drinking and spending your money, you can never even think about using a condom.

M: Let us leave the issue of alcohol. Now, let us go to the topic of testing for HIV, what do you think about it?

R: My submission, I won’t divert so much away from the youths, they always bring programmes for testing and a person says I can’t even step there to put myself on pressure for nothing, and they completely refuse to go there. I can’t go there for people to start saying that you are like this, I will even start worrying. That’s what is in amongst the people, most especially the youths. So, for example if we had come here for testing, he can say that let me just go to the other place where they don’t know me and I test from there. Even I myself, my wife asked me to escort her to the health center, but she had never had about me testing before, so she asked me to escort her, and she even promised to pay the money herself, that was my first time to be tested, but I always felt like the seat I was in, was so big for me. I imagined all the women I had slept with and couldn’t think of any of them who was safe. When the doctor brought the result papers, he gave them to my wife but I always counted myself among the dead because I have had several sex partners right from when I was eighteen years and was not selective if one was HIV+ or not, but after testing when they told me I was safe, I had second thoughts and I felt it was high time I reduced the speed.

M: I think we are about to close up this topic. Fellow men, why do you think women actively participate in testing than men? This is the new topic that we have started.

R: For a woman, the most important thing is that they are too promiscuous, let’s say that if she got pregnant and then the man leaves her without giving her care, she will produce the baby and take care of it. But when she finds that her income is very small, she can start to move around men to get money so as to help her take care of her child. That’s why when a woman clocks 23 or 24 years, she can start to reflect upon her early years and the way she moved from one man to another she will then think about testing herself to see how she stands. She can begin to think that maybe she is sick or not. Then the other thing about women moving from one man to another is like this, even if a man gives her everything, she can still go and get around five men, well knowing that at every point she has a man there. Let’s say one for hair, women don’t have anything that they want, wherever she gets a deal is where she goes.

M: So do you want to mean that the men switch sex partners, even when the wife gives in herself to you every night?

R: For a man it’s different because a woman doesn’t appreciate anything, even if you give her everything that she needs, it can’t stop her from going to look for what is taking place the other side and the other side. When she has about three men, she looks for a way to handle her programmes so she very well knows that I will be meeting this one at this hour.

R: For me about this topic, it is the problems of women being housewives, especially in town. At home, especially for me a low income earner, if you leave 10,000= UGX, a woman can look nice and when she looks nice, it can’t stop my friend from going to try her out, he can also give her 10,000= UGX, so now if she starts to do such things, they go on pulling her around up to a time when she doesn’t consider you as important. Then from there, she can start to think about testing herself to see how she is standing.

R: For me about the topic for women, men just have to cool down because they have to look for money.

M: We have not ignored the issue of working, but why do women engage themselves more in testing for HIV compared to men?

R: Women nowadays can be calculative and be like now if I go to the other one, he can give me money. And because he can offer her help but he can’t move the machine very well (meaning is not good at sex), she will have to look for one that can move the machine very well (give her good sex). So she will leave this place to go and look for one that can move the machine better, but then she finds that even the one she has got can’t move it well, she keeps moving from one to another, up to a point she feels like she has over moved so she chooses to go and test how she is standing, and if she finds out like when she is already HIV+, she chooses to also infect some others.

M: Please you can talk!

R: I will not differ so much from what my friend has said, poverty and the big desire for free money is the problem and its source.

R: For me what I am saying, women are so fearful compared to us men, a man can realize they are sick and they keep quiet, but for a woman she will say, let me go and test if I find myself positive, then I start treatment.

M: We have closed that topic, what are the benefits of men testing for HIV/AIDS?

R: The good thing about men testing is that, you can move to a certain place and you think about testing, if God helps you and you find yourself safe, then you can reduce the engagement in casual sex. Actually most just abandon everything, now like me who has a wife, if I find out that I am negative and my wife is also negative, I can just settle down because I really fear the HIV virus.

M: Then this is saying that for him he differs from you because for him when he found out he was negative, he just reduced on the speed.

R: Me what am saying is that, it would be important to test and if you find that you don’t have the virus, you just have to abstain from there and then.

R: For me, I am encouraging all men to go and test because it is good. You can even take good care of the children you have produced.

M: Mr. XX has something to say!

R: For me, testing is very good, it is necessary for a man to test, but we work so hard. At times you can even get to know that you were tested and was found HIV+, but then when you don’t have the time to go and line up for treatment. So in most cases I think it is good for men to go and test but we don’t always have time to go and test, we always have a lot of work to do, everything is demanding for your time, and by the time it gets to the evening time, it is when you find a widow. The truth is that we really fail to get time to go and test.

R: Me I used to say that I can’t go to test, but one time with my wife we chose to go and test, but it is the woman that forced me to go and test, because me I already had a feeling that I was already sick (HIV+), but then I got to find out that me I was safe yet it’s her that was sick, so from then, I just give her money to take care of the children, and I chose to forego having sex with her. Right now, I just make sure she has taken her medicine properly.

M: We were still on the topic of testing for HIV/AIDS.

R: Me am saying that testing is good and it would be important for everyone to go and test. Only that when people find out they are sick and they instead just choose to spread the disease, but it would be important to test.

R: And the other thing about women is that for them they change blood which men don’t do.

M: I think we should conclude that topic, what would inspire a man to go and test himself?

R: What would inspire us to go and test, now like us who have produced children, and they begin asking for books, you can get to a point and think, I should test for HIV/AIDS, if I find out that am safe, I can go and raise my children.

R: Me am saying, it would be better if a man goes with his wife, let’s say for example if you have gone to test, to find out if any of you has it or not.

M: What would inspire you to get tested?

R: What would inspire me to go for testing is the reason this man has talked about; you could find that my wife has it when I don’t have it. Now such a reason would motivate me to go and test. Only that is what is most important to us the youths but we don’t have time to talk about what HIV is . If you could be with your friend there and they give you such a thought, but we the men don’t have such time to say let me sacrifice this time and I go for testing. But women have so much of that time, because it’s rare to find a group of over five men when they are talking about HIV.

R: But when the other man told us, if your madam tells you that you check this is my result slip, I went and tested, you still can’t trust so that can be a basis for you to go and test, because by the time you seduced her and brought her home it’s because you trusted her.

M: But still you can’t follow her results to know your status!

R: True, that can’t happen, because all men like we are here, no one has a wife at home when they can say that this one is mine alone, because they also have some men out.

R: Now for me what I think would inspire us men to go and test and even the youths, now like me am a man, I have a child and I even have a wife, but it would be easy when you the organizers for such events for example say that there is an event about teaching people, where we can learn about testing for HIV and then you offer something that is entertaining, so when they reach there and they see that you are doing your stuff, they won’t be afraid to get tested, because the youths love music so much and if we see entertainment like music, we could just branch off and join. For me I think that’s what would work.

M: Is there anyone wishing to add on that or we can conclude that topic?

R: I won’t also differ from him; we need morale boosters which is supposed to come from you people to us

M: Is there anyone to conclude?

R: Let me just add on. What I want to say is that testing is very good, but when we are going to test, they would have separated us, and they say that men should line on this and the women the other side, so that we do it the way it’s done in the mosque, so that we don’t go back home and the woman begins to suspect my mood.

M: But now you are saying that men should be on the other side and the women on the other side, do you also behave like that when you are at home, I thought that you were supposed to collaborate and work together?

R: But like the other one said that we men are rigid, but if a woman finds out that you are sick, they immediately pack their stuff and go, a woman is not someone that you can trust, once they find out that the husband is sick, they just pack and go.

R: But even me when I realize that she is sick, I can’t look back, I just run away for my life, I can’t wait for anything else.

R: That depends on how many children you have with that woman, now for example you have like six children with her, do you still run away?

M: now, there is someone that has asked me that if it was me, what I would do. We all understand that there are families where a man has HIV but when the woman doesn’t have and where a woman has and the man doesn’t have, and they give birth to children and the man stays alive and the woman stays with her virus, and they don’t spread the disease to each other, so now if you run away, who will take care of the children?

R: There at least I would rather keep sending support.

R: Me I think for all the time you have spent with the woman when she is not spreading the disease to you, it means that there’s something that is bringing you together meaning that even when you go and get another person it might be where you could even get the virus from, because from all the time you have spent together you may even develop a thought that maybe it could be there when it has just not yet manifested. For sure, you produce six children with the woman and you haven’t contracted it, where else do you really think it will come from?

M: We have finished that topic, so what challenges do men find when they go to test for HIV, those that stop you from going to test?

R: The issue of jobs, and even getting worried about being sick or safe, and you choose to keep in that state when you are not sure.

R: Me I can’t get worried because I don’t move around women.

M: When did you last test yourself?

R: Me! Only that this year I have not tested yet.

M: So what happened to you that you have not yet tested for the whole of this year?

R: This year!

M: Yes, first give me a reason, now like yesterday the health workers came, why didn’t you test yourself?

R: I am always travelling I was not around, and sometimes I am in the garden digging.

M: The man behind, please give us the challenges that you face.

R: Challenge!

M: Or what stops you from going to test!

R: What stops me most from going to test is that I do a lot of heavy work, now if I find out that I contracted HIV, my heart can be so sick, I work a lot and now if I start to worry plus taking medicine!

R: And something else that makes people worry, is that they don’t have money, now if they find out that they are sick (meaning HIV+), they will always be worried. But if they see how they take good care of the patients and they are in a place like Kayabwe, if there is a clinic for HIV+ care, when they take good care of them, they get rid of all the doubt that they had, and become confident about the medicine, so they can also choose to go because they can see the benefits that the others are getting from there.

R: If the HIV clinics were near, someone can go there and get medicine. But then someone first has to go to Buwama (meaning far from where they have tested) to get medicine, sometimes Lukaya, yet if the private clinics had that medicine, like I can leave this place and go to Mugisha’s and I get treatment, but then you have to suffer looking for the medicine.

R: You know why some people don’t go for testing, they have a challenge with taking medicine, they even threaten you by telling you that the medicine is expensive, and hard to take, but what I would suggest is that there should be an innovation to make HIV treatment injectable. For example, when women are raped, why doesn’t the government put that medicine at the clinics so that when I know that am going to the other lady I go with it (meaning Post Exposure Prophylaxis).

M: Listen to this man, so you now want to promote raping of women?

R: No but you see, there is a medicine given to those that have been raped and it works 24 hours, why don’t they put that medicine in clinics so that we can get it.

R: I also don’t agree with that because it is like the government is encouraging us to go and spoil ourselves because the medicine is there.

R: It’s still the same.

R: No please, that’s like going to step on people’s rights.

R: You have not understood what I was trying to tell you, because if I have my partner that is positive, I can go and get the medicine after the action, I take it.

M: This man is talking about PEP, do you know PEP?

R: I know it. but the challenges that are in PEP are uncountable, to start that medicine is not also an easy task.

M: You are saying that it’s not easy but then why does it become easy when I have had sex with someone that has the virus?

R: It is only when a problem has occurred that you can take, but it’s not something you just think about and you then go and get the medicine, that is very strong medicine.

M: How do the rest of you think about it? Why don’t some people want to go and test, this old man had not yet talked at all!

R: I had also once been stopped by that factor, because in my heart I thought, if I go and test today, because me I had my children, I think my first born was seven years old, and the other was four years and the last born was three years old, so as I thought about it I wondered if I go and test now, I may even die before my children grow up, I think it took me close to five years when I had not seen any woman.

M: What can be done to increase on the number of men that go for testing? If that is done, you will ask questions about whatever topic we have looked at.

R: First and foremost some men don’t have money, if they hear that there is testing but they also offer a token of 5,000= UGX that can help to increase the number of men turning up for testing, they will even come make lines, when whoever they test is the one they give money. It can then be easy to test because sometimes, one could have come to test but when there’s someone that is going to give them a job, but in case they are going to get some money after testing, they can come and test and get that money and later go and do other activities.

M: We have understood that, don’t you have any other opinion?

R: What that man has said is true, because if you tell that the other side they are testing but when they give you money, when I even come and get the results instantly, when I come and find that the results are bad, I just branch off to a bar and drink alcohol.

M: But you had already given out all the answers for example that man said that, the number of trainings should be increased, and bringing the disco, even putting a private place, you had already forgotten about all those answers, but then you added the issue of money.

R: With that, even someone from deep village can be inspired to come and test.

R: Even when you have not left any money at home, you can say that let me go and get this money and then I will cover up after getting it.

M: Do you have any other question or we can proceed?

R: My question is directed towards our leaders, they come and promise to help us, after voting for them, they just stay in the parliament increasing their salaries, they don’t even care about us at all.

M:: I don’t have an answer to that question, but you allow me to give you direction on how to use the other method I told you about, we pass through it, only that it appears like time is against us. This method will be using saliva to test for HIV to see if you have it or not in addition to the method that uses blood, is there any other method that anyone of you knows.

R:: Me I know one for blood.

M:: Is there anyone that knows any other method apart from this one?

R:: This one is for saliva! The truth is that there is no other method that we have ever heard about apart from the one that uses blood.

R: For us the old ones, we could always use the eye, we used to check by seeing, because the HIV virus fears the eyes so much.

M:: You want to mean that you were using your own eyes?

R:: Yes that is true, and it helped us a lot and that’s why we are still alive up to now.

M:: Let us now talk about this method. This method is used when you are at home, you can find time and you test yourself, you can even test yourself when you are at the work place, even if you work in a quarry, this method was brought to increase on the number of men that test for HIV/AIDs after it was realized that the number of men that turn up for HIV testing is very low compared to that of women. It’s like a sealed tea packet, like you see from number one, in the box for number one there are two things, there’s a bottle, I think you can see it in number two.

R:: Yes we can see it.

M:: Even in number five there is something that looks like a toothbrush, do you see it? Now that which appears like a toothbrush that is at number five is used to get the saliva, you pass it on the upper and lower gums, the bottle has solution in it that tests the saliva to know if someone is positive or negative, at number three, you open it, at number four you raise it the rack, at number five you remove the pipe-like thing, it’s the one you pass around the gum like you see at number six. After passing it through the upper and lower gum, you put it in the bottle that you opened at number four, are still together? At number seven, you raise it, at number eight you leave it standing as you are watching, after twenty minutes you can get the results.

R: Do the twenty minutes show me if I am sick or alive, what I want to know is that how do I get to know that am either sick or alive?

M: I have some other four questions this other side, let me get them altogether.

R: How do I get to know that am either sick or safe?

M: Do you see this thing that you held that looks like a toothbrush, on top it’s big and at the bottom it’s small, and it has some dots, you can turn the paper After turning the paper, the dots are enlarged, on those other papers, they didn’t put it, but when you look at this one’s paper, I wrote them using a pen. If it brings two lines, when you look through the bottle, if it brings two lines, then you have the virus, and if it’s only one line, then you don’t have the virus, but then if it doesn’t show anything, then you have not used this method correctly, for example let us assume that you were just from eating, or when you get saliva that has food I it, or when you pour water in the bottle, you dilute the solution, or when you use saliva from the tongue, the tongue comprises of very many things.

R: So how many minutes should pass after you have brushed?

M: After brushing, it requires that you spend one hour, from there you can get good saliva to use, and this thing that you use, does not cause any damage to the gums.

R: Let us say that I have used this method and it has showed me two lines, meaning that I am sick, where do I go to get medicine?

M: When you wake up feeling weak, is there anyone that sends you to go to the health center?

R: It’s just that the health centers I go are these very near ones.

M: When you go to the health center and they tell you that you are sick when you didn’t expect it, do you move on with that woman or not?

R: the woman I get is the reason I actually don’t go for testing, because we have already said that there is always no time to go and but if I go and we test when she is negative, there’s no where I can go on with her.

M: Anyway you are supposed to go to the health center and take medicine.

R: But then for those clinics, if whenever you go for testing, they tell you to go to the health centers now like Buziga, and you go with the results slip does it speak for you or you again have test again.

M: This method was brought to make such methods simpler.

R: So now if I test and I find myself sick, am I supposed to go to the health center and test again.

M: Only if you don’t feel contented with the results.

R: So now this method is just to encourage me to spread the disease further.

M: So you want to mean that if someone gets to know that they are sick from the health center, can’t they go and spread it?

R: Now let me also ask, who are you?

M: We already talked about that long ago, only that maybe you came in late, but never the less, we came from the ministry of health at the district and we moved with some officials from Mpigi district, anyway, what do you think about this method?

R: This method is good and it has no problem at all, because why it is good is that it brings all of us together even those that fear to test, you just buy it and you go to your room.

This method would have been good if there was a clinic for it.

M: So you want to mean that those that test blood have their own clinic?

R: It’s just because am wondering if I will be buying it, or it will be for free, and like am in a quarry so after finding myself with it, where do I go?

M: You just go to the health center, you are over making it appear difficult, so if you get a stomach upset, where do you go?

R: When my stomach is hurting?

M: Don’t you go to a health center?

R: Like you also know, AIDS doesn’t hurt so people just take it for granted, because it takes a lot of time to hurt.

M: How about the disease attacking you?, let me try to explain it to you.

R: The problem you want to explain is like this, what stops from going to those health centers is, the patient is there and they are on treatment.

M: Let me explain to you

R: You won’t explain to me and I understand.

M: Let us just go ahead, let me ask a question going to all the men that are here, can someone go to the health center just to check how they are standing when they have not gone with their wife, just to know about how they are standing. If they tell you that you are sick, what do you do next? Do you think the health workers give you ARVs and if they are not there, do you think they tell you to go further?

R: The further it affects you, the further you move from one clinic to another in a search for medicine, you may get fever and you choose to go to Nkozi, reaching at Nkozi when it is just increasing and from there you just choose to proceed to Mulago.

M: Let us go on, what brought us here is the fact that this method has not yet started working because of the research that we are still doing up to now, we can even test, so what brought us is that we want you to tell us how good and bad the method is.

R: There isn’t anything bad with using it only that the only bad thing with it is using it badly, the thinking is not the same but when you test yourself, you can be the only person to know your results, in a house where there are pangas, you can cut someone especially when you are confident you had never moved around, someone can easily cut the other because people have different so one can even choose to kill themselves, or even to spread the disease further to other innocent people.

M: I told you that what brought us here is doing research, not so? we shall come back and that will be to bring about samples that people can use to test themselves, do you understand it? But when they start working amongst people, they will be put at government health centers and pharmacies. In the pharmacies, they will be for sale, and in the government hospitals, they will be free of charge.

R: they will all be for sale because there isn’t any morefree things!

M: You don’t need to argue, they are there.

R: Here even the ARVs are just bought.

Not all of them

M: Don’t lie to people; medicine for HIV is not for sale in all government hospitals.

R: They are free; they get them free of charge.

M: Is there any other opinion about this method?

R: For me I just have a question about this method, if I find out that I am sick, what do I do to get the medicine, for example at the Hospital at Nkozi when I have tested myself positive?

M: This thing that gives you the results, even if you keep it for forty days, it still keeps your results. This thing can only be used on one person and only once, once you are done using it, you can either burn it or throw it away, that’s why you see that they put a dustbin here, you either burn it or throw it away but it still keeps your results though it’s always of no use provided you have understood the results, you may not need to go with it to the health center, you can’t go and lie to any health worker about your life.

R: So now like I have tested myself and I understand the results very well and I throw away the results, what if I go to the hospital at Nkozi and they refuse to believe that I tested myself, when they want to prove for themselves that am not faking.

M: By the time we shall start using that method, even the people at Nkozi will know about this method, and even in the health centers, it will be there, it won’t be about one person only, no! my question is like this, give me answers and we then wind up, my question is Mr. YY, I don’t want you to run away, this method uses saliva, but can the HIV virus be spread through saliva?

R: Please come again!

M: This method bases on saliva to test if someone is HIV positive or not, so I want to know, does saliva have HIV?

R: I also wanted to ask you because all along I knew that to test for HIV, they use blood, so I was wondering about it was discovered that saliva also contains HIV?

M: I asked if you had a question and no one bothered to ask me.

R: Me I know that it can even pass through the women’s breasts.

R: A child is breastfed for six months; wouldn’t he/she have died there and then?

M: Let me explain to you about what this method bases on, I want to give you an example from which you can relate, if this man has a machine here and he removes it, when you come, what do you see to realize there was a machine?

R: You can find the marks of the tyres!

M: So even this method is also like that, take the HIV virus to be a system in your car, then it has symptoms that arise due to it, so we can take that to be the marks, it’s also like going to a home, where they graze goats, how do you tell there are male goats when you have not found them there?

R: You can still feel the smell.

M: That is still the same with the signals that are in saliva in the mouth, like the smell of the he goat can be, it’s the same way the HIV virus can be in your system. This method will base on the signals that are in saliva, but the saliva has no virus and a person can’t contract the virus through saliva unless they kiss a person with wounds in the mouth and the blood joins together, we explained all that at the start, and I think we all know about how someone can contract the virus. Thank you so much for your time gentlemen, unless there is any other question.

R: there isn’t!

M: Am going to read about six people who will stay behind there are some papers we want you to help us sign, we have forms that show that you have helped us in this research, on top is where you put the name, and here in the middle is where you put your signature and at the bottom is where you put the date. Is there anyone with a second pen?

R: Is there money for this?

M: What do you want old man, these are my papers

R: I want papers that am going to sign on

M: Write your name here.

R: What is the date today?

M: Today is twelveth.
