# Supplementary material for: Prevalence, associated factors and perspectives of HIV testing among men in Uganda
Source: PLoS One. 2020 Aug 7;15(8):e0237402. doi: 10.1371/journal.pone.0237402 (PMC7413494; doi:10.1371/journal.pone.0237402)
Supplement: S1 File — (ZIP) [file pone.0237402.s002.zip › manuscript data/FGD Kayabwe 2 Eng.docx]

**KAYABWE FGD**

M: Then it’s us that are always making women pregnant, then you want to know..….

P: Then she tells you that let’s both go to the hospital and get tested.

M: Yes, let’s start from right there now, how do you feel about getting tested as you men?

P: It’s a good thing

P: It’s not bad a thing

M: First wait so we can hear from one person at a time, as we come; let this gentleman tell us now

P: It’s good to know your status so that you can maybe get treatment early, instead of waiting out for the disease to grow in your body. What you are saying is right sir.

M: But the voice is so down. Come closer sir because we want to pick the voices

P: I’m requesting to ask, I’m asking that if I test for HIV and I’m positive and I’m given treatment, does it come a time and I still die of the same disease?

M: You die of something else

P: It doesn’t kill me?

M: Actually on that note, we shall talk about it at the end because this gentleman has a lot to talk about. If it comes up somewhere in the middle, then we shall talk about it.

M: Do you know that today it’s different to have AIDS (the disease) and testing you and they find that you have HIV virus in your blood. A person with HIV virus in their blood and a person with AIDS, do you know that they are very different?

P: I knew it as being the same

M: No, being tested and they find that you have HIV in your blood do you think it is the same as having AIDS?

P: Now mum there…

M: Let me ask, I’m asking a question. I’m basing on the question of this gentleman

P: Hhhhmmm, me I say it’s not different. Me I say that it’s the same

M: Why do you think it’s not different?

P: AIDS and the virus that brings AIDS are not different

P: AIDS and the virus that brings AIDS

M: Being with the virus that brings AIDS in your blood and being with AIDS, do you think it’s the same thing or it’s different.

P: That’s what I want you to explain to us

P: They are not different

P: Me I say I see a difference there

M: Hhhmmm what difference do you see there because we have two types of things

P: Because it’s good for someone to know. Off topic conversation and rearranging seats to accommodate someone

M: Ok now that thing in was asking you, why is it different and why is it not different? Rearranging again…………

M: Hhmmmm

M: Talk please sir

P: Me where I see that it’s different from; the other is a virus in the blood and on the other end, you are sick. The other simply brings the disease AIDS.

M: Then it leave and go!

P: Does it go out! It also remains there, because it’s in the blood

M: Others, what do you think?

P: Me I don’t see a difference.

M: What I know is that everyone has a thought. You say out your thought.

P: The virus that brings AIDS and AIDS; I don’t see a difference.

P: When it’s you who has them?

P: Yes, when it’s you who has them.

M: If they test you and they return your results when you have the virus; what do they tell you? They tell you that you are HIV+. They tell you that you have the virus. Now, having the virus in your blood is it the same as being with AIDS, the disease AIDS?

P: There I’m confused.

P: Me, I completely see that they are all the same. If they tell you that you have the virus that brings AIDS. I completely see that the thing is the same. It’s all disease automatically.

P: Me I’m requesting you to tell me one person that has only one of the two. When they don’t have both

M: When they don’t have both?

P: Eeehhh when they have only one

M: For you do you think that it’s possible to have both? Or it’s possible to be there with only one?

P: It’s not possible, still you have to have them in your blood, or you don’t have either.

M: Hhmm, what comes first, let’s first go back to these two things what comes before what?

P: Because mum………………

P: You start with the virus

P: Mum me I have raised up

P: We have one problem, we don’t understand what you are talking about, ask how

M: How?

P: Now here when you say AIDS and the virus that brings AIDS

M: English differentiates them; the problem we have is that our language Luganda is narrow. But you remember that in English they say HIV+, meaning that you have the HIV virus. Then remember that they write HIV/AIDS, which is AIDS, the disease

All: it’s the disease

M: That’s why I ask, If after testing you and they say you are HIV+, is it the same as saying that you have AIDS? Do you think it’s the same?

P: yes

P: aah aaah

M: now that’s what I’m going to base on to ask. What is AIDS, for us that we call silimu in Luganda?

P: for me let me first seek for clarity, it seems like saying that you have the virus that brings AIDS I think that we should call that virus being unfaithful.

All: aah,aah …………laughter follows

P: there are those that got it through breast feeding

P: there are those that are born with it

P: Katerega tell us what you think

M: wrap your thoughts around it

P: ZZ

P: who is ZZ

P: That’s him

M: ZZ is hiding; he should come and sit here.

P: What is silimu called in English?

All: AIDS

P: How about the virus, what is it called?

All: HIV

P: Eeehh, eehh, they are all called AIDS, even HIV is called “silimu”

M: That’s in English, for us in luganda we say “akawuka”. The other disease we call “silimu”. For us we say the virus that brings AIDS, just as you would say for malaria that a germ that causes “omusuja gwensili”. For us we use “kawuka”

P: I’m requesting that you enlighten me on those two things

M: Yeah, also me, you were giving your thoughts and I returned it.

P: We are defeated

M: It can’t defeat us, all these heads

P: Let’s put together all these heads

M: Let’s put together these heads and leave when we have shared our thoughts. These kids

P: We have to differentiate them, all of us here

P: Hey desire you go you go

P: We are defeated

M: Let’s put our brains together on this matter

P: This matter has defeated us

M: Hhhmmmmmm

P: Because

P: This matter will not defeat us

P: This matter will not defeat us, why I think that it won’t defeat us, that virus, where does it come from and where do we get it from, isn’t it in cheating? After getting it what do you spread?

M: I will ask a question at that point also, where do we get the virus, is it in cheating only?

All: aah,aah,aah……………..

M: Where else do we come back with it?

P: At birth

P: Needles

M: The one for needles is there

P: I could use a razor blade

M: The one for the razor blade is there

P: I could get an accident and I get in contact with someone who has it. Blood could mix or wounds could get in contact.

P: Truthfully where does it come from?

P: We are still thinking

M: There is one way that I’m waiting for that you are not looking at. When you go to the salon, we use the same machine

P: One that cuts RR head also cuts mine

All laugh……………….

M: Now I think we can see where we are heading

M: Ok, since it’s that all of you cut off beards, should we say that you all have the virus that causes AIDS? Because we said it can go into the hair as well and we all cut off hair.

P: Then me I ask that where does it come from

P: In the blood, in the blood is its life

M: This one is asking if it can pass through saliva

P: Wait a bit, you can get a woman with AIDS and you don’t get AIDS. But in case she gets a wound. You can get it. As long as the blood mixes

P: So what you are saying is that you can go ahead with kisses? Since we are sharing thoughts

M: Hey guys, answer him

P: You are allowed

P: It’s your decision

M: Facilitator laughs; you are answering that unsurely

P: Go ahead with your kisses

M: The way they are telling him that it’s upon him, there is a way they said it.

P: For us we showed him one thing as it happens. That AIDS is transmitted through exchange of blood. You can even marry a woman with AIDS and you have sex with her and you don’t get the disease. But if you get bruised, that’s when HIV is transmitted.

P: As long as one of you pours blood

P: Saliva is just water; AIDS can’t get you through it.

Facilitator claps …………….

M: Ok now let’s go back, now we have gone back, being with HIV is it the same as being with AIDS? Remember we looked at this thing and knew that even if our language is narrow, the English differentiated it fully. Having this virus is it the same as having the disease AIDS?

All fumble: doctor, doctor,

M: Let’s listen

P: Ok, let me ask you something; If I get the virus that causes AIDS, when it’s in my blood, can I not get AIDS?

P: I’ve also raised up

P: Me I’m also asking a question

M: You would have first given him an answer, because he asked a question. We should all first wrap our minds around this. Don’t first ask me. We are all here to learn from each other. Because I also know some things that you don’t know and you also have a lot you know that I don’t know. But the most important thing here is that we all think about this. We shall leave when each one of us has added something onto the other. That’s the truth.

P: Me I’ve just come to learn

P: If I get the virus that causes AIDS. Could I not get AIDS? Could I get it or fail to get it, when I have the virus. Is there a way I can be helped so that I don’t get the AIDS when I have the virus that causes it?

M: What do the rest think, what do you think?

M: Yes, sir

P: Because he doesn’t have any chance of not getting AIDS

M: Wholesomely for you see that he is going to get it?

P: I’ve also raised up

M: Yes

P: Me I’ve heard even if you go for testing, my friends, now doctor, If I went and tested and found that I have HIV, and I straight away start on medication, can I get prevent AIDS?

M: That’s also a question amongst us, for you what do you think, because another mater has been introduced, did you hear how he brought it up? He has brought in the matter of medication. If someone gets tested and they are found to be HIV+ and they are given medication, how does it help them?

P: It helps to strengthen you, but still you end up getting it

M: What do the others think

M: There’s this gentleman

P: Me I say you have it… but when you are getting treatment.

P: Yeah

M: Yes, sir

P: Well for me

M: Well, you can be tested and they give you this medicine

P: Which soothes the virus

M: Yes yes; do you hear how he has brought it out, our medicine that we have does what? It soothes the virus, is that true or not?

P It’s true, it soothes the virus.

M: It weakens

P: It soothes all at once

M: Now if this medicine soothes the virus, just like this gentleman has brought it out. They have tested you and found you positive and they have given you medicine to sooth the virus, do you get AIDS or not. Wasn’t that the question?

P: That’s the question

M: Its back, we are all asking questions, now let’s put our minds together

P: You be having it

P: You be having it yeah.

M: What did you have to be given this medicine? What did they base on to give you this medicine?

P: It’s the virus, but when they give you that medicine, it helps to prevent you from spreading that virus to someone else. Me that’s how I see it

M: What have they based on to give you this medicine?

P: The HIV virus

M: Yes, then they give you the medicine to do what?

P: To prevent

P: To sooth not to prevent

P: Ooh

P: Not to prevent, not prevent

P: Already you have it

P: Already you have it

P: Is it going to go out?

M: You have the virus, they have given you medicine to sooth it

P: To just anesthetize it

P: To anesthetize it

P: What means

P: It weakens it

M: Uunnhhuu

P: To reduce its growth speed

P: If you don’t take the medicine you get AIDS, if you swallow the medicine, it means you won’t get what they call AIDS, you remain with the virus

M: Is that true or false

P: It’s false

P: It doesn’t exist

P: Now what would you have

P: What’s the other

P: The other is a virus

P: They are twins

M: First listen, remember where he started from, before you point at that idea, this gentleman brought it out well

P: I have another idea. If you are the eldest at home and you don’t produce children, do you think kids can come? It means that the virus gives birth to AIDS.

P: It gives birth to AIDS, which means that when you get it, you have AIDS.

P: When you swallow the medicine does it die?

P: Let me ask this please gentlemen forgive me. If you are writing ten, isn’t it written like this? Now if you don’t write this can you get this. Without writing this can you get this? This means they all go together.

M: No

P: They are all twins, is that what you are trying to say

P: The approach you’ve brought it in doesn’t have health in it,

P: It has it

M: That’s mathematics

P: My friends, here is my thought, Silimu, is what language

All: Luganda

P: How about akawuka

All: Luganda

P: AIDS is HIV

P: No sir

M: Let him finish his thought

P: Silimu came from Luganda and went to what as you think?

P: AIDS

P: No, AIDS is English

P: Now AIDS is also Luganda

P: AIDS isn’t Luganda

M: AIDS is English, silimu is our language

P: Why are the two things combined

P: Every nation has its name, the Rwandans say “akakoko kasida”

M: Every nation named it its own names, just like for us here we have ….

P: Musawo, I’m requesting, this group I’ve come in or where we are seated now, me I know it well, the night will fall and we shall sleep here.

M: As long as we understand, we came to share thoughts

P: I was requesting, that now like you, me I’ve been tested for like 15 times, and every time I come out negative. But now this thing that HIV, that the virus which causes AIDS that I have it. Truthfully I’ve never heard of it and this is the first time.

P: Yes

P: Yes

P: But if we’ve discussed it and you’ve brought it up, let it have like four or five signatures, then you tell it to us and we finish it, then go to something else.

P: Then we go to something else

P: Because you have more knowledge than us

M: No we don’t,

P: Hehe, we can also be more knowledgeable

M: You also know it

M: We could just tell you and you don’t understand, but here when we are sharing thoughts, this is where you understand

M: It also helps if someone hadn’t understood something

M: This gentleman had a good idea that we didn’t pay much attention to. But I don’t know if he still remembers the way he brought it out.

P: I gave it to you

M: Well, you repeat it for the others

P: I was around and I didn’t pick it.

M: At first he said, you first have the virus, and then they base on the virus to give you the medication that soothes. After that then this gentleman got up and explained what happens when you swallow the medicine. Yes, what did you say?

P: Iyiii

M: Me I was very attentive my friend

P: He asked you that after getting the virus that causes AIDS, if I have HIV, if I’m tested and I have HIV, do I have a chance of not getting AIDS when I get the medicine

P: Yes

M: The answer has come out, why yes

P: As long as you test before it spreads through the body

P: Does it cure?

P: It doesn’t cure

P: Does it leave

P: It remains.

P: It’s like sleeping. The medicine makes it to sleep. The medicine you get is to make it sleep.

P: So that it doesn’t move!

P: So that it doesn’t get out!

P: So that it doesn’t reproduce

P: Could it wake up and start moving?

P: The medicine you swallow

M: If you miss and maybe don’t swallow. It’s what he means

M: Now before you make this gentleman tired, you see we don’t want him to get tired yet you called him knowing that he has your good ideas. For you what do you think? First conclude on this matter. What you will talk about is what I’m also concluding with.

P: About that matter, me I had never heard about it, because I know by the time they tell you to swallow the medicine, you are sick.

M: Yes

M: It’s just because our language is narrow, you see. If they test you and tell you that you have the virus. Having the virus, they tell you that you are HIV+, because that HIV is there in your blood. It’s positive because it’s present. It’s like saying HIV present. HIV- is HIV absent. It’s the same thing, it’s not there. If HIV is present, it is in the blood and reproduces, the more it reproduces, it slims you. That sliming, falling sick and you are down when your Immune system is all done because HIV it’s up the system so that it can reproduce and grow. Then you remain out of shape like that, any disease that comes gets to you, cough can get to you, herpes, etc. that’s AIDS and that’s the situation. But if they test you and give you the medication this one talked about, remember it soothes. If it soothes and weakens this virus, it prevents it from reproducing and it becomes dormant. Which means that a person can exist with this virus but when it hasn’t reproduced which means it hasn’t eaten up their Immune system to see that he won’t get other diseases. And he exists properly and looks better than you who has been tested 70 times when you are negative. Because you should know, when the virus is in your blood, if we look at today what we have, we don’t have medicine that can kill the virus, but we have medicine that weakens it then it remains there to the extent that unless you wanted to tell people that you have it, only those you choose to tell will know but when nothing shows, you see it. But when you have HIV and you take the medication to weaken it, it’s not the same thing as having AIDS, you could have this virus and you don’t get AIDS, you could get this virus and get AIDS. It depends on what you decide to do. If you decide to take the medicine, we’ve talked about what the medicine does, if you decide and say I have the virus but I won’t swallow the medicine, also what it does we’ve seen. You be going the other side. Which means that today these two things are different. It requires us to be able to differentiate them. After we’ve been able to differentiate them, everyone can go back to their place and decide for themselves. We came to share ideas. Whoever has a question on that matter?

P: You can make the virus barren

All: Yes

M: Maybe as I conclude what musawo was saying, there are people who have that virus, you know them as she said. They regain and look better, and then the next time they test them they come out negative.

P: Because of the other medicine

M: When they can’t see it, remember it’s dormant, it’s not reproducing

M: When it tries to reproduce, the medicine stops it, and then the person looks healthier and healthier. Then for you you come and ask to sleep with them.

P: That’s true

All talk…………

P: They bring a thing and prick here, then check if you are positive or negative. If you are positive they tell you to start on treatment immediately.

M: The reason why they tell you to start the medication immediately; the other virus they found to be positive, they want to deny it the chance to reproduce. By the time they say positive, when they have seen it, it means it has started reproducing and they have seen it in the blood, but the more it reproduces it does what! You could be looking better at the time they discover it just like you look now but the more it reproduces, it feeds on you, it’s like how you see maggots, it eats to finish.

P: Well those things are not explained to us

M: Well we are here now

M: Ok, now it’s you that brought out the ideas that’s why it has come out.

P: They just test you and then give you the medicine.

M: Now that’s the importance of that medicine. That’s why you see they brought that medicine. Even if it doesn’t kill it, it prevents this

P: Musawo, the topic here

M: First wait, let’s listen to this gentleman

P: We are not arguing about it,

P: What’s the main point you are talking about now.

M: The virus

M: Sharing ideas about the virus

P: Ok now we have shared the ideas but what’s the main point you are giving us.

P: What advice are you giving us

P: What advice are you giving us as health workers

M: Well now, when we come and share ideas, get to know your thoughts and we have also come with ours. Right now we are still sharing ideas.

P: Yes

M: After we are done sharing, you yourselves will leave when you have understood everything. The other gentleman reached the other side but he was the first to appreciate this gathering that he has picked something.

M: Before we close that topic we’ve been on, have we all differentiated this thing of having the virus and having AIDS

All: yes

P: I’ve understood that one

P: I’ve differentiated

M: Who can, recap it for us in our language

P: In our language?

M: Yes,

P: They told us like this, if you get HIV, and you act first and go for testing; and then you get medicine……………….

P: That stops it from reproducing

P: That stops it from reproducing, it helps you to live well and even live longer

P: What you mean Is that prevention is better than cure

P: It’s like it doesn’t reproduce to multiply in the body. You only be having the other one. And you make it barren, it doesn’t reproduce.

P: You just make it lame

P: You silence it just

Some laugh

P: It remains there and you only fight with that one, but if you fight with like 10 or 20 viruses, my friend.

P: But when you are taking the medicine……….

P: Yes, when you are taking your medicine

M: Yeah when you are swallowing your medicine. Because today, if God helps us and we live for like more 50 years, as science progresses, maybe one day they will discover the medicine that kills it. But today……………..

P: Musawo, what you said I interpret it like this, I know a gentleman; he is in this village; he fell sick; we were with that gentleman. I was surprised to see him today, he was here in 1989, or it could have been around 1986, but he was so sick like almost dying; he was an army man. But I saw him today when is alive and well and looking good.

P: But was he suffering from HIV.

P: He was down almost dying, he was an army man

P: Don’t go very far; I came here in 1987 and people used to say that Moses was ill with the virus, but now he even looks better than me.

P: He looks better than us

P: He looks better than you who is healthy

M: My friends have we seen the power of medication? Have we seen the power of medicine?

P: 1987 but even today he is still alive

M: Have you seen the power of medication? Before we even leave this matter, what I know is that we’ve now all agreed.

All: yes

P: That one we have differentiated

P: We’ve differentiated that one

P: We’ve differentiated that one! Even the importance of medication we have differentiated?

All: yes

M: even the good things about it we’ve found out?

P: yes

M: now, before we leave that matter there is another issue. If you are tested and they find you HIV+. You; today we have men; you as a man when it’s you with the virus.

P: me?

M: yes, the wife doesn’t have. We are looking at men because it’s us giving the ideas. We’ve first put ourselves in the worst position. We shall also change the other side and also put the women. Let’s begin with us because it’s us giving the ideas. When it’s you the man with it, the wife doesn’t have it. But at the moment they found out your situation, you have and she doesn’t have, you don’t have children. Do you think you can have children?

M: Let me start from here musawo, with this gentleman. Did you hear what musawo asked?

P: Yes, I heard

M: A bride, you’ve decided this time to get a bride, but you’ve brought her, she’s negative.

P: If that where you had started from, we would have even left

M: You talk

P: When she’s a bride, he just married her. She hasn’t given birth before and you want to get children. What do you do?

P: And she has found out that you are positive

P: What do you do

M: And you still want the bride to produce children.

All Talk……….

M: Sir we are listening to you, the bride has come, she has found out that you are positive.

Off topic chat

M: Let the gentleman give us his thoughts

P: ok if the woman is the one, who is positive,

M: no, it’s us who are positive. We started when it’s us the men who are positive

P: the man is the one positive

P: and you’ve brought home someone’s daughter, because producing children is essential and even if you are sick you can produce children. What to do is that; if earlier she had produced like at least two kids I can leave her alone and we raise those ones.

M: no we are looking at a bride, you’ve got your bride, married her,

P: she hasn’t produced

M: she hasn’t produced; she came when she is negative and yet you are positive.

M: and you’ve decided this is the mother to your children and it’s assured you want the children. But now you’ve been tested ……….

M: let someone else share their idea. Let’s go to your neighbor

P: that thing, to be when I’ve got a bride and I’m positive and she’s negative

P: you can’t chase her

P: you can’t chase her. And you won’t tell her that you are positive, but in my thinking, after I’ve discovered that I’m positive, I find a way to tell her that I’m positive. Because what I know is that you health workers you have a way of doing it when me I’m positive and for her she’s negative but we can still have children.

P: and she allows!

M: let’s go to this gentleman and hear his thoughts. Because everyone gets it on their own

P: me as me I don’t tell her, I take my medicine

P: and you go ahead to have children

M: we left the bit of taking medications; we already know the importance of taking medication. But what’s in front of us now; this is a bride. You have her already, you know the importance of medications and you already have the virus but you still want the children. And this is the bride that you have chosen. You want the children. The other gentleman has a view

M: no, we are coming there. What do you do?

P: I don’t tell her

M: how about you, sir

P: me I don’t tell her, however I go and get the medication, if it comes out that, maybe when she has gone for antenatal care when she gets pregnant, or she has gone to Immunize a child, and they get her blood and test it and discover that she has it. I tell her that we are all just starting.

M: what you are saying is that you transmit to her?

P: yes

M: this man is heartless

P: someone’s daughter and spread to her

P: I just fire her bullet’s

M: you just fire her bullet’s

M: let’s go to this gentleman. You’ve brought your bride this week, and you love her, but you got the virus long ago. Yes, let’s hurry up. So many ideas

P: In my opinion what I do is to tell the bride to go back to her home.

M: even if you love her

P: yes, and I die of my own disease.

P: even if you love her

P: I swear even if I love her, now should I kill her?

M: why do you call it killing yet we talked about this at the beginning.

P: then I keep quiet. If the medicine was allowed then I keep quiet and swallow my medicine when she doesn’t know at all

M: then you spread to her!

P: what should I do now

M: you see why I brought up that thing, let’s first get all the opinions and we exhaust them

M: yes sir, how do you do it

P: eeeh, I see

P: me what I know, if I love my person and I don’t want her to leave, if you travel, you acquire knowledge. But I know very well, on my own I know, because I even have a friend of mine who does what! I get her and take her to more competent hospitals. Then I explain my situation that I’m what? I yet I need to do what;

P: to produce

P: now, me what I know, those competent hospitals; you know them, they can find a way for that woman. They have things they can put in her that maybe can protect what! And we produce children when the woman has not got any what? Any disease. Then me I remain with my disease but when the woman doesn’t have what? Because she has something that prevents her. In competent hospitals they have it I know it.

M: if they ask for money

P: when you don’t have it.

P: iyii, just money

M: Now that woman, you’ve brought her…………

P: Me as me, I don’t tell her, but if it’s her that’s positive and I’m negative,

M: No, we started when it’s you who is positive, we are the ones on the bad side

P: If it’s me then I don’t tell her. Did I just breast feed it?

P: You’ve spoken the truth

M: Yes, sir

P: You go ahead

P: Me my elder what I do, on that matter

P: When it you who is positive

P: When it’s me who’s positive, the man, it’s me who wants to reproduce, I’ve brought a young bride. Ok it’s me with the virus, that virus for me I will have to swallow tabs for it as I have sex with, until we get a child.

P: You stop me, me just recently they had given me a wife. When we went for testing, they read wrongly and I was positive. No the woman, at that very moment she changed her mind. And she told me boss……

M: Eeh they had just got her for you

P: She was mine, we have spent some time but it’s not for having sex right away, you know how it goes like that. That’s when I said eeh, you have killed me. Then I told the woman, my friend me I’m positive, what are we going to do, the woman told me there and then that my friend you know what, let’s keep the friendship. You will kill me for nothing. Now ma’am, for example musawo you are there, you are a bride.

M: For you you’re sick, and we brought it as bride why, you’ve decided that sincerely this is the one who should give birth to my children. You’ve decided!

P: yes,

M: that’s the situation,

P: yes

M: Hhmm? And now you want the kids

P: Musawo I love you and you also love me, we’ve gone to your home, we’ve gone for blood check up

M: You’ve even put in your money you’ve brought things to introduce me at our home. Now imagine

P: Musawo, then they tell you, that man, you see, that man is sick, musawo you, will you go ahead with me.

M: You’ve not yet told me, it’s you who knows

M: For us what we want, you’ve introduced musawo, you’ve put in all your money, everything is about to go down, you’ve brought her home. You’re saying she’s the one you want she’s the one going to produce my children but you are sick.

M: You’ve been tested and found positive, mugole is negative as for the kids, you want them. What do you do?

P: The truth is like this, let’s not pretend.

P: Let’s leave this issue and the basawo tell us and we go to something else.

M: He is bringing it out, these thoughts by the way it’s you who always have the answers

P: There is no one here who can say that you will tell your wife.

P: I cannot tell her

P: There’s no one here who can do it

P: They will be lying

P: You even if she’s a night dancer she will first leave you then people will tell you that she has been a night dancer.

All: talk,

P: My friends I hope suggestions are over, let’s give the chance to the basawo and they tell us. we’ve all given our opinions

M: Mr. SS what do you say, I see you are also a male

M: You see for me what I do, I go back to the health worker who tested me. And I tell them musawo, what should I do, the bride I brought, we had not gone far, but I’ve been tested and I’m positive, the bride I’ve brought is someone’s daughter who seems to be negative; she’s negative, let’s take it that she’s negative. What should I do, advise me to see that she knows that I’m positive without me telling her because I will also be afraid.

M: And you don’t want her to go!

M: And I don’t want her to go.

M: So, musawo is the one who will tell me that you go and bring her as if you’ve come for treatment, for you to tell her it’s you who is sick but escort me. Then we go to the hospital, then she will test us and she will give us new results. When she gives us the new results, that’s when I know the truth and I find out what she thinks about it.

P: Won’t you be like reporting her that she

M: No, we’ve been tested and it’s me who’s positive, for her she’s negative

P: Musawo? Well, assume I’m the health worker; you’ve come to my place

M: Yes

P: For the second time

P: Yes, you’ve come with your wife, what do you think I will say, still I will say if you want the woman, you use the condoms.

M: Well, yes, still it will be musawo giving me the advice.

P: Yet you want to get children

P: There you will not have children

P: Reproducing is still in the picture, where we started from my friends first remember, we said having the virus isn’t the same as having AIDS, which means that this person with the virus can survive and live for really long. Ok let’s say, you’ve got it when you’ve just become a youth at 17 years. You’ve been with it and gone through whatever you’ve gone through. But when you make 30 years you say will I die without a heir! You’ve got the bride; you’ve gone through whatever you’ve gone through. You’ve decided and said, let’s say you were even responsible, you’ve gone there and introduced. On introductions no one is forced to test, now you are at a point where you want children. You should know that this is the person you want. And remember we said it’s not a crime to have the virus and we differentiated the virus and the disease. Even the way we talk should be different now, a person with the virus isn’t sick; but has the just the virus in their blood but not the disease. We differentiated the disease and I hope we are all clear on that. Now this issue of children is what we are looking at.

P: The truth, a woman you’ve introduced very well, you don’t tell her there and then

M: But you want the children

P: You get strong

P: A woman, if you make her pregnant ………

M: What you are trying to say is that among all of you here if one of you found out that they are positive, even if they have a bride, does it mean you won’t produce a child on this planet

P: You tell her later

P: You mean, you give up on the children

P: All these things are difficult musawo, for me I may keep quiet but even those young girls she can come when she knows that she’s positive and on drugs but doesn’t tell you. Unless you check through her things when she has gone somewhere

P: My sister

P: And you find out

P: I have a sister of mine

Random talk

M: listen; listen to him

P: I have my sister; she told me she got HIV from a man when she was 14 years. She was the first to sleep with him. Now she has 5 children, each with a different father for most of them. She spends like 3 to 4 years with each man. But she told me that no man knows that I have HIV.

M: are the children positive or negative

P: the children are negative

M: how comes, that’s what we need to know today, everyone needs to know that even though I have this virus, it’s possible to have children and when they are negative. We need to know that thing. Has your sister explained to you?

P: yes

M: how does it come about

P: she told me that she goes to the hospital and they get her blood sample, she swallows her medication; then at the point of delivery, the health workers help her and she delivers well, her kids when they make 6 months she stops breast feeding them.

M: for the woman for them they go to the hospital and take the medication, now we are looking on the side of the gentlemen; what will they do?

P: when it’s me who is sick?

M: it’s you who is sick

P: automatically I’m going to do what the woman has done to me. When she gets pregnant, eeh,

P: that’s when you tell her

P: that’s when you go together

P: not really, she can get pregnant when she doesn’t have HIV,

P: when she doesn’t have

M: well, my friends do you see why I’ve put emphasis on this thing, do you what he has described, in most cases when we think about these things, they favour females. For the woman you see that they have something favoring them as them. But also you as a man, you have equal rights to get what you want. Why the woman lives, you’ve explained the that very well, for her she knows, let’s say your sister, for her she knows and she also has her children, now that’s where the situation comes from, when it’s you who is sick. Does it mean that you seal yourself?

P: I have to have sex

M: does it mean that you seal yourself?

P: I have to have sex, and even getting the virus, I’ve known that it depends on the nature of the woman; she could be sometimes dry, and such things.

Some laugh

M: we are going back, which I don’t want

P: we are mature people my friends.

P: don’t take us back where we delayed to come from

P: I can be positive, or the woman can be positive and I have sex with her and i don’t get the virus.

P: it’s not taking you back; we are still on the same topic.

P: you reach there when your libido is all high

P: you just go on bare soil

All talk……….

M: Mr. SS you conclude this thing

P: what you mean, if you are positive, whoever you be with you spread the virus to, no; the woman may not get the virus but when you have it

M: if you do what?

P: eeh

M: if you do what?

P: after all we are mature people, as long as you romance yourselves well.

M: wait, now I want to ask you properly

P: yes

M: remember you told us

P: yes;

M: your sister, the very first husband spread to her, she was not cheating!

P: yes

M: you told us he was the first, me I’m only looking at that. Ok now, what you mean you can have sex with someone and they don’t get it. I want us to look at this thing clearly.

P: everyone that spreads HIV to another just rapes them, there’s no need for feelings

P: me I say, we be with women, ma’am what you are saying …………

M: there’s, a point you were bringing out, the one about preparing someone, you were still talking when you got interrupted. Yes what do you do?

P: with romancing, start from there; because it’s where you stopped and you know it’s where you stopped.

P: when there is no hurry, you be there and you are mature people

P: when I’m the woman, I’m the one with HIV,

P: it’s you with it, or it’s the woman with it. You’ve slept with like 3 women without testing; you mean all the women like ten are all positive

P: they are all positive

M: ok, you get to the point

P: it means you can just go in without protection

P: me I know something like that, if it’s the woman with the virus, and me the man I don’t have. Chances of me not getting that virus are many, as long as you give her time. And you perform in your right time, and when she has all the requirements, not these dry women. I mean when you like the thing is coming out well. Me I see that I have a chance there but when it’s me the man who has it. Those feeling are hard.

P: you could be when you cut your pubic hair and she also cut.

P: so whoever you find as long as you romance well

P: but if a woman is the one with it and she has all the requirements well, she handles her things well and you find trouble with it, me I see the chances of not getting the virus are there.

M: gentleman what do you say about that matter. She has, this one doesn’t have

P: that matter, I talked about it, and I said that you health workers are the ones who can give us advice and say that if I’m sick, I cannot tell the bride

P: yes, I support you on that

M: this one also talked about it

P: I was the first to talk about it and I said that you the other medicine that you give us when it soothes.

M: well, we’ve been giving each one a chance to talk

P: for you you were absent

P: I talked

M: we were asleep,

P: we were on something else,

M: Yes and now we are no this thing of spreading and not spreading. We want to finish it and go to something else.

P: there’s something we’ve not talked about

P: this is what you found in amidst us

M: first wait, let me explain, for those that didn’t hear

P: he didn’t hear now we are going backwards

M: no,

P: the main point was like this, you the woman is sick with the virus and the man doesn’t have and I talked about that matter; I even gave an example about my sister.

P: that’s what you found amidst us

P: we went through all of it

P: well, let them bring another one

M: we can say we’ve concluded that one

M: no, we are going to conclude it

M: yes

M: that one in English is called discordant couples; this one has, this one doesn’t have; Like this gentleman talked about it. All the thoughts you’ve given about this are all right. Do you hear me, like the gentleman said; you have to prepare your partner. So that you don’t be travelling from Kayabwe while going to Kampala and your partner doesn’t even know that you’ve boarded a taxi you are going. You have to do what, you have to let them know; let me give you an example. I have a friend of mine; when he comes to work, we talk a lot, but sometimes he takes me aside and says, my wife as I was leaving home today, I didn’t understand her, as I was leaving, she held the bag for me then as we arrived somewhere, we had got the boda-boda and she refused the boda-boda to leave. Then she began playing around. Then I asked him, did you quarrel, and he was like no, I was just surprised. When he comes back the following morning and you ask him, in what mood did you find her, she was happy for me. She even knelt down, welcome back dear; she could be sending a signal. There’s also a man who arrive when they are home with the woman and she begins setting traps, when he puts something, she removes it; eeh, and they get that joking mood. The brain, will be started, one prepares the other to go to the other thing. It’s what this gentleman was saying. Now I hope we’ve resolved that issue. By the time they reach to the other journey, it doesn’t feel hard. Remember we said that getting HIV, this gentleman explained it.

P: it’s from the blood

M: so, let’s conclude it like that

P: I clap for you.

M: Which means, you can even produce children if you as a man you be what; you be when you’ve swallowed the medicine. Because we faced it in ourselves because the discussion today is we came to discuss when they are in the favor of a man.

P: I also have four of them,

M: the kids, all the kids are negative.

P: Yes

M: ok, how about you

P: I’m also negative

M: ok, well now let’s talk about the importance of testing we’ve talked about everything, where it starts from, how it gets to us. This time I think I’m going to start from here then I come there; everyone should give me one importance of testing for HIV. Yes, sir

P: me before I tested, I thought I was positive and any woman I would find I would just sleep with.

M: ok, that’s one, yes sir

M: they shouldn’t give us the reason of knowing my status; because normally there’s a reason except knowing your status. Everyone wants to know their status, that’s a reason for everyone but everyone has another different reason which isn’t knowing their status.

P: you go ahead

M: sir, go ahead

P: now for me, depending on the current status; it would be important every after a month or two for someone to test and find out their status, if anything went wrong you rectify it.

M: thank you; yes sir

P: me I don’t see the importance of testing,

All laugh ………………

P: because even if I get the medicine still end up dying

P: no no no

M: let’s assume

M: he has a very important suggestion by the way. The reason of not seeing the importance of testing for HIV is very important, very important. Why don’t you see the importance of testing?

P: even if they give me the medicine, I swallow it, then I get sick and I die

P: it’s like a calendar!

P: it’s like a calendar, if you will die on 20^th^ 2002, you will die on 20^th^ 2002

P: ok, thank you, let this gentleman retaliate a bit

P: the reason why you swallow the medicine, and testing, you’ve been told that you will prevent the following things; cough, diarrhea, there’s what they call herpes, swellings and even living a healthy life

P: what you mean you’ve never seen a person who has lived for over 30 years with the disease

P: we said, Nick came here in 1987, but there’s someone he found positive and they are still alive and well today

P: I told you, whoever will die on 20^th^ 2002 will die on 20^th^ 2002

M: thank you, he will understand slowly. Yes sir, give us one importance

P: me my importance, when I go and test like this, it teaches me going forward; that the women in my past have not spread the virus to me and I don’t hate them much but these ones with their curves that I’ve not seen, I hate very much.

M: thank you; yes sir

P: me that thing, of testing, it’s very good. Because for me at my age I’ve tested for like 15 times. Me I see I be keeping my life as I remain knowing my status.

M: yes sir

P: the thing about testing

M: the good one

P: the good thing about testing; I easily find out what I got from wherever I’ve passed; after knowing that, then I know the state of what I got from there. Is it good or bad; it’s bad. If it’s bad, how do I go about it? Also other people not to know about it, even my sister

P: I have over 40 years but I’ve never tested

P: you?

P: yes, 40 years, I’ve never taken any medicine, I’ve never been injected,

P: then you could be positive

M: wait wait

P: first leave us

M: yes sir

P: what I see about testing

M: a good thing

P: a good thing; ok if you have a strong heart, you could be helped and you swallow medication. If you are strong. But sometimes when you test, what may cause you to die early ….

P: stress

P: stress

M: I knew you were about to bring it up

P: the heart gets weak, and we’ve lost a number of people here who have tested. They test and then die soon after wards when they are very afraid.

P: fear

P: it requires one with a strong heart, but it would have been good; although some people really get scared and they die soon after.

P: I also have a young brother who tested positive but when he was given the medicine, he swallowed over dose.

M: yes sir

P: the good thing about testing; I won’t differ very much from the other gentleman. This testing, if you are found positive and you start the medicine immediately. You prevent these other diseases like herpes.

P: how come when they come to test you, you run off

P: who?

All laugh …………

P: were you the health worker to test him

M: yes sir

P: well, for diseases like that, herpes, suffering from STDs, diarrhoea,

P: and knowing your status

P: and knowing your status

M: thank you, yes sir

Random talk ………….

M: please let’s keep quiet and listen to the big people this side. Yes sir, what’s good about testing?

P: me on my own, the important thing about testing, it helps you to know yourself and you know if you are positive and you swallow the medication. Because what I understand most; the other gentleman said that for him testing causes him pressure and he may lose his cool. But for me it makes me brave. Because I can’t go forward and put myself in an accident after being shown that where I’m going it’s there.

M: yes sir

P: even knowing, you need to know

M: major general is here, he has something

M: how about this gentleman

M: yes sir, bring your opinion. What good is in testing?

P: the good thing in testing…………

M: please listen to the general

P: the good thing about testing is knowing what you are, then you prevent or you go ahead

P: now general, after knowing that you are positive what do you do

P: you swallow medicine

P: if I know that I’m positive, if I know that I’m positive, I become careful of what I’m doing

P: I thought you just go ahead

P: no sir, I become careful,

M: by the way on that issue, I want to bring another question. What problems do men find in testing? Because on that very issue, it takes us back to what the other gentleman also said. Because one question can ask what problems men find in testing or what bad things they see in testing that prevents them from testing.

P: not wanting to know their status

M: before you say it, before. Health workers have moved in many areas, even if they came here under that mango tree. You are always the first to ask. Even as we came to park here, you were the first to ask, what they want and we heard you while in the car; what do they want. Then one came and said they have come to test; then I started seeing some disappearing behind corners and yet they were men. But now what’s the problem? What prevents us? This time where should I start from; let me start with this gentleman and then come like this. What problems prevent us?

P: the problems that normally prevent the men, fear

P: fear

P: they are very fearful, most of them

M: the reason of fear is a big one, what exactly do they fear? What do they fear?

P: knowing their status

M: what knowing status are you talking about; you mean you don’t want to know your status. What else do they fear?

P: if he goes and gets tested, if he has been a farmer and he usually digs from here where we are seated to the other eucalyptus tree. He may this time stop there; he doesn’t reach the other bottle.

P: he doesn’t reach the bottle

M: but what?

P: sickness

M: so what you mean is that they are afraid of finding out the truth, they are afraid of knowing that they have the virus. What else are they afraid of?

M: let’s come here sir. We’ve understood that one, don’t bring back that reason. The one of fear

M: they have not exhausted the reason of fear. Because this reason of fear seems to be big, they give it a lot, it’s like saying knowing their status yet it has other reasons with in. what do you fear?

M: let’s listen to this gentleman

P: I test myself now, then I come to see you and I test again in two weeks and find out that things have gone bad, I can even burn you within the house.

M: you can burn her in the house

P: that’s the fear I talked about

P: that’s the fear that stops everyone

M: the fear that can even make you to make mistakes

P: yes the fear

P: fear

M: yes engineer

P: me what frightens me is the thoughts, and then secondly, I can test now when I’m negative and I get it at 10pm. I will have wasted my time

P: at 10pm

P: that disease is very worrying, it scares everyone.

M: let’s go to this gentleman, yes sir

P: it’s what got him from Kenya. This one

M: there are people that tell us, look here

M: let’s listen

M: we make the women pregnant, we men. When they go to the hospitals they are told to bring their husbands.

P: husbands don’t go

M: yes, do you know what the women do. They get boda-boda men and they take them there.

P: the reason that stops those men; the reason is one; men are not responsible about testing issues. They left those things to women that they should be the ones who test. When they see a car like that one parked there that you’ve come to test, aah, let me go the other side and get a bottle. Yet he doesn’t know his status. Another reason, for him he knows that if he goes and tests and learns that he is positive, that’s also going to become an issue for me.

P: it’s just pressure he will be dealing with

P: after knowing that that’s also an issue there, then he decides that testing or not testing me I won’t test. Let their cars be there.

P: Yiga don’t go very far, this one tests and he was told …

Phone rings and interrupts

M: let’s get this gentleman’s thoughts

P: I’m right here;

M: what fear gets to you that stops you; we’ve come, the health workers have spent a day there.

P: when they have come to test

M: yes, and freely

P: these things as you see, everyone grew up differently. Just like you see me, me I go and I get tested. Because you can’t tell me that going to test whether I’m positive can make me afraid, I won’t be the first. I have to be brave and they test him and get to know something.

P: we don’t see you test

P: don’t talk about me like that. Do you know where I get tested from? Me I get tested like every after three months. I get know something.

P: musawo, on that issue everyone has like ten points. Me I could burn you in the house and after I’ve known that I’m really sick, I also didn’t get it from a tree, I also start moving

M: what you are saying is that knowing ………….

P: because you could avoid HIV and get knocked by a car.

M: ok, the reason of HIV, many things kill people

P: you could avoid HIV and fall in the lake.

M: but still all deaths are different

P: well, if I have HIV, and I fall in a lake will they say that I died of HIV?

M: they are not the same

P: they will say that I died of HIV.

M: yes that’s it. Which means you can’t know that you have that thing and you just leave it.

P: and I’ve not been wanting young girls but I may start wanting them due to that

P: we hear people taking over dose tablet’s and they die, because of fear

M: but although that fear is there, as we are here, how many of us find the issue of going to hospitals as an easy one. Because most testing is done in the hospitals. How many of us find it easy to go to the hospitals?

P: when I’m going to the hospital to be tested?

M: yes, you as gentlemen

P: me

M: you find it very easy?

P: yes, I last tested…………….

P: me musawo, if I have money to ……….

M: where did you go? Nkozi?

P: if I have money to take me to the hospital, it’s the thing I want most in my life.

M: reaching the hospital?

P: reaching the hospital, normally I go to that side of rcc,

P: me I go to these ones you send to come in the field

M: MRC,

P: yes, if I’m around nkozi there, as for these comes that come, even if they come everyday

P: even if they come a number of items

P: even if they come daily

M: you don’t get tired of them

M: yes sir, what problems or how long do you take to go to the hospital

P: me, like four months

M: because you told us a while ago,

P: like 4,

M: eehh

P: 4 to 3 months

M: what hospital do you go to

P: me most times I know how to fall down and burst my head and I go to the hospital, before they saw me up, they first test me.

M: what hospital

P: in clinics

P: Basoga and up there at musawo Night’s place

P: you say that these ones that come here

M: but you cannot reach Nkozi?

P: Nkozi maybe if I want ……….

P: it’s the time he doesn’t have for Nkozi, but even those that normally come around, I have never missed; every time they come here.

M: if there happens to be a chance ………….

M: how about that gentleman ……..

P: me I’ve never been injected my entire life.

M: you fear injections

P: the ones for testing

P: whatever injection, I’ve never been injected on earth. 42 years

M: you fear them

P: I’ve never been injected and I’ve never gone to church, 42 years

M: have you ever tested

P: he has told you he has never been injected

M: he could be saying other injections

P: I’ve never been injected

M: ok let me ask, the question is directed to you. If there is a chance to test without injections, can you test?

It’s impossible, I told you whoever is to die on 20^th^ will die on 20^th^. It’s impossible; unless I’m tied up.

M: I’m talking about something that doesn’t include injections because we’ve known that you fear them

P: I’ve told you there is no chance

P: with that one you will sleep here

P: musawo, is there a chance of testing without injections

M: I asked the other gentleman, what do you think; if there is a chance. Because he is the only one I’ve found out that he is afraid of injections. Are there others that are afraid of injections?

P: me I don’t use it

P: not really

P: me even if you give it to me I can inject myself.

P: this one for testing, here or on the finger, that one you can inject but the one of the buttocks.

P: musawo, I’ve also spent years 40 years, but I’ve never been injected even if for malaria when I’m understanding.

P: waragi treats

P: but you find young people of 18 to 20 years when injections are all over their buttocks and yet they test.

P: I also went one time and the doctor told me, I had gone to the hospital and had some malaria. Then he told me that they were going to inject me. I told him no, I’m not injected. If you don’t have tabs

M: yes, how about you sir, how do you feel about our topic. Let’s listen to what he has to say

P: maybe, the topic w3e are talking about, what stops us from going for testing.

M: no, we want to know, which hospital do you go to for testing for HIV

P: the testing every after 3 months

P: that testing, if I’m here in kayabwe and I’m not in masaka, I normally go to a man called peter the other side; he’s the one that normally tests me every after 3 months

M: for you, you have your own doctor

P: who is private!

M: is he a family doctor

P: he isn’t a family doctor

M: does he test for free or you pay

P: I give there some money

M: let’s finalize with this gentleman

P: yes, I’ve arrived

P: chairman of barundi

P: my testing is in kayabwe, and it’s you that come to test me, and I know the truth but I don’t travel to go to Nkozi, because I don’t have money to take me there.

M: they are bringing eats, let’s get settled. Now what I’m requesting, government or the district, what do you want it to put in place to increase testing. What advice do you give us so that we can carry it forward? The thing is here. To increase testing of the HIV virus; I will start from here.

P: the advice I give you ……

M: first wait, let’s start from here then we shall come. Let him bring, then the other will bring and the other will bring. Where do you want us to rectify. What do you want us to do to see that the men; because if you say that we change here it’s the women that will still come. Let me give you an example; this hospital of Mpigi, and all these other hospitals, just that some things are not talked about, there’s an NGO that used to put money; when whoever comes with a man, they give her ten thousand and they also give the man ten thousand. Those that knew were taking people.

P: me I even got twenty thousand at some time

M: aahh, ok sir what advice do you give us, to continue attracting us men.

P: to attract us men to go with our wives, if there was money, maybe people didn’t just know.

M: what advice do you give us? What did you say?

P: the other issue we have, if I’m to test for free, I have to board and go where? Nkozi, if there was a hospital around here when it’s also free like Nkozi, we would be helped.

M: how about the people that come here.

P: those that come here!

M: so, you want them to build another level here.

P: for free, well you see.

M: thank you; sir, what do you ask of the government and other agencies in health

P: agencies in health, what I’m asking of them and the government, let them just put in more effort and send those teams that keep coming to the village.

M: yes sir

P: the advice I give is that they move house to house, in the villages, when they are two health workers on a village. Two health workers, a man and a woman; when they have a gift to give them to keep them around

M: will you be around you

All laugh

P: he will be around,

P: but when it’s house to house.

M: house to house; will you manage to be around?

P: yes

M: yes sir

P: me what I request of the government and what it should do; is to send us these health workers to come to test us and they find us where we are in the villages we are for free. And they shouldn’t get even one hundred shillings from you. But what they should do; they should put a branch or pharmacy when people go and test, and they are given maybe days like Friday or Wednesday for testing and it’s free and it will help those that don’t move a lot. That money will help those that move a lot in villages going to test for free and just be there and just communicating that in kayabwe, we’ve tested these people, we’ve got these patients.

P: me I support this man’s idea

M: yes sir, let’s go ahead. Give us your thought; what do you ask of the government?

P: me as me, I ask the government to increase on the number of health workers who come to villages. In towns like this one they come and we converge in one place and they test us. And nothing like asking for money.

M: so you want health workers to come from nkozi in large numbers.

P: or mpigi

M: but you may wonder, when they come most of us don’t come

P: no, we …….

P: if word passes about that salt.

P: they come and they park the car like there, most people don’t know that there is testing; many people live in the village

P: musawo me I’m asking, when I went to masaka at RC. What strong agency is that?

P: Mulago;

P: not Mulago, RCC,

M: RCC, when I went, I had my person that took me there.

M: a man or woman?

P: she was a woman. And at the reception I was asked, where are you coming from and I said Namasuba Zana. They gave me twenty thousand shillings; ten thousand to bring me and ten thousand to take me back and I also got a good lunch. So that encouraged me to keep going for testing.

P: exactly that

P exactly that

M: yes sir, we are here. Your message to the health service providers, that are concerned with health and testing and attracting men to come and test for HIV.

P: it would have been that the health workers are many, that try come. Now there is coming and you come 3, like how you found me when I’m in a hurry going; then you start wondering lining up and when they will get to you yet I want to go and get food for my children. I want to spray, and time is going then I see that I can’t manage.

P: and you lose that chance.

M: but if it’s that the health workers are around in plenty. When you arrive, they work on you, get your results and tell you that you go. And they also get a way of lying around.

P: not lying, telling you

P: lying, are you lying to a young child

P: when a person hears about eating.

P: that haha

M: well, you’ve all talked on the side of the government sending you message; but you have not told us the reasons that when we also see that the health workers are many we shall manage to gather ourselves or telling each other. Sir let’s go to another reason.

P: sir me what I see, it would have been like this. They should be walking, the health workers, house to house.

M: like what the other gentleman said

P: yes what he said

M: yes because he said he won’t run

P: if it’s house to house, if you finish testing someone and they are positive and you even have some medicine you can give them then, and some money, people will endeavor to come

P: or sugar

P: or sugar

P: but when it’s house to house.

P: now for you you are in town and the others don’t know

P: they leave you something and some medicine if they find you when you are what: positive. You tell them start from here.

M: start from there. Thank you sir; yes sir

P: me I would have said that the health workers should have been more; but still let’s say if they are to come on a day maybe today, they should have announced like yesterday on the loud speaker because there are people from far, when you can’t get the communication like how you’ve come here.

M: but if we announce again wont men run

P: wont they hide

M: surely they will run

M: the man will run, and we could even get responsibilities, Yes sir

P: me the thing I see me kiseka I’m one person; if you give a day and be like on this day we shall be at kayabwe, surely if someone has pain, they can manage to come and test, they get tested and know their status. But you if you wait that the one feeling could again they show you to the fire aahh. I don’t know very well. Whoever can manage and go, sekagale like the other one when is also around; aanhh that’s my suggestion.

M: how about you sir the last one here

P: Me as me, I suggest like this

P: haaa, now that one, isn’t he the one with over 150 children

All laugh

P: don’t start on me, don’t start on me

P: me as me, if I’m, now like the health workers, it’s paramount that they increase in number. Then they go through people, and they get to know the truth. Them as health workers to know the truth and the people as well to know the truth. Because me I won’t be afraid of testing. That’s it

M: well well. Now, there’s something this gentleman talked about. That if they increase the number of health workers and something like that; if there is a chance, when you yourself, you can test yourselM: wherever you are, so that you know your status. How would that be? Now like it is that we first go to the hospital, we always have many reasons like this, when the health workers come to your place, you still have reasons why you haven’t come. That they have delayed or they came but they are few. Some want to be approached in their homes where they are. I think you can see those reasons; which looks like it’s true that these health workers can not make everyone happy or reach everyone. If there is a chance that you yourself you test yourself, how would that be?

P: musawo?

P: musawo me I have

M: you know for yourself

P: it will bring murder in the home

P: the main point is like this, that the men are hard about testing, because if you are really concerned, there is no reason for you not to go there when the health workers have come to town here. If you are in town, something that concerns your life, if you are not afraid, what reason will you have not to go and test for free?

M: you gave the reasons, you gave them. And surely that’s how it is

P: yes, what reason really

P: people are just hard

P: they are afraid at heart

P: they are afraid.

P: that thing is like, you could be facing challenges, but if you find out that this person is the one bewitching you, do you go and cut them? Do you go and cut them?

P: it could bring murder in a home

P: not really

M: but first answer what I asked. Give me your thoughts on that topic; if there is a chance when you yourself you test yourself.

P: yes, now I’m I going to buy that thing and I test myself or not

M: let’s look at the thing is around, they have handed it to you; let’s first come back from where you get it, it’s around and you have a chance to test, how would that be.

P: that one I see it causing differences in a home. Just like this gentleman said that if he has the HIV, he won’t tell here.

P: yet you test yourself?

P: now the woman; no, me if I’ve tested then the woman has not tested. When you come home, the woman gives it to you that you get and test yourself, now these things; if she finds you with Nabuma and you are conversing, if she has always wanted to leave you, that’s when she goes and brings it that you get and test yourself.

M: so for you you see that it’s completely impossible for you to test?

P: me for myself at home,

P: that’s chaos at home

M: you what do you think?

P: the home will have ended

P: the home will have ended

M: what do you say about this thing, if there is a chance.

P: me I see, if there is a converging place, ok maybe like a hospital.

M: no. we are looking at if there is a chance

P: when it’s you who test yourself

M: when you test your own self. Because we’ve seen all these reasons, if it’s a hospital, all the reasons that have been given we’ve heard them, those that don’t go there are here, if they come to your village, we know what happens, now there is a chance, you test your own self.

P: now like me on that issue, that’s easy on my side to test. If they give it to me like this that it’s there test yourself. That’s very for me.

M: you think it will be easy?

P: it will be easy for me

M: you don’t see any problem with it?

P: it’s easy for him because he is single

P: this one picks from the road

All talk and laugh

M: silence, silence

P: me what would have been easy for me, it’s being like you the health worker you have come, then you test me, and I know my status

M: we left that one

P: now when I know, when I know, I ask you that what I should do. But here if I test me as myself, and I find out that I’m positive, I leave it at that. Then I go and ask my waragi and I take.

M: on that same reason, because most of you hear except for one person who said that he can’t test , but others we have tested before, that’s what I think

All agree

P: apart from Mr. Yiga

M: now, we’ve tested before, but we’ve been having reasons that stop us from testing all the time like we should be doing, that’s where I based to tell you that, even if you’ve tested before, if there is a chance now, there is a system that you yourself, what do you think about it; even though back then you’ve been testing testing, wherever you find them you test, now there is this chance. You test yourself, we’ve looked at it, remember we are still on the same disease.

P: in that state, I won’t be able to give myself any help.

P: musawo me I have a question, me I test myself?

M: you yourself

P: when you’ve also given the wife her own thing or

M: no, you just you; we’ve started with us

M: you, just like we are here

M: there is a chance

P: I won’t get counseling

P: now, if you give it to me to test myself, after knowing that I’m sick or not, I tell the wife or I don’t tell her?

P: definitely you don’t tell her, the other woman

M: you know for yourself, you know then you decide.

P: even you are tired of the wife, you’re looking for a way of making her leave.

It’s like how we started earlier before he talked. That you’ve been found sick, the wife has not yet found out, now you have tested yourself. They have taught you, and counseled you to give you the other think; you as you, she is asking, she wants to know, what situation you maybe in after you’ve tested and found out your status; whether you are sick or you are not sick. It’s what she’s trying to make us understand that you on your own you’ve tested yourself, there is no other person coming to test you.

P: musawo

M: you’ve tested yourself,

M: the truth is like this, in addition to this gentleman’s; even if the health workers are around, they walk, in hospitals they be there; the truth is that they don’t reach everyone. That’s why I asked, if there is that chance, that you a person who maybe be in need of this, you have a chance to test yourself, how would that be? The other one gave us his thought. Even the other one gave us his, it was burning, and there’s another one he was bringing.

P: I was telling this man that it brings chaos in a home, I’ve tested at this time, I’m negative, I gave a wife at home, what do I be thinking when she hasn’t tested.

P: you even know that she’s sick but you’ve always looked for a chance of her leaving you. You’ve tested and you are negative

M: you test and know for yourself

P: sometimes I could be even knowing that she’s sick but when I tolerate her because I know I haven’t tested yet

Some laugh

P: now I go back and tell her that cook food or to find the route that brought her

Laughter continues

M: others this thing of testing yourself what do you think about it.

M: you man you’ve made me laugh, sir they are asking you. What do you say about it?

M: yes, there is a chance now, you are going to test yourself as you

P: are you ok?

P: I’m ok

P: me for me, when I get up and go to the hospital for testing as me; I do that, every after 3 months. For me my wife is in Masaka. If I find that she got weak and became sick, when I’m not, what do I do at that point; I chase her and she leaves my house or what do I do in that situation?

M: because we are now looking at the chance to test yourself

P: musawo, I have a question, do I have the chance to test myself

P: when I test and find that I’m negative

M: the chance is there

M: the chance is there

P: now, how do I know that I’m sick

M: the chance is there and that’s why I’m asking you like that, if there is a chance when you yourself you can test. And know; because today, because of those problems that we face, government has prepared a new method where we the people test ourselves

P: we test ourselves; that’s the best

M: that’s why I asked that if there is a chance

P: it remains your secret, you know it within you.

P: it’s in secrecy

M: you yourself, you yourself

P: they will give me that one, I go to my room, then I do what,

M: give them papers, give them papers

P: it would have been good for government to give us that thing and you test yourself, but if like two months pass and you see that you are sick, then after like four or five months and you test again there there’s, just that I don’t know if they give you soothing medicine or not

M: you hold two people there. You hold two two people because there is explaining to each other and pointing fingers. It would have been good if they hold two people because there is pointing fingers, there’s someone who was here and he has run. You with him, him with him, this one also has someone he will see with. Yes, on that same issue of testing, I’ve told you that government has a new method it’s preparing; and that new method, it’s the one we are discussing about on that paper we’ve given you, the other gentleman, when I asked him first, that if there is a chance and you can test without being pricked, this method is one of those methods. You can get tested without being pricked. But another thing it has is that you can test yourself. Now I’m going to let this gentleman to first take us through this method and we see.

M: let’s get closer, have you seen the paper?

P: yes we’ve seen it,

M: are you holding it as it should be?

P: yes

M: the method of testing, it’s this one for individuals. The method of testing yourself when you are using what? Saliva. Are you seeing number one? Have you seen it?

P: yes,

M: eeh, there’s a sachet or the thing

Off topic talk, chasing away a woman who tried to join the group

M: that sachet, we shall give it to you, when you hold it down; there is a swollen thing, even up there is one. Let’s go to number two

P’ yes

M: are you seeing number two, he is holding a corner of that sachet, and is tearing, he is tearing there to remove what? A bottle or tube, a small one, and you can see it here in number two where we are exactly. When he removes it out he will see where the cover is

P: in number three

M: in number three, to open what? The bottle. And that bottle if you are looking at it keenly, here you may not see the water in side it but it has its water, which was made in there. In number four, he is done opening it, he is seeing what’s in the bottle, after opening it, you can see what’s inside, but when you haven’t tilted it. You have to hold it just like you are seeing how he is holding it. We’ve gone to the other part of number four; now after he’s done removing it here, he comes to the upper part, do you remember how I told you, the sachet has two parts. The upper part, he still tears at the corner, in that corner, he removes something that looks like a toothbrush. Others say it’s like a spoon but if you look at it, it’s like a toothbrush, but in tearing, you won’t just tear, it has a head, and where you hold. Just like you will hold.

P: it has where you hold, don’t you see he is holding it here

M: anhhhhaaa. Now number five, do you see number five?

All: yes

M: well, now he has removed the tooth brush, do you see that toothbrush, it’s for handling very carefully; if you see it, it has a head, and the side you touch, let me start with the head. If you hold like this that toothbrush, this part you see that’s pointing down like that, that’s the head, like you can see here, that’s the head. The head has a soft part.

Off topic charts, someone complaining about a pick axe

M: let’s go ahead, now that head; it has a soft part like cotton. Have you seen it?

All: yes

M: well, that head, do you see where you are holding like this, there are two lines.

All: yes

M: those lines are to handle very carefully, and you touch down here, such that you don’t spoil other parts. We are going ahead. You will hold that toothbrush like this; the other part I told you about, the lower part

P: yes

M: the part with a soft feeling; you hold it like this and you put it above the gum. Do you know where the gum is?

Fs; anhhhaaa,

M: then you turn. The clock, the clock moves heading where.

P: the clock moves like this

P: like this

M: where does yours head?

P: like this

M: laughs, I saw like it turns this like this

M: the clock turns like this

P: yes

M: but this time round, where the clock doesn’t go is where you turn that thing. Now for me I’m seated like this, I put on the gum this side and I move slowly until. Do you listen?

P: yes

M: you see how I moved

P: yes

M: I get it from here, up to this side, but inside the gum, then I get it from up and I bring it down but this time round, you move it like how a clock does what? How it moves. You put it under the gum and move it up to where it stops, do you hear me?

P: yes

M: that’s number six, if you look at number six, up, he is above the gum, down, he is below the gum. Do you see that clearly?

All: yes

M: then you go ahead and remove it, that thing of ours; then you put it back inside the bottle that you opened as you see in number seven. The gentleman is trying to put it back. Number seven he is done putting back part of it. In number eight, do you see it?

All: yes

M: now when you’re done putting it back, you finish twenty minutes. But let me first tell you again what I forgot. Before you put it above the gum, you make sure that you have spent like 30 or 20 minutes after eating or brushing. Reason being that after youre done eating and you say that you are going to test, the other food that sticks on the teeth is the one that will get onto our thing and our results will get lost. Or even this side down. You have to test when minutes have passed how many?

P: thirty

M: or twenty; either after brushing or eating or even taking this soda of ours. Let’s go back to the 20 minutes, let’s go back to number 8, I wonder if people this side are seeing me.

M: they are seeing

M: let’s turn over

M: we’ve turned over, to this side, after the 20 minutes, we are now going to read the results, what has come out of the testing.

M: yes, do you see where there is C and T, two lines, or boxes

M: we are up here, you see this starting part.

P: C and T

M: or call them results readers. This thing you are seeing, it’s the one we put inside the tube/ bottle. It’s showing how many? Two

P: two

M: this first one and the second one; you will find that there’s one that has passed through C and one through T.

M: because this is a photocopy, we used a pen to make them more clear, but the original is in colour.

P: they are visible!

M: in colour

M: it can bring red colour, with two line when it has passed through C and T but in red or green or other colour but they are two, that means you have HIV,

M: even if they are faded

M: even if they are faded how; because here, we saw two lines but they were faded; just because we came with a pen and marked it. You see they are two

P: C and T

M: C and T, do you understand it my friends

All: yes,

M: they gave you the other thing and you tested yourself alone; you’re in the bedroom alone, you didn’t bring even the wife. Let’s go to the second box. The second box wills show you that it has brought one line on C, do you see C? T is empty,

P: C has a line in it

M: it has a line in it, it’s on C, remember, C means control; what’s control in Luganda

P: “kwegendeleza”

M: we make some English words luganda; control there. C

P: control is kwegendeleza?

M: yes that’s one, how about another one, sir, control in luganda is what

P: control has many meanings

M: yes but I want in luganda

P: “mwekomeko awo”

M: mwekomeko awo! Then T is test, you will ask me that T is for what. You see it, if you find T, with one line on T, you will be negative. If we go to the last boxes here, these three.

P: the two

M: yes the two, just that this thing is not showing clearly, if we photocopy we get things that don’t show clearly.

P: it’s for wrong results

M: this one is for wrong results. How? It’s the other food I told you; you’ve just finished soda because you’ve got it; in packaging it was packaged wrongly; in transporting it from mpigi to kayabwe, it fell down and it got some damage; you won’t get results like these ones above. And other things that make results fail to come out well.

M: like one of those reasons he has given it to us; the other reason, he talked about it, like here we all took soda but who checked the expiry?

All laugh

M: now this thing, I’ve just used and example, because it happens often; and it’s the same thing with condoms. We have to check the expiry dates of these things. You see it? Now even a person who didn’t check the expiry if they use this thing when it’s expired, even if it’s a condom and you use it when it’s expired, it won’t give you protection you hope it will give you, just like this one won’t give you results. The other reason he explained it about food. The other reason where it would have been, it would have been here in opening. You see this sachet that he showed us; for you, you see as if it’s faded but it has a marking that shows the behind of the tooth brush it already shows you; this side that has where this big thing is facing; it’s where you tear, but for you if you tear this side with this part for passing on the gum; you tear this side, and you remove when the part for passing on the gum is the one you are holding, you held it first.

M: let me repeat for you

P: musawo

M: you won’t get results

M: let me repeat for you something small.

P: let me say on this thing, where I haven’t understood properly

M: we are coming there wait a bit

P: here here

M: yes

P: I see this side is big and this one is small.

Fs; anhaaaa.

M: now I was showing you the way they open, here in opening, you see here in opening

M: you show the other man

P: you will forgive me, I wasn’t around

M: first wait, first wait; we shall get back to you shortly. We’ve left you a long way. You see this side where they have showed a marking here? We opened from here. Do you see this side that they tear, if you see the marking on this thing? It shows where this big thing is it’s where you have to tear then you remove, because where there is this thing for passing on the gum, you don’t have to touch it. If you open the wrong side and you touch where you are supposed to pass on the mouth; you will have made it dirty.

P: that one will be dead

M: another reason is when you are opening this bottle, if you open very well from number one up to number four then you come and pour some of this one

P: me first give me and I go and test and I see

M: first wait, as you open, you pour some of this and it remains very little then you may get this, when it’s not enough. Another reason you may find yourself with results like this when they are wrong; if you open very well and reach here, but you don’t be patient to remove this and use it, then you decide to spit in the bottle and then put this one; what do you think you will see?

P: wrong things

M: wrong things, and another thing that may happen is like this

P: it’s just that our government is not well.

M: my friend first listen

M: first listen

P: that thing, me I enjoy very much, they call me to see, when you are really there opening it

M: not really,

M: we are few, next time we shall come when we are many

P and they will be available

M: but first listen

M: if you come and open this thing, where mistakes may arise, I’m showing you; you’ve opened very well, the one about spitting in you’ve heard. You won’t get the truth. You heard the one about touching it, or if you pour the liquid. For you you came late so be patient, you can’t ask questions, if you pour it and a little remains then you won’t get results. Another one is like this, if you open it well from number one to number four, but you don’t pass on the teeth first of all, and you dip this inside the bottle, then you remember that you were to first pass it on the gum, remember you first dipped it, then you removed it and passed it on your teeth then you put it back, you will be here. What does it mean? That we have to follow these instructions. These pictures because they are the ones showing you; number one do this, number two do this without short cuts, for us to be able to get results. Because remember when we had just introduced this thing, government is preparing to start a new method where us people in need of knowing our status, we can do it for ourselves. That’s why you see that today, we’ve not brought those things because it’s new; if something is coming up, it’s good to first show someone how it works and they first look at bit before you bring the real one because remember, all these instructions won’t be followed and he will make mistakes; and remember where we started from, the thing we are talking about, everyone shakes their head about it, it’s a big thing and that’s why we first brought this thing to explain to you. Now, let’s share our thoughts, Mr. Mukasa it’s here I’ve returned it.

M: well, let me give you an example; they brought for us male condoms, yesterday I was in a mining ground in Muduuma and one gentleman told me that musawo we have some problems. You date a girl, when she’s still in school and she lives with her parents, then they send her to a shop and she gives me five minutes to use the condom. Do you know what he does; the girl tells him that I’ve been sent to the shop, you have 4 minutes now on the five I first told you. The he pulls out the condom, remember it’s at night and you are in a hurry; he tries this corner, it refuses to tear, he tries this one, it refuses to tear, he come to the other one, it refuses to tear and the girl is waiting saying daddy, mummy

P: he is left with two

M: then he gets it and puts on the mouth

P: and he bites it

M: you don’t k now what you’ve bitten whether you took part of the inside one as well. When it comes to wearing, he wears it upside down and then he changes it until it gets on correctly

P: aren’t they these ones they made small

M: I was waiting for the small issue to come out; laughs

M: they over say it

P: that what

M: that they are small, the condoms

P: the condoms are now small

M: the results my friend

P: it can’t be small

M: it can’t be how

P: yet you can pour a whole 20 litter jerrycans and it fits in there; I can’t allow

P: these days there are small ones

M: you see that thing, you shouldn’t under mind it. It’s very serious. Its peoples thoughts and they have been giving them and we are going to take back because remember this thing people that use it have something to say. They have a right to talk about it.

P: they are small, they even bring us blisters

M: ok, answer, the results that musawo was telling us about; a woman can come up and complain and you tell her didn’t you see me wear the condom? Or even a woman. You see how a conflict starts from there. The second one, we talked about saliva, someone can say; “what means even kisses can do what”

P: this is the guy that talked about it

M: eeehh, the elder that talked about it; No, this thing was made when it has phones that it can see in that saliva; It on its own; because you might come and say you’ve come to test for HIV, and you instead test for malaria. And you say musawo, let me try to test for something else. No, it only sees the phones of that virus that are in that saliva. That’s why we say that first clean yourself for like 20 minutes so that you also settle because if I brush in the morning, you can tell that I’m from brushing, but after 20 or 30 minutes I will have come back to normal; for it also it takes those phones that will be in the saliva; the other thing, how did we call it? The virus, it has many things and it’s what this thing will test whether you are sick or not. My friend who has come here, you will teach whoever hasn’t come. But what I request you, they will ask you where you got those papers, then they will read about it and you will be like we were in an education session there. That’s the thing we want you to teach your other colleagues. So that when this method comes, it’s easy for others. I was talking to musawo and I asked her, how much, how expensive is it? It’s really expensive, because even if I come here when we have some that we have brought, I will still look for you. I will tell the VHT that the gentlemen you gathered for me last time are the ones you should give. Then you will go and test yourselves; now if you test wrongly, you will have wasted money; if you don’t test, you will have wasted money; that’s why I’m requesting you to see that you use it in the way we trained you to see that we money is not wasted and we also benefit.

P: musawo, for me the person I’m sharing with stays very far.

M: I will give you this one I’m using; another reason, we were told some fear injections. But this method has no where you will bleed from. My elder you fear injections at 42 years

P: that one will be easy for him

M: actually he would have told us, do you see any good in this method

M: that’s where we should head

M: do you see any good in it?

M: let’s hurry up

M: then we shall conclude

P: I see a good thing, a person testing, if they fear injections, he won’t get the pain

M: how about you, who was saying no no no

P: I also won’t feel pain

P: he’s changed

P: he’s changed

P: we have you

M: you see the situation you came in and now you’ve changed. Yes sir, what advantage do you see in this method we’ve taken you through.

P: that method will bring people who are down there,

P: even those down there

P: down there where that hill is, where it ends

M: how is it called

P: they call it mulufunda that side down

P: bendegele that side down

P: they will manage to get this thing and test without the need to get three thousand shillings to go to Nkozi, or here. Do you here?

M: thank you

P: musawo, will you have transport for these things

P: musawo we talked about expiry dates, this thing, does it work once or you can reuse it

M: anhaaa, exactly, I had forgotten

M: you’ve raised a big concern; I was need to hearing such a question. What do you think

M: what do you think as you

M: let’s go back to blood, the one for blood works for how many times

P: the one for blood?

M: yes

P: it works once

M: it works once

P: me I see, after pricking me like this, they put blood on that thing, then what is left they throw in the trash bin

M: yes, before musawo asks something else; what do we do with the condoms?

P: it’s once

M: how many times do we use the condoms?

P: it’s one time

P: the condoms goes in for one route and it comes out and goes the other side.

P: and it goes the other side

M: I think where we are we are in the answer, playing in it

M: it’s used by one person and just one round

M: one round, yes what other advantages do you think may come with this method. Because all these come to try and make life easy for us, but we need to tell them the good in it. If we don’t bring out the good we think then it won’t happen. We have to bring out the good and understand it, and after we are done with the good, then we shall bring out the bad we also see in this method.

M: let’s start with the good. Remember the other gentleman told us that he will be around to test, he won’t run, and he told us

M: what good do you see

P: the good I see in testing

M: individual testing

P: the good I see in individual testing, is knowing as you then you be careful on what you are going to do. So that you don’t transmit to others

M: yes, how about you, what good do you see in this thing?

P: as Mr. Richard Birigwa

P: the good in it, just like the point the other gentleman raised, it’s the one I’m re-echoing, you test when you are alone and you get to know your status when you are alone.

P: when you are alone

M: laughs, ok, how about you, what good do you see in this thing.

P: I want to ask a question to my friends, all of us here. Will this method reduce HIV in Uganda or not?

M: let me add for you this one; will it tempt men who fear the injections and going to the hospitals? Add for us that one

P: will it tempt men who fear going to hospitals to test to go and test?

P: it won’t tempt them;

P: it will be easy for them

P: it will tempt some that are able because it’s for money. But if someone doesn’t have the money they have attached to it, he can’t afford it, he only looks for that days food; then it won’t be easy for him.

P: how about reducing HIV,

P: musawo, this thing

P: you are the knowledgeable one

M: first keep quiet, yes

P: this thing, how much will it cost

M: is that what you also wanted to ask?

P: I wanted to ask that this thing if I need it; in a government hospital will it be for free or not.

M: that thing is going to be like condoms as you these days. How do you see them? Who has ever bought it with his money?

P: me

M: and who has ever got one for free

P: the free ones are fake

P: the free ones are at Nkozi

M: first wait, it’s like testing these days, you’ve ever gone and paid some money and you’ve ever gone and tested for free, but all of them

P: free ones are fake

M: no no, it’s truth

M: it’s true

P: sir, free things are not fake

P: they are all true

M: first listen

M: it’s just that the voices are going the other side, they are not coming down; now another thing before we leave, it’s very important because we’ve looked at this thing. Before I ask the last question because we are also about to let you go; but we shall ask you as we leave, everyone what they have understood. But before we go to that. What disadvantage do you see in this thing of everyone testing themselves.

P: there is no bad thing

M: because we’ve brought out the good

P: I see they are all good

M: we’ve brought out the good but it’s important that we think past the good alone and look on the other side. It’s only this gentleman that brought out a complaint and said, ok even though but for him he talked about a complaint. Others what complaints do you think see that maybe in this thing when it’s you who tests.

P: there is no complaint

P: me the bad I see in it; if I be when I know nothing about it, I don’t have any counseling at all, but I’ve got this knowledge and I test; I’m at my place, me as a single person, I always get high blood pressure, yet if I’m there with a health worker, I leave when the pulse isn’t so high.

Some laugh

M: now on that very bit, it’s where I will ask from; even though this method is for individuals. Where would you want it to be stationed? That if it’s put there, it will be easy to be picked and still I predict that wherever you get it from, the person there will be able to give you some few words before you go to use it. Where do you think, now like in this area of yours, when you won’t find hard time to get it.

P: the chairman’s place

M: because remember hospitals have their own challenges

P: chairman’s place

M: no let’s get suggestions from everyone. Sir where do you want the method to be stationed?

P: you first be asking the other side

P: on the village

M: where? Do you want us to just throw?

P: at the local council

P: no

M: how many times do you go the local council

M: sir where do you want this method to be stationed

P: in Soweto

P: this method me I’m asking, let them look for a clinic close by, and they put that method and people will know that when you pass by this place, it’s where you will find what, the individual method

M: how about you sir

P: me the way I’m thinking

M: you’ve really given us thoughts

P: not to make things hard, for us here we are very close to the big government hospital.

M: which one

P: the one in Nkozi

P: no

P: we again board and go?

P: what if you don’t have that 3 thousand

P: there is not far, if it’s really there that you are going to find that thing. Because you can’t put here such a thing

M: that’s his idea, yes sir, your thoughts

P: my thoughts, here in kayabwe there are many clinics

M: you’re for clinics like this one

P: yes, if they announce and be like this method is at this clinic, we shall come there

M: ok thank you; sir this method where do you want us to put it where you can find; I mean you get your time

P: but time has gone

M: yes time has gone, let’s conclude

P: I would have thought; just like this lady brought you and showed you to us; they show us that person that they it’s where you are going to find this method. When he/she is a fellow village member but they can note down that I know this one, he has come and got, I know this one, he has also come and got.

M: that’s where we conclude from

P: I won’t differ much from the other two gentlemen

M: which ones

P: the other two who said we get a clinic or pharmacy and they tell us that you come here and we know when to come.

M: yes sir.

P: I won’t also differ much also, if we get a clinic, we can all come and be there. That’s my thought

M: well, now, these are the last thoughts; I hope we have discussed it all because my friends, those that haven’t got this chance are going to base on this, we’ve all known the reason for this virus why we test it in saliva?

All: yes

M: how many of us know that the virus can spread through saliva. This one hasn’t gotten yet he hasn’t got please give me one. The other one. How many of us still think that saliva can spread the virus? Any one still knowing that saliva can spread the virus

P: no

P: I learned it from here

M: that it doesn’t do what?

P: that it doesn’t spread the virus.

M: it’s very important. But why do we use it for testing? Because we’ve known the other thing

P: we’ve understood that the other thing we learned about, it doesn’t see the virus in saliva.

M: we, now I’m going to ask before I turn off, before we go to those papers; Is there anyone still with a question about the things we’ve discussed today? That we shouldn’t go with

P: me mummy

M: yes, let’s listen up,

P: Me what I’m asking, as you’ve helped us and brought this health education,

M: yes the last question

P: no

M: the last question, yes

P: my question is that

M: please let’s listen to this question then we start writing

P: I’m asking it to you as health workers; are you going to get another day and you come and tell us and teach us the other thing live as we see it as you show us the example as it should be. Because you cannot show us on this paper and we really understand.

M: exactly, that will be done
