# Supplementary material for: Prevalence, associated factors and perspectives of HIV testing among men in Uganda
Source: PLoS One. 2020 Aug 7;15(8):e0237402. doi: 10.1371/journal.pone.0237402 (PMC7413494; doi:10.1371/journal.pone.0237402)
Supplement: S1 File — (ZIP) [file pone.0237402.s002.zip › manuscript data/FGD Kayabwe 3 Eng.docx]

M: We are here to share ideas. For those that weren’t around the last time, we will remind you. But we are only here to share ideas on the topic we are about to share. Are we together? We are going to share ideas about testing for HIV/AIDS. That’s the reason we are here today. We are here as male youth. However, whoever is below the age of 15 years should not be with us here.

R: There is none.

M: That is good. Now we are gathered as youth. We should be open in our discussion and no one should give an excuse of being shy. Most of the people here are your friends and you know them. If someone says they slept with 16 girls last week, there isn’t anything you will do about. So let’s be as open as possible. We are here to talk about testing for HIV/AIDS in individuals but male youth especially. I would love us to share knowledge about the different methods. This gentleman here is going to moderate us. Let’s ask a few questions and we can go ahead. The topic of discussion today are the different methods of testing for HIV. I want us to talk about what challenges we have faced using these methods, what you think about them.

R: Let us first talk about why we do not want to test. When you go to test, you find that the professionals that help have their personal problems. They will tell you to wait. And yet you left work for testing. We don’t have that time to waste lining upon to test. They will delay you and yet the hospital is also far from home.

R: When you test, the profession will give a contact. You will realize that it is not even their contact. And the injection is painful. I got one for syphilis but it was very painful. The other thing is that when you test and find out you are positive, you worry so much. The medicine is very bitter and yet they are to be taken daily.

R: We admire the rich who get the drugs monthly or annually because daily medication is like you’ve committed murder. There is even a myth that when you start on medicine you start having nightmare about funerals.

M: Let’s listen to one another members.

R: The ARV tablets are sour and smelly. We are poor. We cannot afford sugar to sweeten them up. The tablets are like for pigs.

M: Let’s go on.

R: The reason I have failed to go for testing is that. When you go to the doctor. They ask you a question about what you would do if you found out that you are HIV+. I will tell the doctor that I won’t take the medicine. I would rather commit suicide because am still young and will not be able to enjoy life. Sometimes the doctor will not tell you the truth after knowing your reactions. Sometimes those people travelling outside the country, get tested and if found positive, they won’t be able to travel. Those are some of the reasons I don’t want to get tested.

M: what are your reasons for not testing? The rest.

R: I have a friend of mine who I just escorted to go for testing at Uganda cares Lukaya. After that, we came to Nkozi to confirm the results because he was told he is positive at first. When he came here, he was told he is negative. That’s how I ended up not testing myself. I don’t want to test and they start making errors on my life.

M: Can I ask you a question? Have you ever asked yourself why it turned out like that?

R: I asked him instead of the doctors why he was positive. He said he didn’t know why. However, when we came to Nkozi, he was told he is negative. I decided not to test to start pressuring myself.

M: Others, what are your thoughts about that?

R: According to me, the biggest reasons for not testing is the injection.

M: The injection?

R: Yeah. There is a way to disorganizes you and takes your peace away. Even when you are told that you are HIV+. Starting on drugs is difficult. Then you are told that nonsense of nightmares about funerals. They also say that when you miss taking the drugs for a day, you will die. That’s what stops me from testing.

M: Do you have what to add on that?

R: The drugs are very sour. They are even nicknamed nine millimeters. They bring so much discomfort when taking them. It’s very hard when you don’t have food. Also, taking ARVs is at specific time and yet sometimes you can travel or be at work. That means you have to always come back home to take medicine. When your woman finds out, she will start denying you sex. But I have a question? Do you die if you miss out on taking tablets for a day?

M: We are going to answer all questions but for now let’s share some more ideas.

R: Can’t you make for us injections that are administered once a year even for the poor. And in addition to what he said, you might get a job that is far from home. You can’t always move with the tablets.

M: To able to share our ideas well. We shall talk one at a time. You’ve been sharing your ideas about the reasons why youths don’t test for HIV. So now what do you think can be done to change that?

R: Let them get another method to test. In line with that, let the poor also be considered by making tablets that can be taken once a year. The youth are the biggest part of the government as we also pay taxes.

M: Yes, sir, we are sharing ideas, is there anything you can add?

R: Yes, they should find another way of testing for HIV. This is because the youth are afraid of injections. In addition, there should be another way of managing HIV. Tablets are not effective as youth get tired. They can stop taking the medicine thinking they are okay.

M: Why do you think someone can withdraw from taking tablets.

R: They are sour and bring nausea. Also, people don’t want to leave work to swallow medicine. You can’t allow the woman to see the drugs. They should also introduce weekly-administered tablets. Sometimes we use sex workers who can check you can find the drugs. They will go around telling everyone that you are HIV+, which is bad for your reputation. They also say you have to take the ARVs with Septrine (Cotrimoxazole) but it usually on sale, now for someone who is poor and can’t afford the drugs. Won’t they die? What happens if you don’t take the Septrine?

M: Thanks for asking that question but for now let’s continue sharing ideas on why youth don’t take medicine.

R: I also have a question. Am told when you test the virus reproduces at a very high rate. So who dies first between one that tests and one who is not aware of their status?

M: Yes. We will answer all questions. But for now let’s first exhaust the topic of discussion. Why do men fail to test and yet women go and test?

R: There is a law that allows a woman goes to the hospital. They are tested for HIV. This helps to stop mother to child transmission. And they can also be influenced by their peers. Others test to find out about their status and go ahead to infect others.

M: Let’s continue sharing our ideas about the reasons why women test for HIV more than men.

R: I think that men are busy making money. We were happy with the in-house method of testing. Most men use their time to work and get the money. I think they should find at their work places.

R: There is nothing much I can say right now. Men are just busy working. Women test because they don’t trust their husbands, they think they can get infected by their husbands. Moreover, for the men when their wives test and find they are negative, they instead find no need to test as they think they are also negative. I have question. I want to know why we are lied to. How can my wife be negative and am positive when we have sex all the time?

M: We will answer you. We have time for questions.

R: We are told to use condoms and yet we hear condoms have acids and other chemicals in them that will bring complications to me?

M: I wish we get other types of condoms. The ones we have are not safe. They can burst. What is the importance of testing?

R: It helps to be aware of my status. It helps me engage in safe sexual behavior.

R: When I find out am positive, I can make sure that I transmit the virus in the village.

R: To know if am safe or sick. That’s the main reason I go for testing. So that if I own things like goats. I sell them off before I die and buy myself alcohol.

R: If am sick. I start on drugs right away.

M: Let me first respond to that question sir. That’s why you are given drugs. As soon as you find out that you are positive, start treatment immediately before the infection weakens you.

R: Let me also ask, if you test and you find out you’ve been positive for only one month. Do you get healed completely when started on drugs?

M: Let me respond to that also. Some will call it healing and others will not. When you are infected, the virus retreats to the bone marrow. When you start on drugs as soon as possible, you can weaken the virus before it goes to the bone marrow. That’s why sometimes you can test positive and negative other times. You also asked that why do you test and find that sometimes you are positive and other times negative. ARVs make HIV dormant.

M: The other question is. What do you think can motivate men to go for testing?

R: The main reason is unless you have had sex with many women, there is no way if you’ve dated only two girls that you will think about testing.

M: Any more reasons?

R: Sometimes when you contract skin diseases, you might go for testing. Is true that if you get a skin disease you are HIV+?

M: I will respond to the question later.

R: If am not sure that my wife is faithful. I can travel and she sleeps with other men. So I have to always test.

R: Sometimes you can test because your friend has tested.

R: The other reasons is the introduction of community outreaches. This will cause a man to get tested as they often have no time. It also helps men in hard to reach villages. Government should introduce monetary incentives to motivate men to go for testing.

M: Let’s go ahead. We have been talking about the topic of HIV testing. We have asked so many questions and you have given so many responses. Is there anything you would like to add on to the reasons that stops men from testing for HIV? Ask questions too.

R: If my wife is pregnant and I am HIV+ but have other STIs and I am on treatment. Will she get sick also?

R: When a woman is pregnant, there is medicine that helps to stop mother to child transmission.

R: Why don’t you make a drug which you use so as not transmit HIV/AIDS.

M: I told you that when you are HIV+, the virus has its reservoirs in the bone marrow. That’s why they give you the drugs to weaken and reduce its multiplication. The drugs also prevent mother to child transmission. That’s how it can prevent the child from acquiring HIV even when the mother is HIV+.

M: What about men? What situations can stop us from going to test?

R: The ARV tablets are very huge. They can worry you. They are nicknamed nine millimeters. I also fear to die when am told that I am HIV+.

R: When I go for testing, I may find someone from my village who can go and tell everyone that am HIV+ if they get to know. The youth fear embarrassment. When everyone gets to know that you are HIV+, they will invade your privacy. The youth will be forced to go around transmitting the virus to others. Also people are very poor, if you tell them to go and buy the drugs, they won’t.

M: The drugs are free at all government hospitals. But they are of limited.

R: But the health workers sell government medicine.

M: Now that we’ve exhausted all these issues. Let me call upon that gentle man to come and teach us about the use of this thing if he can remember.

R: You see this thing. It has a procedure on how to use it. Procedure one to four. When you open it and you put it in your mouth, it takes only two to three minutes. When you remove and find its signaling only one bar, know that you are negative, but if it shows two bars, it shows that you are HIV+.

M: This man has made a few mistakes. Let me try and explain to you how to use this. This method is called HIV self-test. There is this paper. Look at it keenly. It helps you to test yourself from all places of your convenience. It is different from all other methods of testing. It doesn’t require an injection. It uses your saliva. You should wonder if there is HIV in saliva.

M: Saliva doesn’t have HIV. You won’t actually get infected by kissing or sharing saliva with anyone. But the reason we use saliva is because, when the virus enters your body, it releases antibodies throughout even in the saliva. When you are to use this method. You are given a packet that contains three things. The instructions are in both English and Luganda. You have to read and understand them very well. Today, it’s in black and white. There is this spoon you see, secondly there is a bottle then there is a base. That’s where this bottle sits to prevent the chemical in the bottle from pouring. Those are the three parts of this packet. You must be careful on how you handle this packet. When you hold the device and place the flat end in your mouth, you spoil it. There are these pictures of instructions on how to handle it. Don’t put it the chemical before you put it in your mouth. You should follow the instructions from one to four on how to handle and properly use this method. Do you know why you should put in only in the gum but not inside deep in your mouth?

R: No we don’t know.

M: We use the gum because saliva there is clean. Use it before you eat food. When you’ve eaten, use it after thirty minutes. It’s better when you use it after waking up in the morning before you brush. Use it as instructed by the pictures on it. After twenty minutes, you remove it from the chemical and read the results. Look at the result chart. There are letters T and C. You should understand what they mean. That’s where we get our results from. When it shows red bars on both T and C. That result is HIV+. An invalid result is when the red bar is only on T. C means control and T means positive. C alone shows that we are negative. So the valid results are only two. When showed C only and the other is C and T showed at once.

R: Does this kit work only one time?

M: Yes, sir, you can only use it once.

R: Are there any side effects?

M: No sir. There are no side effects.

R: Are these things free or we have to buy them?

M: For now, they are free. Am not sure if we will continue getting the funding. What do you think people will thing about this new method?

R: People will like it because people fear injections.

R: It ensures privacy too. It also allows us to train our friends on how to use it.

M: We have implementing partners who help in the funding of this method. For now, they are for free.

R: People will love kit because it doesn’t waste time. You test yourself without anyone knowing. It will help reduce on the long waiting lines at the hospitals.

M: Have you ever heard of this method?

R: No sir. This is our first time.

R: I will use it at my convenience. No one will know my status. It will also save people money they spend on transport.

M: So, what might stop people from using it?

R: People will think it’s for buying.

R: Others won’t trust it because people have different ideas

M: What could be the benefits in this method.

R: It will help us know our status.

R: It will help not waste our time.

M: Do you think men will find any problem using it?

R: They will not find any problem.
