# Supplementary material for: Prevalence, associated factors and perspectives of HIV testing among men in Uganda
Source: PLoS One. 2020 Aug 7;15(8):e0237402. doi: 10.1371/journal.pone.0237402 (PMC7413494; doi:10.1371/journal.pone.0237402)
Supplement: S1 File — (ZIP) [file pone.0237402.s002.zip › manuscript data/FGD Kayabwe 4 Eng.docx]

M: A born-again not a born-again in the daily situation, let me lead in a word of prayer, Lord our Father we kindly ask you to lead us in what we are going to discus, everyone to give out their view that they want, that our gathering may benefit us even those who have not come here, Amen.

M: So, right now we are going to introduce ourselves and I am going to introduce myself again, and we are going to move like that up to the last person. My name I said is XX, I am a doctor from Makerere University and I am here because of health issues. My name is YY and I am from Makerere. My name is ZZ and I am from Kalangala, RR, PP.

M: Now we have two topics that we are going to discuss about. The one that we are going to begin with or the first one, we are going to discuss is about what stops us, what attracts us to go for HIV tests as men in place of Kayabwe. We are going to pass through some questions depending on what we are going to discuss about. Remember we have gathered here to discuss about what prevents as us men and what can attract us to go for HIV test. By the way the first question that we would begin with, by the way there are very many ways through which we can test for HIV that you have been seeing as you people of this place, what can you say about those ways? Again which other ways can you give about the current ways of testing for HIV?

R: Now on that question concerning the ways I thought we are on the question.

M: But we are on the question and you are the ones giving us the answers.

R: Things that prevent us…?

M: Things that prevent us or things that attract us. Depending on the current state of HIV.

R: Yes, what prevents me from HIV testing, hasn’t it been like that?

M: That is another question. The other one has been a main topic, the question is that, the other has been various ways that have been used to test for HIV, those that you know and those that you don’t know or even those that you have ever used or those that you have never used what do you say about them, how about the people in this community and even other people what do they say about them? That is the question. Now you can talk about the negativity or positivity, you know best. Remember that in this there is no wrong answer in our things every answer is wright.

R: The good thing we had last time given the fact that we are always busy, doctors used to pass around here and put tents and even one would spare 2-3 minutes but right now they no longer do it in this village they have spent like three months but it was good. Right now if you spare time and say that you are going to the hospital, when you reach Nkozi when there will be a long line and that is very tiresome whereby you incur very many losses.

M: So with the current method of HIV testing they don’t give you time yet previously they used to give you time. Another idea on this method, who has something to say about the methods that are used for HIV testing, what do you personally say about them.

R: Personally I would say that method would be good but there are ways through which things are done and you see that doctors who work in that area, when the time reaches for HIV testing they are the same people you will find in that area testing for HIV but me I may be when I am sick and at the same time I don’t want that doctor to know my issue, I may know that I am sick then he/she may go telling this person and the other. If this doctor I don’t know comes to test me for HIV then he goes back, I can go and get tested and the issue will be over but there are some doctors that come in actual sense we know them and we don’t want them to know us.

M: Is there anyone adding anything on this issue? You can give your thought, we expect everyone to have a thought on how to test for HIV, you may not have tested for HIV before but you may have an issue concerning it. That no I have never tested for HIV before but I don’t like what they are doing or that the situation that they pass through to test us is not ok because of this and this. What do you say about that issue? According to current ways that are used for testing HIV, you as a person how do they prevent you or how do you see them in that way? Do not fear if you have an answer to bring it here because we have told you that there’s no answer that is wrong, concerning our situation what makes an answer more correct than another one is the reason you bring concerning the answer you give. Yes, please.

R: Me on that issue what I can see that prevents most people, in most cases is fear. Someone gets to fear especially when he looks at his past sexual history and the people he has slept with. Do you understand it sir? These bring him fear and then he says that if they start to test me and tell me that I am HIV+ that I am going to be ashamed. So most of them fear because fear is much concerning the ways that are used to test for HIV.

M: The men that are that side, the current ways that are used to test for HIV, what do you say about them? Ok sir repeat the same issue that they have talked about. However, try to speak louder we beg for your pardon.

R: I have been talking about the issue of not giving us time, they do not come to test us for HIV yet they were around last time.

M: But have you ever tested for HIV?

R: Me I have never tested.

M: But what prevents you from testing for HIV is it that the doctors are not around?

R: Yes sir.

M: But if they are around, would you have tested?

R: Yes sir.

M: Gentleman what do you say about that issue, on the issues or on the ways that have been around concerning HIV testing, what do you say about them?

R: That method is good but I don’t want another person to know that I have tested for HIV.

M: Meaning you want a secret method?

R: Yes and If I test and I have it, I can find the way I can treat myself.

M: Are you ready to take medicine if you find out that you are sick?

R: But just because you enter some things when you are not ready but, when you find yourself HIV+ you start taking medicine right away.

M: Why do you start taking it? I have not heard any word from you.

R: Me personally I am a bit different from the other one because the nearest hospital here is Nkozi. On Tuesday, there is always a market so they come and make a tent, recently they were doing it every market day on Tuesday and they were always around but we found out that the health workers who treat malaria and other diseases are the same people on the team of HIV testing. So you can develop some fear and you say if I am sick, and the other health worker tests me he will go on spreading the news yet in most cases, we date their patients. Once you are found HIV+, they can try to control you because they also have daughters.

M: Do you mean you can develop some fear that the health worker can say that you are sick and tell his or her daughter to prevent you from befriending them?

R: To say that he/she can do all it takes to prevent his or her children at home just because he/she knows you so they can’t come to you. He/she may say it says it to the family at home, then the kids the take it outside family and in the end the whole town gets to know that you are HIV+.

M: Now basing on that issue that you have given, what would have been done to solve that issue?

R: What would have been done to solve this issue is that hospital in Nkozi should create a department that deals with HIV+ people only and other departments to deal with other diseases in that hospital. The government should also provide more health workers in that hospital so that issues like this one kept separate in one away from others.

M: Other people what do you say about this man’s issue?

R: Testing us for HIV wouldn’t be bad because everyone would love to know their status. Now you have said that you are a health worker, do you think you can have two more years than me? If I ever came to hospital and it so happens that you are the one going to test me for my HIV status, you stay in Nkozi and I stay in Kayabwe and I run all my businesses in Nkozi, if I reach in hospital and you are the one inside and I very well know that I will come back tomorrow and I see you, you can’t test me for HIV because we date girls in the same place simply because you are a youth and I am also a youth. It is can be easy for me not to come to that hospital to treat me because of ignorance. But if it so happens that a person is to test me, you have to see that people are different when that person is older than me, because you can reach hospital and say those are youths who are not trustworthy but when you find a grown up person you can give them their respect. But if you happen to look carefully most health workers that go on spreading rumors about people’s statuses are youths who are still young of about twenty years and below and that is another problem and it is a big one. Now if the other man has been telling you that if a person has tested him for HIV, I don’t think there is a doctor who is thirty, forty or fifty years who will go on telling people that the other one is sick. But if you are in the same age bracket, you are always dating from the same people. Another thing if you find a doctor let’s say he/she comes from Kiboga or Kampala and you bring that person here to test for HIV, such issues remain between you and that person. Therefore, he does not have anyone to tell about that issue and it’s the last time to see you. But, for a person who has a clinic here, I will come by to buy Panadol and he will tell a patient that he is HIV+ but he does not know that he is sick now he has come for Panadol tablet instead of ARVS, do you see that? Then that kid can go on telling the whole class and the whole class gets to know that a person is HIV+.

M: Others what do you say about this gentleman’s issue, I have liked it lets first complete it.

R: For that issue this is what they can do, the health workers from Lukaya can come and test people in Kayabwe, and those in Kayabwe can test people in Lukaya, so that issue can be safe. A person can’t fear because a person has never seen them anywhere, he/she can go and test people from the other side and the health workers from the other side come and test for blood of people from this side. There people will always take an initiative to come and test for HIV because they will not have a reason for fear and again I can’t fear telling you my issue because I don’t know you and if you know me you will keep quiet you won’t tell it to your colleagues because you don’t know me well so it will remain between me and you.

M: Is there anyone having anything that cannot be left out on that issue, or we should proceed?

R: What this one has said wouldn’t be wrong for the reason being, if you put there a tent and I get to know that you are from Nkozi and it will happen that tomorrow you will see me, you can’t make me leave my job at least I board and go to Kampala.

M: Ok anyway we thank you for your contribution on that question. Another thing these days more ladies have taken an initiative to test for HIV than gentlemen, what do you say about that issue? Or what are your views on that, most ladies have tested for HIV more than gentlemen, is it true or not? It’s right. What brings that, or what do you say about it?

R: On that issue I would say like this, that men have a mistake that women also have, you will get surprised that if a woman loves you and she is HIV+, she doesn’t tell you that she is sick and for you, you think that the woman you are dating is HIV- and more to that she goes ahead and gets ARVS, you only see her becoming big and beautiful when you personally you don’t know your status.

R: Now another thing, men always want things that benefit us, if I see that they testing for HIV, I just say those free things where do I go? But if you hear that they giving out something there you will get surprised that a man is the first one on the line. And if at all they can say that they are to give out money, you will get surprised that women who have been the first to line-up will be displaced by men as the first people because men want to benefit economically more than their health.

M: In short do you say that to get these men we need put there an offer of something?

R: Yes, because the youths of this regime want money, so this time round if you come and say that there’s testing for HIV but any person who is going to leave this place is going to walk away with something even if you have not said it but you say something, you will get surprised that initially you have been testing for HIV for like ten people, you are going to test for like thirty people because of something you have said but women are always up to their health. That is the reason ok what I blame women for is if she is sick she doesn’t tell you even men don’t talk but for a woman she goes and sorts herself and she rescues herself and she leaves you to also face the problem alone.

M: Sir is this one yours or for your friend?

R: For my friend.

M: Ok have you ever tested for HIV? Ok do you accept that women are better than us in testing for HIV? Or you are against that? Are you saying that they are better us or you are the ones better than them?

R: Ever since I have lived I have never heard that a girl has dated a boy but they say that a boy is the one who dates a girl.

M: Now let me ask you, among the girls you have slept with, how many have you asked for their proof of HIV status or how many have asked you for your proof of HIV status?

R: I have never asked any.

M: How many have asked you?

R: No one has ever asked me.

M: Why don’t you ask them?

R: Me I no longer ask.

M: Do you measure using eyes?

R: On that point, the first point why they are more than us it is right, it’s time for them they have much more time compared to us men. You can have your family with a wife and children and you think about sparing the whole day because it’s almost a full day, because this man the point he has first put across that we don’t have enough health workers, you can go and line up, by the time you leave that line you may find that you have very little time left, meaning the whole day is gone, your family needs something to eat and you find you don’t have time. Another thing you can be with your wife at home and you say that my wife I want us to go for HIV testing, now that you don’t have time, so for she faces it at that real time that she is the one who is HIV+ and for you, you are HIV- and then she thinks that if we stay together and he negative and I am positive, how is it going to be? Now she puts herself on pressure, the moment you leave like this if you have gone to work then she uses that chance and she goes to hospital to be the first to know her HIV status. However, testing for HIV we always want but the main issue is time. Because if you go off your work at around 6pm, you find the health centers closed.

M: Is there anyone adding on that point?

R: And again those women, one woman because you also see the current situation there’s too much poverty and the poverty that women have is different from ours because for us we work you can get what you want but for women their earning is less. She uses that chance because of the very many people she comes across and she loves many men like three in one week or four something that is not on the side of men. For her she uses that opportunity because of the very many sex partners she has which forces her to go to hospital. However, me I go for HIV testing and I spend one month because I am not into casual sex. I can spend one month when I have loved one girl and again if it is one I have just met I protect myself so it makes me be like….

M: My dear what do you say about it? Why do you think that women usually go for HIV testing compared to men?

R: Again everything women, it’s right they test. Now for me I can leave home 4,000= UGX but in most cases it is not enough for the home and when she realizes that she is going to use many things and there happens to come this person and he deceives her that he has 5,000= UGX, she will just accept him. But for me I don’t have another girl I go to and I don’t give her money for that matter I keep myself in a poverty life, but everyone who comes with money she goes with him.

M: Don’t you have any other thing that prevents us from going for HIV testing apart from the issue of time and money?

R: I have been saying that something that would prevent me from going for HIV testing but when for the woman it allows her go for HIV testing or it makes her go for HIV testing. There is a trick for health workers I also see it in my place the other side, a woman or a man loves the other and tells them that she is HIV- but when she is HIV+. Then she/he goes to a health worker if the result looks similar to the first one when she goes for an HIV test when she/he is sick then he/she tells the health worker make a document that indicates that she is negative instead of indicating that he/she is HIV+. When you come and start to love that woman or man they will show you their document which is HIV- when in actual sense they are HIV+ but she brings HIV-. When you search deeper, you may find out that she/he was HIV+ changed the document. So now even if you caned me, I can’t go back for HIV testing in that hospital that is near there. That it has prevented me from HIV testing.

M: This question comes to you, how can we get rid of that situation of not trusting the health workers that this one gives out right results, this one gives out fake results and again that health worker who gives you correct results how would he/she look like, and that one who gives out wrong results how would he/she look like?

R: According to me on that point of health workers faking results and he she says wrong results is as a result little money and the bad environment they live whereby a health worker is on government pay roll and the new month has come and no salary, and then he/she uses that chance not to mistreat but to benefit because then the other one gives him 20,000= UGX because of their working environment. Also, in most cases the monthly salary has come and he/she has not paid rent or they have even sent children back home for school fees because the fees were not paid in time, then he/she uses that chance to solve his/her problem. The government has to come up with strict laws on that issue and even to give health workers their salary in time. If a health worker works for a month without being paid, they end up doing the contrary and hurting others.

M: Is there anyone saying about this man’s issue?

R: Even me what I am sure of me I can go for HIV testing but if I discover that I am HIV+ and the girl I am dating says we can’t proceed if we don’t go for a medical checkup, if I decide for her the hospital to be used and I happen to use the chance of money then I make that health worker make a mistake. My decision will not differ from this one’s.

R: However, according to me I would say that what would stop all that is a health worker from far can solve that issue because he won’t know you. He/she will fear to be tempted to do this and that thinking that person may be a spy. That is one. Secondly, the longer the health worker stays in an area, he gets used to the people so they deserve to be transferred always. If a health worker over stays in an area, he gets used to the people and starts joking with people to get to know how to make money out of them. But if a health worker is like a policeman, let me say he/she has worked for six months and they transfer him/her to another hospital I guess that issue can’t happen because he/she may doubt when you say that don’t give me the result of this test. He/she will fear and say that no I have just come to this hospital they may be on spying me. But if the health worker has overstayed in that area that situation will not end.

M: Some of you told us that you have ever tested for HIV status, isn’t it? So for you, what do you think are the good things that are within testing for HIV? In most cases men including you say that when I test for HIV I get this and this, why should we test for HIV status?

R: My dear, one of the good things in that thing is that you get to know your status if you are HIV+ let’s say you have a family and little children, if you start ARVs, you live for some time to see the children to grow up. Because it doesn’t help to test for your HIV status and you stay the same. So if you start ARVs you can add more years to your life and enable your children to study.

M: Gentleman you told us that you have ever tested for HIV. The other gentleman has kept quiet for so long but I am going to come back to him, which good things have you seen in testing for HIV or things you say that before I tested for HIV like this and this but when I tested for HIV I benefitted like this and this.

R: The good thing I have seen is that I have never tested for HIV but they told me to use protection the next time I happen to have sex.

M: Do you always use protection?

R: Yes.

M: Gentleman you told us that you have never tested for HIV before, for you what do you think that you will benefit when you test for HIV more than now when you have not yet tested?

R: What I will gain after testing for HIV? Well, if I find that if I am sick, I will start taking my ARVs. I won’t accept to spread the disease to young girls to put them into my situation because ARVs are good when you are sick but they are not good if you have not reached out for them.

R: For me the importance I see in that is, it is always good that you test for HIV because most cases there are women who love us and you test them using your naked eyes and you reach a time and see that they are many, then you withdraw your heart. Now if you test for HIV and God helps you and you find yourself HIV-, you can reduce speed at which you have been moving and say that God if you give me this chance and I find myself safe I am not going to repeat moving at the speed I have had previously, let me be with one person. So if you have not yet tested you can say no let me go ahead with my thing I will test up myself in time to come. But if you test yourself for HIV and you know how much time you have left, you can change saying God has given me a chance I am still HIV- let me stop moving.

M: Thank you very much, sir what is the importance of testing for HIV?

R: The good things that are associated with HIV testing are very many because you get to know your status, where you are and where you have reached, even you normally you can decide for yourself what is good and bad. If you get to know that you are HIV-, you go ahead and protect yourself, if you find out that you are HIV+, there is some counseling that you get from a health worker and you go ahead to follow the rules and regulations in order to have an additional life and if you that it is not helpful you leave them.

M: Is there anyone adding something about it; the importance of testing for HIV? Or if you say that for me I have ever tested for HIV and what I have got is this. Before I tested for HIV, I was moving through this situation then after I tested for HIV this is the situation in which I am moving.

R: What I gained in that was that after they have tested you for HIV and they tell you that you are HIV-, you reduce the speed at which you have been moving, and even the situation in which you have been moving. There is a time you reach and you love women and say I may have become HIV+. In fact, when you are not reducing but you are just moving forward, because when you have used you naked eyes to say that you are HIV+. So now if you go and they test you for HIV it reduces your speed and you say that I won’t love this one because of my current situation or you can even decide that let me get one woman whom I am going to marry in case you have not married and you first test for HIV.

M: Anyway those of us who are here, how many are here and they are married? Are you married? Mr. XX are you also married?

Mr. XX. No I am nor married.

M: Anyway even if you are not married again it has no effect. Those of us who are here, how many have ever gone for HIV testing together with their loved ones? Now you have told me that you have ever gone for HIV testing. What do you think about going together with your loved one for HIV testing?

R: Everyone gets to know the status of the other and you go back home when you are happy.

M: What other things are in going together with your loved ones for HIV testing?

R: First of all, in that it reduces adultery at home everyone fears, for the other if you happen to go together. If they discover the first time that you are all HIV-, one says that if we happen to go back for HIV testing and I am the one who has made a mistake, how is it going to be? Therefore, that enables everyone to protect himself/herself. They may tell you that come back after three months so everyone wants to go back with the other when they are safe when they are not HIV+ saying that they tested us for HIV and we were all negative so it removes suspicion of adultery and again everyone trusts the other provided that when you go back they tell you that you are HIV-.

M: Mr. SV are you adding anything?

R: I wasn’t going to give my view but this man talked about it while concluding. That thing brings about trusting each other in marriage. It brings about trust because when we go with my wife, she may have doubted me every day but if we happen to go and they test us for HIV, she will trust me and I will also trust her. However much in most cases it brings about separations within families if you test for HIV at the same time with your wife because you may find that one person is HIV- and another one is HIV+, but if you find out that, you are all negative, it brings about trust in the family.

M: Do you have a girlfriend?

R: Yes.

M: If she wakes up one day and she tells you that let us go for HIV testing do you accept or not?

R: I accept.

M: According to you what are some of the benefits you get from going with her other than going alone?

R: Even me I would love it if we go together and we test, if any of us is HIV+, I am free to run away from her and I get another one and we test for HIV and if she is negative then I stay with that one who is HIV-.

M: Let me first ask you there after I am going to return to these ones who are married. If you go with your girlfriend for HIV testing, and you find that she is HIV+, do you drop her or you go ahead with her when for you are HIV-? She has been there for in your worst days and you have been with her for three years, others what do you say about it?

R: On that issue it depends, we may discover it when I don’t have any child with you, but when I asked a health worker it happened that you can produce more than one child when she is HIV+ when you don’t know, but me if I have a child with you I can’t drop you. What I do is to continue supporting you but because of the truth that I have discovered, I stop all sexual relations with you and we can look after our children as I give you support.

M: Now little before others give their views, I am still asking you who has said that you have gone to hospital and they have tested you for HIV and you are negative, you have children with that woman and you say that sleeping together is impossible, now how can you manage to stay, and even her how can she stay happy?

R: Now this current situation even if you are tired of a woman you can look after her given the fact that you don’t stay together.

M: No let’s say that you stay in the same house with your wife, do you leave that home?

R: I don’t leave you the home or what but I leave you.

M: But you stay looking after the children and you even come and see them?

R: It’s possible.

M: Do you accept your wife to get another man?

R: It has no problem it’s upon her and the man if it has ended between me and her.

M: But let’s first listen to this new man’s views, you happen to go to the hospital and they test you for HIV with your girlfriend or with your wife, do you have a wife?

R: Yes, sir.

M: And they test you with home wife and they discover that you are HIV- but your wife HIV+ but you have three children with her, what’s your next step at that time?

R: I just advise her to go and start taking ARVs because I once heard that if a person is taking ARVs she can’t spread to you HIV if you handle each other very well.

M: You man, if a person is taking ARVs does he/she pass it (HIV) on to others or not?

R: She/he doesn’t.

M: For you what do you say?

R: He/she can pass it on to others.

M: Sir what have you told us?

R: It depends on the way you have had sex, you can go and have sex in a forceful way in case inside her she gets damages and she bleeds there you can get HIV.

M: But your issue you have said that she can pass it on which means you have spread it.

R: The other when you can agree when there is no force but peaceful.

M: This one told us that she can’t pass it on.

R: Now for me I am saying because I am always on radio and health workers who are looking for medicine that cures HIV I hope they have not gotten it they have the one that relieves, now for you how do you say that a woman can take medicine that can relieve her just but that which does not cure her, how can you say that she cannot transmit HIV yet she is not cured?

M: But let’s take that question to this gentleman, and he can become the first to answer. Have you said that the one who is on ARVS doesn’t transmit HIV? She doesn’t?

R: Me personally what I know is that if a woman is taking ARVs, and when the man is HIV- and when everyday she takes ARVS her disease is very hard to get transmitted because her infection can be dormant.

M: Now you have not separated is it difficult to get transmitted or it is not transmitted because you told us that she doesn’t transmit?

R: It is not transmitted only if she does not miss the ARVs every day that she is supposed to take medicine.

M: What if there is what this one has said?

R: For me I am going to give this one’s answer, a person can take ARVs, can reach at around 80% he/she can take ARVs and it goes on weakening slowly by slowly.

M: For you what do you say? Does she/he transmit or not?

R: Let me also ask as I have a wife and we go for HIV testing when we have three children and I find out that she is HIV+ and I am HIV-, which advise do you give me let me also ask you?

M: Which advise do I give you? Is there anyone who seen a person saying that she/he was tested for HIV either a man is HIV+ and a woman is HIV-? Is there anyone who has ever seen that person?
R: I just hear about them. I have ever seen that person.

M: But do they stay together? However, when a man is HIV- and the woman is HIV+ and they are still engaging in sexual intercourse and don’t they even use condoms? Is it possible or not?

R: It is possible.

M: Now first answer yourselves. Little before that, we are going to bring that question in a little moment, what if the two of you for HIV testing and you find yourself HIV+ and when the woman is HIV-, how can you go about that situation?

R: When I am HIV+?

M: Yes, when you are HIV+?

R: Personally according to my situation, I would give her freedom to choose what she wants. If I have children with her or if she is a girlfriend, I quit that and I give her freedom to choose what to do for herself. I leave her it to her to decide, if she decides to leave me I don’t take it in a bad faith, if she decides that we stay together I take it as she has decided.

M: What have you been saying gentleman?

R: I have been saying that in case your wife is HIV- and you want to leave her, you can never know that where you are going because you might instead pick on someone who is HIV+ and you end-up falling in to a ditch when you are seeing.

M: You said before that you used to test for HIV using eyes.

R: We use eyes but if I know it…

M: But do you know that women who are HIV+ look more beautiful?

R: It is right. But if I get a chance of testing her and I find her HIV+ when I am HIV- the fact is I can’t because I can be tempting God.

M: So what you talked about the children, wouldn’t they suffer if you say that you have left it to her to decide?

R: How do I make them suffer when I am going to look after them?

M: What about the love of the children?

R: The love of the children; we can be saving them.

M: Do you think a woman who is HIV+ can produce a child who is HIV- or not?

R: She can produce that child 100%.

M: What is the reason?

R: The reason is you can go to hospital because health workers have a way they do it and the pregnant women who are HIV+, even my brother’s wife is HIV+ but they have a way they do it and they produced a child who was HIV-.

M: What do others say?

R: You can obey the rules of the health workers and you do it as they have said because they know that it works.

M: Now let’s go back to the other issue when the man is HIV+ and his wife is HIV-, can he transmit HIV to her or not?

R: He can transmit it.

M: But why can she give birth to a child who is HIV-?

R: If at all you obey the rules of health workers.

M: But now little before that you have come with your wife to test for HIV and you are negative and the wife is positive, is possible for us to convince you that you stay with her you will give birth to children who are HIV- and you will stay HIV-, can you accept that?

R: Now doctor if you can look into that issue.

M: ok I can explain it to you can you accept it?

R: If at all you can explain it to me and I accept that the child will be born HIV-. Now we have also asked you here that is it possible for a man to be HIV- and the wife is positive and they give birth to a child who is HIV-. In addition, you have accepted that yes, then why don’t you just stay with her?

R: Can you step in a ditch if you have seen it?

M: Gentleman what are you saying?

R: What the other one is talking about is that you can be HIV+, and the woman is HIV- and you can be with her and even have sex, but it depends on the way you approach it. If you use have come, you can come with force and either of you gets a bruise then there’s blood sharing which increasing chances of being infected. I don’t really understand the way you have such sex.

M: Ok now as I am concluding in addition to this gentleman’s point a little bit because we are going to another point, haven’t you ever had a person and they tell you that the other woman is HIV+ and she is on ARVs, and one rapes her and he doesn’t contract HIV?

R: It is the type of blood that the other one has. Or he can date her and they don’t have sex.

M: And they don’t have sex? How?

R: The reason is that person can so happen that he has not gone into the other issue that brings about HIV. I had a girl they told me that she was HIV+ and I had sex with but I didn’t contract HIV.

M: And at last did you confirm that she was HIV+?

R: Yes.

M: At last did you confirm it? Ok let’s leave that issue people. Ok we have seen the importance of testing for HIV, but what do you think are the things that attract us to go for HIV testing, men what do you say or what do you do that attracted you to go for HIV testing, what attracts?

R: For us we mostly stay with youths. They see where you date, they see your movements, for me what first attracted me to go for HIV testing is, you can go to play pool and people can say them in a joking way that according to this one and this one, you are already done, when you are still there in the middle of the conversation they have their saying that you slept with someone who was HIV+, do you understand?

M: That what did you eat?

R: Someone with HIV. Now you first take it as it’s a game when you are still there but when you go back home you say that the truth is the colleagues talked about a fact, this one and this one let me go for HIV testing and sometimes they find you HIV- or when you are positive and you start taking ARVS, if you are negative those ones who seem to be with HIV you leave them and you cut them off and you say that let me stop those ones.

M: I think even you; you have ever tested for HIV? What attracted you to go HIV testing because I know is that you were like the other one you have never tested for HIV, but what do you say that in fact it is this that attracted me to go for HIV testing and that if it weren’t that I would never have tested for HIV.

R: What attracted me to go for HIV testing is having sex because if I didn’t have sex I wouldn’t have gone for HIV testing.

M: Having sex? Every moment you have sex do you go for HIV testing?

R: No.

M: Now if you start right now this hasn’t this one ever had sex? Now which other reason apart from having sex attracted you to go for HIV testing.

R: Another reason that attracts me to go for HIV testing, recently I used to get girls when there was no way I would protect myself because I tried to protect myself and sometimes things failed to work out, then I said that the wise person keeps quiet. Me I don’t accept protecting myself because I know my HIV status, but today when you date a woman and I sleep with her, for me my policy is that I have to test HIV with her. Because me I can have already had my wife I don’t always use a condom that’s what attracts me to test for HIV every time I get a woman.

M: Do you test before you use her or you test after you are done? As I conclude, do you first eat and you wash your hands thereafter?

R: Personally, if I sleep with you because it’s not every day that I stay with you, if I spend like a month or two to get another one.

M: Now let me ask the same question on your side we are going to come back to you, that woman how long does it take let’s say you have slept with her today and you say that let me go for a medical checkup?

R: It takes three months. For me what would attract me to go for a medical checkup is to be with my wife when I don’t know her movements. According to the way we move, you happen to be moving and you hear your fellow youths saying that I saw that one with the other one and the other one, it attracts me to go for HIV testing.

M: What do others say?

R: What I say the thing that attracts me to go for HIV testing, you can have your wife and the youths talk about her as a person who sleeps with everyone because in a month you sleep home twice, now if they love her and it is the same wife that I love then I have to go for HIV testing. For me I take it every after a week.

M: To test for HIV? Or little before that let me return this question to you, first ask him and I also ask him, do you know that you can have sex today and you become HIV+ after five months? Do you accept that or not? It’s what this gentleman is asking. That if today you sleep with someone and you test for HIV tomorrow you may not know but if you test for HIV after like five months that you can know?

R: That’s why I want to know…

M: But do you accept or not?

R: I accept that’s why I have said that I do it after a week for the whole period.

M: Now let me give advice, I agree with what this gentleman is saying. However, if it’s today when you have slept with a person, is today Wednesday? And on the Wednesday of next week and you go for HIV testing, you could be wasting your time. They will pierce you for nothing; you are supposed to do it every after three months. Now if this virus happens to attack your body, it is not detected there and then if you not been positive, but it can take you between 2-3 months and for some even many more months. So it is not advisable to sleep with a girl let’s say I have found a girl and I have dated her in Kayabwe and I say that I doubted her because she had swollen things, and tomorrow in the morning I go to the hospital you can have wasted your time because they will show you that you are HIV- but on the other hand the virus may have entered you but when it is still invisible. So now in this situation a person would spend three months and then he goes for HIV testing after sleeping with a person, but it won’t help that today you have slept with the other and you have doubted her and you say that tomorrow you are going to run to the hospital again they will tell you that you are negative, spend your money without knowing the truth of how you are. Do you hear this one said that he first eats and then he washes the hands but at least he would have first washed hands to eat because what you eat is not hot.

R: Even if you do what, you reach a time and you have unprotected sex with her.

M: If you date a person today you can give it some time but the bad thing is that some of you want there and then, that in one week I want to get it done.

R: You don’t put off for tomorrow what’s set for today.

M: Ok on that issue let’s proceed, what do you think are the things that stops men from going for HIV testing? Which things prevent them from going for HIV testing as men?

R: Not trusting each other.

M: How?

R: Because I may be working within others and have my wife in Kayabwe, I don’t know what she does, she also doesn’t know what I do. Now if you are going for HIV testing and you say no when you were to go for HIV testing together, or you also say no I am going for a long journey they have called me then disappear. I have gone and she has gone to be tested, for me I have gone I may be HIV+ or HIV-.

M: People give us other things that stop you from going for HIV testing.

R: Those that stop me? What stops me most is adultery, you date women every year, I date ten women but in those women the fact is my heart says like what the others have said that there is one with HIV when I had sex with one with HIV, and I say that I am going nowhere the health workers will test me when I am on my bed, do you see that one? That one makes me fear a lot.

What stops the youths from going for HIV testing, a person telling you that you are HIV+ is scaring. What this gentleman has said that for him a year passes when he has not gone for HIV testing because he knows that he loved one woman, do you hear me clearly?

M: Where will you stop because a woman I may be HIV+, and you love her and she doesn’t make you acquire HIV but for you when you know that she has, and you stay loving and the last one you love makes you contact HIV, now if they tell you that a woman is HIV+ skip sometime and go for HIV testing, if she is negative leave her.

R: But I may fear going.

M: Now what do you fear? But you may keep quiet when you are HIV- and they make you acquire HIV at the end, do you understand it? But that is what we are talking about what prevents us to go for HIV testing but at first we saw what can attract us, but what stops us, is there anyone with a different view? But this question was going directly to you because you told us you have never tested for HIV, for what stops you from testing for HIV?

R: For me I put on a condom every time I go for sex.

M: But don’t you think there is a chance that makes that condom to get torn and you contract HIV even if you are wearing a condom?

R: No may be when they have bewitched me.

M: I am going to ask this one a question, do you know that you can have sex and you don’t discover that a condom has gotten torn? Again let’s ask him, do you say that sex is the only one that transmits HIV isn’t there any other way through which you can contract HIV/AID?

R: They are there but I am a kind of a person that I help those ones who have been involved in accidents, another thing that makes me fear to test for HIV….

M: Who has another reason?

R: I am back, what makes me fear to for HIV we have already talked about that issue it’s concerned with health workers I believe we are done with it if there is another thing let’s go onto another one.

M: Ok what do you what would be done to add on the number of men who test for…. but I think this question was also done. Because the question was that what would be done to add on the number of men who test for HIV, what would be done? You told us that health workers who are from the North should be transferred and come this side, and then the ones from this side should go to the North to test those ones from that side. Now it’s as if we are concluding this point where we have reached, is there anyone asking a question or is there anyone who has anything that can’t be left out on this issue that is within us at this time or on what we have talked about? Because we have talked on very many things, there are very many that we have discussed about you may have something that you have not understood clearly or you have something that you want to know and others. Is there anyone with a question and we answer it at once?

R: According to what you have taught us have mastered very well now we need guidance from you people who know things.

M: Ok before to add a little bit to this man’s issue, let me begin with the issue of lunch; that’s why we moved with a soda and another thing is there anyone who has not taken it? The situation that is bad is there are some whose religion does not allow to eat certain things but they won’t chase that person from the party. What can be done if we were to talk for the whole day we would have given you lunch but it is not much time, others spared much time and answered most of the questions.

R: But what I can advise you to do is that in us those who date very many girls like this gentleman who has never tested for HIV, let’s try to use condoms. And another the issue of having very many women is not good because you don’t know her movements because you will be with one person or you will be with very many women but on those three you have and again there are also three women who have other people and even that person will want to do it when they want to revenge that you personally you have three women even me I have to get three. So my friends if we move in that trend HIV will never go away when our main point is to reduce AIDS because it cannot get over but we can reduce it. And again being with one who also moves around, what is better every after three months we can go for HIV testing. And most those ones who have wives even you who has a girlfriend it doesn’t stop you from going with her to hospital, it is good for all you to test for HIV and everyone gets to their HIV status because the other gentleman told us that if you go the two of you and you test for HIV when you have tested the two of you will say that this one is the who brought the virus or I am the one who brought it and that means that everyone will doubt the other. But let this gentleman take you through the second session but we are going to be very fast those of us who want to go should not get worried. This gentleman what they told you about you understood it very Cleary; now first tell us what you heard that is where I am going to add from.

R: This paper what they taught me about is that the man told me that do you know it very well that you can test for HIV when you have not gone to any hospital? And I told him that I did not know about it. He taught me very well and he pulled out for me this thing.

M: Discuss for them what you understood what do you remember?

R: He told me that this bottle you can open it and you get it out of its cover and there after you open it, there’s a thing you can use this side up and you and it traps saliva for this side but the one that is not from the mouth the one which has eaten food but this one from up the mouth and you it into that bottle. Now if you put it into that bottle, that bottle can show you HIV if at all you are HIV+, if you are HIV- it puts one arrow, but if you have a question mark it brings two arrow when they are like this.

M: Ok for the interest of time let me begin from here this man can remember gentleman thank you very much to learn, thank you for remembering all that. Now I think everyone with this paper, do you see it’s in black and white not because it’s like this but in the other kit we are going to give you but the paper that is in this kit or the directions, these pictures are in colored very well but because you are very many we had to remove it and we photocopy it so that we give each and every one of you, you can say here I don’t see them clearly, but I am going to explain to you, the ones on this paper be not be of use to you. So now I am very sure that immediately I have given you this paper everyone has first seen the pictures, which shows me that Ugandans don’t follow rules and regulations even the sodas you have taken no one has read the expiry dates, is there anyone who has read it?

R: For me I read it.

M: The reason as to why some of you didn’t read it…Ok I am going to explain to you the new way those of you who don’t know it that we were in this area before, we were in this place I think one week before. You have heard the main points we have been discussing with men on HIV they are some of the questions that we began with, and another thing we were explaining on the new method that the minister came up with or the one that he wants to come up with in addition to the ones that have been there before which have been used to test for HIV not to remove the ones that have been there before but to add on those ones that have been there before those ones you know and those ones that you don’t know that are used for HIV testing. The difference with this method is that we are going to be using saliva like the ones that have been there before it’s not going to be for piercing, people what is going to be using? We are going to be using saliva from our mouth to know that we are HIV+ or not. I can see questions facing me, how? Now here they tell you that procedures to follow I have started going through, they have told you that you have to follow clearly the directions of this method very well with maximum care in order to get the right results I will expect questions after explaining this thing. They have told you that don’t drink or don’t eat for at least fifteen minutes before you use this method of testing for HIV, you shouldn’t forget that this method you are the one who is going to be the health worker, you are the one who tests yourself, you have been to saying that I have gone to Nkozi for HIV testing, this time round you are going to test for your HIV as you an individual and you get to know your HIV status when you are alone. They have told us that don’t drink or don’t eat anything for at least fifteen minutes or thirty minutes before or use things that are used to clean the mouth like delident, close up (tooth paste brands), have you understood those ones? They are the reason as to why they give you that time, for the reason being that we are going to use our saliva from the mouth, this thing doesn’t deal with food that saliva if you have just eaten food, that food can be still stuck on the teeth, where? So we need get that saliva that is pure which we are going to do what? To test. There is another warning here, instructions are very vital in this thing, that if you are HIV+, you may get wrong results, who thinks like this?

R: What have you said?

M: If you are HIV+ and you use this method you can get wrong results. Who has an answer to that? You may have used it wrongly, that is one answer, who has another answer? If a person is on ARVs and he/she uses this method may get wrong results, I expect an answer different from this gentleman’s, and you are the ones who talked it about long time, that if any person is on ARVs that the virus is always sleeping it can even disappear for almost a year and the person can be stopped from taking ARVs but when it is there hidden because of this medicine if he/she takes it in time, aren’t you the one who said it?

That is why we advise people if you get to know you HIV+ that is not the end of the world, go and get ARVs that is why we said that people who are HIV+ are more beautiful. So now finding yourself HIV+ should not bring you down and say that the world has ended let me go and hang myself or let me sell all my things I am going to die. I think we have seen very many people who have taken ARVs for so long like 20 years and that person is still alive. Again AIDS is not the disease that claims people’s lives mostly, malaria is the disease that claims people’s lives mostly, did you know that?

R: What of cancer?

M: Cancer kills a few for you do you know ten people with cancer? What of the ones with AIDS? Ok we are going to proceed I am on direction number one, you will find some Luganda which you don’t understand but I am going to be explaining, they have told you that bag which has this ring for HIV testing has two other small bags they did bags but this is what they tried to mean, it is as you have your pocket on your pair of trousers, but when you have two other pockets, let the trouser of this gentleman give us a good example, he has one pocket here but again he has another small pocket here where you always put coins so again this is what this one means. On number two they have told you that tear and open one of those two bags that have a bottle. Who has a photo on the paper that is clearer showing those two bags? But what they are trying to show I don’t know whether I can get the one which is clear. Do see something that is like a spoon one those I think it is seen clearly here one of the thing that is there looks like this one of the things in those two bags. Another one that is in that first bag actually this is in the first bag this one that is like a spoon that is in the second bag there is a bottle which they have told you to open on number two. In that bag remove a bottle, I am on number two again they show us here when a man is removing the bottle the picture is on number two. After removing that bottle they have told you to open and remove the lid I am now explaining number three now, if you remove the lid on that bottle, that bottle they have told you that you place it upright in its trolley not in the trolley where you hang your utensils, you may be confused I told you this Luganda may confuse you. Yes it’s like you get you TV and you put it on a stand that is what they are trying to mean that you get that bottle it has where it sits however much they named it a trolley but I think it would have been a stand after doing that we make it sit into the trolley, we have a reason as to why we make that bottle sit into the trolley, that bottle has medicine in it we are trying to avoid powering that medicine in that bottle that is the reason why we put it on its stand which they name a trolley so that it becomes firm, are we together? Let that doctor not take all your thoughts we work with her we shall give her to you to greet you, that bottle stays in the stand, which they named a trolley on, number four. Now we are going on number five, number five we go back on number one on the bag that we have not opened, it has this tester, which you have seen that has a spoon. That is the tester we use to get saliva and again that is where we use to see what has been seen in that saliva, how? This upper part, which you see, which is somehow bigger that is where we read from our results from the saliva of person whether a person is HIV- or positive. This other smaller part that you see going downwards that has something like a spoon that is what we use to get the saliva. But we don’t just spit the saliva I want you get it clear and put more emphasis on it, don’t just spit it in this bottle, we hold this thing while holding this upper part where I have told you that we read results from and we pass it through on the upper gum do you see picture number five and number six? We pass it on the upper gum once I think you how arrows move? We pass it in the upper gum and we also do the same thing on the lower gum, I guess those pictures are very clear. After doing that again you hold this way up what you have to get scared of is one thing, you don’t have to touch this thing that looks like a spoon that you passed through your mouth or that you are about to pass through your mouth. Remember that thing has saliva so it doesn’t need any dirt or even when you get hold of it, it gets expired. That is why you see that we said not every time you have to use it I don’t say that immediately you have taken you food because if you use immediately you have eaten food it will go with food. That’s why the give you that time of fifteen minutes. Again don’t use it immediately you have blushed your teeth because the paste can have stayed in the mouth so even if someone smells into your mouth can hear the smell of tooth paste but when time passes it goes on disappearing from the mouth that is why we take saliva from up because someone will ask themselves why don’t we put it underneath the tongue? The tongue most time after you have eaten food it remains on the tongue but again another thing the glands that bring saliva are on the upper gum so saliva begins from up but again it first collects itself from down, do you get me clearly? Now after getting our thing, which we have passed on the gum, we don’t have to hold even its removal from the bag in number one matters a lot. Look clearly on picture number five there is a picture that is not clear but it is in the extreme corner it shows you when he is removing it when he is holding on this upper part which I told you about where you read results from. Avoid holding on the spoon. Now after removing this saliva we use our thing on number six when you have passed it on the upper gum and the lower one, we hold bur we hold up which I told you about on the bigger part and you put on that bottle that has medicine which we put on its trolley in number four and they show you here in number seven when he is putting it, they have told you that put it until it has topped on the bottom of that bottle with medicine. After doing that they show us in number seven when he is putting, in number eight they show you when he is done with putting it when it has touched the bottom of the bottle. We time it for twenty minutes, you time it for twenty minutes and you remove it to read the results from that blood. But there is a warning here they have said that don’t read the results exceeding forty minutes you will be reading wrong results this thing they timed it when you have to use it between twenty or thirty minutes or even thirty-five or forty, if you exceed those minutes you will get wrong results and you cry for nothing even consoling yourself. That’s why you see that instructions are very vital on everything some of took that soda when you have not read the instructions. You time twenty minutes it would be very important when they reach twenty you remove it.

R: Now how do I get to know that I am negative or positive?

M: If you look at the tester number five it has two things as you can play a song on a DVD on the next part, have you seen them? Now open behind and I show you what they indicate, those things on one of them has two, one has letter C, and another one letter T, this is what you have been seeing the other side, those two things have letter C and T. One of the letters C means control which would be translated in Luganda I think “okuziyiza”. This letter T means test what you are testing for the virus that you are looking for in your saliva, now if you find that these two letters have been underlined below them after twenty minutes after you have removed them when they have been underlined two lines and they will be in the red color even if it is pale but when you can see them that is HIV+ in simple terms that person is HIV+. If it shows you two lines on letter C and T, that person is HIV+ but even if they appear badly the important bit of it is that the two lines are there because they may come when the lines are pale, you are there found HIV+. Now on C only, explain it. Now if it appears when only C is the one which is underlined when T is not underlined because when we go onto the second part down here, are we seeing here all of us? Ok in the second if at all C only is the one which is underlined when there is nothing on T it means you are not HIV+.

R: What it is on T and it is not on C?

M: If it is on T and not on C, who can answer that if it is on T and it is not on C?

R: It is the same.

M: No, it is not the same. Meaning if it on C when it is not on T you are HIV- but if it is on T and it is not on C it means you are positive. Now if it is on T and it is not on C you can when you positive and again you can be negative and it means you may have failed to follow the instructions.

R: What if all of them don’t show at all?

M: Again you may have used it in the right way. It can be faulty itself which you used, do you understand? For it itself it can be faulty when it is expired, or when you exceeded the other minutes which we talked about when you have worked within fifty minutes. You went and tested yourself and you first went and played pull, and you played like three games when you have not gone back to check what you first did.

What have you been on? On the dead or alive?

I have been on the dead, but it is not the dead it is the patient.

Have they told you that it is C only that has been underlined it is the only being seen the one for control the reason why T has not been underlined this time round is that the viral antibodies we have been looking for in your saliva has not been found, so I want you to be keen on that reason. But if you find that you have tested but T is underlined, this time round what do you can be the cause?

R: That question is the one they have been asking.

M: Have you given them the answers? Yes, I have told them. Is there anyone who wants to know the right answer?

R: We have understood it.

M: Ok.

R: What if it doesn’t show at all?

M: You may have used it in a wrong way or it may be expired.

R: Now what are you supposed to do?

M: You have to get another one. You have to get another one.

R: What if it refuses?

M: If it refuses? There we have to force you to go the big hospital, the bigger hospital.

R: Now what I am asking when does that thing stop being used for testing?

M: It only works once and you get another one.

R: Do you have them, did you come with them? Are we going to take them we test them or you are going to tell us where we buy them?

M: Now little before that, at the beginning this gentleman it is good they have ever approached him, we sought for individual s’ views on that thing and we used to ask them where we would find them if we have brought it and where do you want to use them from according to his views. Now this thing we did not come with it but we shall come with next time and we teach you how it works and we direct you where you can find it. But the question is; have we learnt how it is used?

R: Yes, we have learnt.

M: If we bring it and we put it here? You can say that we have leant on these papers or on the new method, what do you think about it?

R: I have a question; can we use them two people?

M: No sir. You use it alone because even the syringe is pierced once.

R: Now what I am asking is that does is it show the results there and then?

M: They don’t even reach forty minutes.

People we are on the discussion of this new method, what do you think about it?

R: For me what I think about that new method it helps us a lot every one when wants to test for HIV or I don’t have time it will help us.

M: What do others say about this new method?

R: For what I say is that it is good because us the youths let’s say that you have gone to the club and you happen to pull a woman in that club so it does not have time, even if you say that…but if she has taken alcohol? Does everyone take alcohol?

M: But you can wait for her. What do others say? The new method what do you say about it?

R: Let’s say you have had sex with a woman and immediately there and then I test for HIV, can show that I have just gotten HIV?

M: But what we said at first to eat food and you first wash hands before you eat, it doesn’t have any use.

R: But listen you can know that you are HIV-, it means the moment I take you to my home I will get out that thing and I test for HIV status.

M: What is this one asking, have you understood what he is trying to ask? That you that you are HIV-, you have had sex with this woman, let’s say he is sick, can you go and test for your HIV status there and then?

R: That sex I don’t…

M: That is when we shall tell you…you yourself you told us that you test for HIV after three months have elapsed.

R: But listen to what this one is asking, for me I know that I am HIV-, if I pull a woman and I take her I don’t need to first have sex with her and I test myself. First test yourself… but you I have to first take your saliva and I get to know her status, if she is positive…

M: Others what do you say about this new method of testing on your own. Ok after telling about this method that it is good, aren’t you going to increase on adultery?

R: That adultery will be there.

M: Now what do you think are the advantages of this method? What is it after you testing on your own? You personally, when you are the doctor and you the patient. You don’t go to hospital but you test yourself from there.

R: The most important thing I see in that is that the health workers who repeatedly hide people’s diseases.

M: There is no health worker who can do that unless he/she is not well trained.

R: Then second is that, they have helped us from some health workers who hide people’s results and release incorrect results because they have given him or her money. Third, this method is going to help us the reason being we are going to save time on our jobs because if I bring my wife and test for her HIV status, I will know that I will go by it and I get another one, even tomorrow like that provide that I first test a little bit.

M: What do others say? What are some the advantages of this method? When you are the one testing yourself, you are the doctor, you the counselor? You can even bring a good thing or a bad one.

R: For me I have been coming from my wife’s place…. Saliva?

M: You cannot understand them, you are going to leave us a bit, just help us. Just listen to what they are saying.

Now what do you think are the disadvantages that you have discovered in this method? What you expect?

R: I will test in an hour and I am HIV+ and I say that let me spread it.

M: Can you be thinking?

R: Yes, people are bad hearted.

The bad reason I have seen is that this method cannot be trusted very much. It has many limitations, the minutes, do you understand? Because it is for timing like a bomb, but if you go there in the hospital and the man pierces you your finger, but here it will prevent me and at times I did not study much and I happen to forget a little bit and boost around as I…

M: Little before that, did you hear someone ask a question that how do they test the saliva when they are testing for HIV?

R: That’s where I have been going also.

M: Ok who can give us the answers?

R: The education I got for me they told me that it is blood not saliva but blood, I studied from the lowest class, I have never had that one for saliva, in most cases they told us that it is sex and saliva.

M: Let me ask you are my wife and I am your husband if I make you get my saliva, do you contract HIV when I am HIV+?

R: It depends you may have a damage on your mouth.

M: I don’t have damage and you also don’t have damage.

R: They can’t.

M: But when my saliva is infected with HIV when yours is HIV-, and why don’t you get?

R: The virus moves in blood not in saliva.

M: But this man has not understood it clearly, how can you explain that we test saliva?

R: Saliva because they are also hormones which move within blood.

M: The man has tried to bring out the question.

What are other bad things before we go to another point? How do you think the bad things can be overcome?

R: For the bad thing I see is that if you are testing on your own, you don’t get a partner because you cannot talk. Now if I test myself and I discover that I am HIV+, no one is going to remind me to take medicine because there’s no one who knows until I am weakened I fall onto my bed then they can go and test me in a known hospital then I can get someone who can tell me to take ARVs. The bad thing I see is that you and I you test get to know even if you have taken ARVs or not.

For me the bad thing I have seen in this thing in that according to my little knowledge you can test HIV when the woman you are with you were the first to date her and you test when you are positive, do you see how my decision can be bad and I cut my wife into pieces?

M: Let me ask a simple question, HIV can be transmitted from one person to another in how many ways, does it only pass through sex?

R: It passes through different ways.

M: Don’t you think of other ways apart from having sex?

R: But those other ways are too little.

M: They are little. But are they possible or not?

R: They are possible but those ways are too little.

M: But if they are there, can you think about them that your wife may have contracted HIV through those ways, or you just think about adultery only.

R: I only think of adultery only at that time.

M: Let’s remain seated we are soon ending.

R: For me my reason is because every after three months I test for HIV, I told her if you find me with HIV you are free to go to your home and even me when I find her with she goes home because I can’t be with a person who has HIV.

M: Can’t you hold for a little bit?

R: Yes, I can’t do it she even knows it.

M: Now, how do you think we can pass on this information of the new method when you are the one who tests for HIV to others and more so the men?

R: In other people? We have to use radios like Bukedde, even mobilizations like here in Kayabwe and the pass on an advertisement, they come here, and they start teaching them yes it helps.

M: What of you, if you can take the information because some of you have understood, and some of you have not understood, you are the ones that have represented the ones who have not been present.

R: I get one of my friends and I explain to him very well. For me I suggest if you get money and you organize a concert here and you bring artists here because if a person can see those artists, that person will just come by and say eh and they tell that person that this promotion is for AIDS but they have something they give you and you go and test for HIV, so you can be there when you are many provided that there is music.

You can say that I teach my friend and I teach that friend of mine poorly and I don’t explain that person very well let me say that they don’t explain to him very well and he can look like a the one in wrong yet I am the one who is wrong.

M: It’s right you can see things but what you see is not that you explain to that person, they may use it wrongly.

R: Now does this man want to stone his friend?

M: What other things are talking about? For you how can we overcome the bad things in this method? That’s where we have been. Don’t fold those papers I have last given you there are some things that are going onto those papers. How can we overcome the bad things?

R: Girls come here, come. Sir is that for also reading.

M: People can we overcome the bad things in that method?

R: That method to implement it is through staging concerts. You organize and say that, we come and you explain on those issues and even how it works. But even if you explain to him that they test in different gums because for me I see gums here down because I was once in school.
