# Supplementary material for: Prevalence, associated factors and perspectives of HIV testing among men in Uganda
Source: PLoS One. 2020 Aug 7;15(8):e0237402. doi: 10.1371/journal.pone.0237402 (PMC7413494; doi:10.1371/journal.pone.0237402)
Supplement: S1 File — (ZIP) [file pone.0237402.s002.zip › manuscript data/FGD Men 2-Eng.docx]

**M:** now, am so glad for your hospitality, am Joan from Makerere University College of Health Sciences, we have an agenda of improving on the system of HIV/AIDS testing and we are working together with ministry of health. This agenda of improving on the testing of the HIV/AIDS, the reason for my coming is to gather ideas from gentlemen, as men what their expectations are in this arrangement. Now before we continue, I would request that we all introduce ourselves, each of could tell us one name that he would like to use in this meeting then we continue, we can start from this side,

**R:** am S

**R:** am C

**M:** you S

**R:** yeah, Am CS [laughs]

**R:** M

**R:** P

**R:** Z

**M:** let us be loud, so that we can hear

**R:** G

**R:** F

**R:** J

**R:** R

**M:** R, I request that we try to be loud so that we can be able….. [Hanging], because I was requesting that we come close so that we can get them well. Now our first question….. [Some noise], this issue of testing for HIV that has gathered us here today, you as gentlemen, what do you think about it?

**R:** [noise of moving vehicles] it has no harm at all, it is good because it help us to know our stand as men so that we can live in the society and be able to plan for our families, we plan for the country, so I think is not bad at all.

**R:** testing is good, because when you test and know your status it helps, if you’re positive it helps you to start treatment early, and if you negative it helps you to protect yourself from being infected with HIV, I see it is a good plan

**M:** what do others suggest?….

Pi, it is very good to test….

**M:** you as gentlemen….

**R:** when you test it gives you the idea of where to go and to improve on your plans

**R:** testing is not bad, it is very good to know the status of your life, even to know the status of your wife, but the challenge is when one of you, you may find that your wife is positive or when it is the man who is positive, sometimes it causes family to breakdown and the wife may end up leaving, or the man may suggest to end the marriage and we separate……

Pi, true, it may be when one is positive

**R:** now he will say I cannot afford to be with a positive person because one is negative, now he will decide to end the marriage yet you have been together for a long period, but it may be discovered that she negative and when you are positive or when you are negative and yet she is positive, when one of you is positive, so it one of the trouble causers

**M:** is there any other person with another suggestion on this matter?

**R:** sometimes when I look at the gentlemen am seated with here, for I am a youth, all these are elders they can be my parents, but the challenge we are facing, maybe they never faced it, but for us we are facing them….

P**M:** youths of what particular age group

**R:** around 25, 27, 26 and below, the ladies we date, when you suggest to her to go for HIV testing she swallows aspirin and we they test, the virus is not detected that is one, another one, youths of our age, it is difficult to find one who has got the idea of getting tested, how are you going to solve this? [Vehicle noise] because my fellow youths date ladies, they date and the following day she pays him a visit and automatically they will have sex, now where could he have tested her from it is similar to these married men that they will be serious, but for us youths, how are you going to encourage us, before even now they may date, but after three days they will have sex, you see…

[Noise of crying baby]

**M:** this is why we are getting these ideas to ensure there is some improvement

P**R:** you can tell your fellow youth that did you test with that lady, you dated last night and today you have had sex with her or did you use a condom either of the two

**M:** because the HIV is so common among people…

**R:** [appreciates, yeah]

**M:** but……

Pi, because everyone is a counselor to the each other better though when you tell them so fear they fear to be informed that they are positive, he may even commit suicide [phone rings]

**M:** I would to know [phone rings]……

**R:** let me ask, is it true as he mentioned that they use aspirin, it is true that when you swallow Panadol also, HIV will not be detected?

**M:** those are people’s minds

Pr[people’s, then laughs]

**M:** and they are the reasons why there is increased disease spread, which is why all the time there is need for modification, so that is the reason why……[left hanging]

Pi, that is the reason why I request her we go for testing, but if she takes the tabulate and the virus is not identified, then….[he laughs]

**R:** do you know that many youths do not know about HIV testing, I think about nine of every ten, when one meets the girl today the following day they have to sex, you can tell him about testing…

Pi, many just test using their eyes [then laughs]

**R:** what I think it should be law…..

Pi, I have ever gone to mild may, but I met beautiful ladies whom you can’t miss to date, but they were getting ARVs [laughs]

**R:** but this method of testing people alongside the roads, it can help people to go for testing….

Pi, and those ones do not delays us, it takes like 5 minutes just then you know your status….

**R:** so it helps to test and know your status whether you are negative….

**M:** it also bring me a question, you as gentlemen what do you think, when you see someone going for HIV testing what do think of them those men who go for HIV testing?

**R:** it means that person is not sure of his behaviors…

**R:** because you go there expecting bad news…

**R:** now for someone to go and test, you go with two hearts, because if you have loved over 4 to 5 women, you go for testing when you are scared, may be you were not sure of one’s status, maybe one women has signs and you have ever had sex with her so you also expect your life not to be healthy which drives you to go for testing and know your status, if you go test and they inform you that you are negative, you continue protecting yourself, sometimes youth don’t know how to use condoms, sometimes back they used to bring health talks, teaching us how to use condoms, many people, doctor for us we know them [someone laughs in the background]

**R:** me what I think people should be able to understand this disease, because me when I was schooling in Mengo, I used to board a taxi, but you could just look at someone in the taxi and know this person is suffering from HIV/AIDS, but now things have changes, so being an HIV patient, it doesn’t mean that you are going to die, o it doesn’t mean that they will discriminate you, so there should be ways of sensitizing the people that having HIV is not the end of life so that people can go willingly in the hospital….

**M:** do you think that people have forgotten that having HIV doesn’t mean death or it is one of the challenges faced…

**R:** some people need health education, you inform them that one plus one equals to two or if you tell them that one plus one equals three and they accept, but if you really educate this person that one plus one isn’t equals to three, while using good approaches, he can understand, but now everyone knows that when I get infected with HIV that is the end of my life

Pi, it is true…

**M:** let me ask, you as gentlemen, do you think some people out there, do they know exact information regarding HIV/AIDS, even the youth inclusive

**R:** it is like this, as you have asked that do they know…..

**M:** do you think they know….

**R:** now like me personally [someone laughs in the background] I cannot lie to you, what they used to talk sometime back, what our parents used to say that you can see someone suffering from HIV/AIDS when he is wasted, like when you are a driver if you have not had a serious accident you may not fear to drive speedy and you continue driving speedy until you witness a serious accident, it is the same story these days that there is a lot of medicines that you can get someone who is truly infected and you cannot know that he is really infected, and even though they tell you, you cannot believe it, even though they bet with a million, you cannot accept that she is infected

Pi, because the medicines are available…

**R:** because the medicines have helped them to falsely inform people, hope you get it, that is why some of us, I cannot leave here that I am going for HIV testing, even the time I went for testing is when I had gone for safe male circumcision….[**M:** laughs], so they said that if you know that, if you know that you are positive [all laughs in the background], we shall not circumcise you, so when the doctor drew some blood from us for the HIV testing, whoever was called upon to go to the theater then you could just know that you are negative, but me leaving here to go and stand there that I do blood testing, what will I be testing, and some people even though we are seated here like this, you cannot know…..

**M:** so it means that gentlemen for them they don’t want to test for HIV/AIDS

Pi, testing…..

**M:** do you want to test for HIV or not…

**R:** majority of we gentlemen we do not do HIV testing, because you are ever scared all the time, because if you are infected, there is no chance of getting cured, all the future plans that you had set, to me it is the end [someone laughs in the background], because I don’t expect that I will do it

**M:** now, how do gentlemen know that they are HIV positive, or I acquired the virus or I have not acquired it

**Pi:** because you cannot know whether you have been infected by HIV virus if you have not gone for testing, how you realize that you are infected, what they tell you is what you follow..

**R:** these days how you can know it, because treatment has changed, if you acquire diseases like malaria or others, if you go the health facility, they force you to test for HIV also, they will tell you we shall test for malaria and we shall also test you for HIV so we establish if you are positive, now for them they are testing all if you don’t want, they can even fail to work on and refer you to another facility where they can work on you, now that is how they get the chance of testing the gentlemen, but is very difficult to find one going by himself to go for HIV testing, especially if you have loved over 4 or 5 women, and especially if you loved one and you never see her again, but the time you see her she has wasted, [people laughs in the background] when she has totally changed [Pi, there you just know….] sometime they may be beyond 10 or six, you find her in another place and she is not on the same conditions that you meet her at first, she has faded,

Pi, even she has lost her hair

**R:** you start praying for yourself and then you say….. [Left hanging]

**R:** another thing that you can know at that a gentlemen has been infected by HIV virus, or that you have the disease is when you come to know that your former loved one has passed on, as you there someone informs you that she had died, so within your heart you realize that you also infected, but if none of my lovers have never died, then no….[all laughs]

Pi, that it is like this strange malaria,

**R:** another way that they get to know is when you impregnate a girl, for her she come back with her results and they indicate that she is negative, so if the wife is negative, then it implies am also negative [all laughs]

**M:** now let me ask…..

**R:** there are sometimes, if your wife is going for antenatal care, these government facilities, you have to go with your wife, unless your wife has lied that you don’t stay together….

**R:** that is true you have to accompany her as in those facilities of yours that you are talking about, but right now, for us we have personal doctors, so I just call and tell him my wife is coming there so do something, but if you tell me to go there, I may not get time to go there as you the situation at the place of long queues, so you have to over wait

Pi, it cost you a lot of time

**R:** a lot of time

Pi, the whole day you are there

**R:** so I just tell her please go and be worked upon then both of us going …..

Pi, but for me I think it is your advantage…..

**R:** for me let me go and work for food, it is advantageous, but I want you to look at it critically….

Pi, for sure the whole day….

**R:** this woman has gone and seated there as you said and you have also accompanied her and you seated there together and most likely that you may leave there when they have not worked on…

Pi, that thing has even happened on me….

**R:** so where will you get the money to feed people

**R:** may be you left home without any provisions

**R:** so many times when the ladies go alone, maybe he has told her to go and wait and for me I go and look for money

Pi, so for me I go and look for money…

**R:** but these things of us youth that you have to accompany the wife to the health facility, I have seen many of my friends, they even call me to go and help their wives, that come and drive her to the facility, you then take her but you become worried of the time you will have to see the doctor so you leave her there for a while and you just come back to pick her so those regulations of Kawempe that you suffered to accompany the wife….

**Pi:** those are false

**M:** what else prevent the gentlemen from going for HIV testing? we had heard those ones, but it seems there are many barriers that prevent the gentlemen from going for HIV testing, we had fear even this gentleman’s reason we have heard it, what else prevent the gentlemen from going for HIV testing?

**R:** there are those who are just not aware, or others when they don’t want to know, because if they tell him that he is positive

**M:** which category of people are not aware?

**R:** people don’t know what testing is, it is true that you may go and test for HIV, but I don’t see any reason to as why I should for testing when I don’t have it

**M:** because we first asked, how can you know

**R:** listen, for me unless they will just tell me or I will start seeing the signs that I am now positive in case I acquire it, but I cannot leave here walk straight to the health facility that I am going for HIV testing, I cannot [someone laughs], for that I cannot [Pi, it is impossible], seriously sworn I cannot

**Pi:** when you don’t have the virus,

**R:** yeah, why?

**R:** doctor me what I look at, in addition to the time wasted, I would expect that there is still need for awareness among people especially in public places where people gather mostly to continue informing people the advantages of this issue, otherwise if it doesn’t happen….. [left hanging] people may be wanting to go if they are encouraged maybe, but they are failed because there is no one encouraging them to go, so what I suggest is to have sensitization in public places and in other places where there are always many people, it may help that one who had not gone may be motivated to go

**M:** it has given me two questions that you as gentlemen if someone would like to improve in the issue of testing, what areas would you like to test from?

**R:** now the biggest problem now many people work in which particular areas, places like these markets, it needs to have service centers, in parks where people are moving to various places it requires them also to have, generally in places with many people

**R:** even in schools, especially these secondary schools and institutions….

**M:** so that we are able to improve, because we are targeting the improvement in among gentlemen, where can we arrange for gentlemen to go for testing?

**R:** yeah in the market, like yeah….

**M:** what if we are like outside this premise, like those areas far from Kampala, in that case where can we arrange for the gentlemen where they can test from because I have got the problem of delaying you in the health facilities it prevents you seriously…

**R:** as the other one said…..

**R:** there can be towns or centers…

**R:** majority of the men work on boda-boda, so it requires that even ion those big stages of boda-bodas

**M:** would you like a permanent area or just an abrupt place, so they just come instantly

Pi, it should be abrupt…

**R:** because even people are not tested every day, they test every day, even though they can come test for a week then they go, they test people for one week and they go because they will be testing the same people, they have been doing it though they have delayed, they used to come there we test, then after sometime they come back and still we test and they go to other places

Pi, they were there….

**M:** now let me ask, just a few days back, there was a new a method that had just been brought, it was more in the newspapers, the method of self-testing for HIV while using a strip that tests using saliva, did you hear of such news?, did you watch such news?

**R:** no, we never watched it…

**M:** there is anew method, and the ministry is looking for more improvements, this method you get a strip as we buy condoms now, you go and get it from the shop, let me assume that now everybody know how it is used, you go and use your condom and nobody should stop you even though you go to the shop, nobody should be criticizing you like what have you bought, it is like someone else who would buy a bread and moves, even this method is like that, someone will be able to go to the health facility buys this strip which tests using saliva, you rub it around the top and bottom gum then put it in some bottle after like 10-15 minutes you are able to know, you can decide to buy your only, or take home, such a method is there anybody who has ever heard about it

**R:** me what I see about that method, it is very dangerous, because many people if they know that they are HIV positive when they are alone, they may commit suicide, but there those experts who test, they have counselors [some murmuring in the background] at that very moment, that method is very dangerous, it will kill all people [some laughs in the background]

**M:** do you think you as gentlemen, can you choose to use that method of self-testing, because you find many problems

Pi, when you find that one is positive, they counsel you and they calm you down….

Pi, you even get courage from your fellows that you see…

Pi, even your wife is well informed, even you wife is called in kind, they ask her if they discover you are positive and the husband is negative, could you still stay with him or if it is revealed that the husband is positive or you are all positive will be able to stay together like that, if they counsel when you test positive then they inform you of your prescriptions, but there is a situation where they have tested you, they inform you that you are positive, then they leave you and they continue, you have known it within you that you are positive, but you have not got the medication, you reach in the hospital to start medication after knowing that your life has reduced, let me started on treatment, you reach there when the medicines are for selected people, they tell that the medicines are available though we need some fifty thousand shillings or thirty thousand….

**M:** now the aim this method is expected to be a starting point then you decide to go ahead, what others have to say…..

**R:** but for me method of using saliva

**M:** but now, when you are testing yourself….

**R:** while testing myself that I buy two stripes and another one I take it to test with my partner…

**M:** you decide yourself

**R:** with my wife…… [he laughs]

**M:** or whoever you have got, we are looking at improvement…

**R:** for me I see this method, the other old way is better according to me…

**R:** according to me it is good, because take it as if you have gone to the health facility they test you when you are positive, they first ask you will accept all the testing results, then you accept, so even when you test yourself when you negative or even your partner is negative or you are positive, you make suggestions….

**Pi:** they won’t you start that who was the cause…..

**R:** that will not come, because the disease…

**Pi:** then you start fighting and end up breaking….. [people laughs] when you are positive and the woman is negative are you not going to fight each other

**R:** it is the challenge [people laugh in the background] it needs when there is a counselor you cannot use that strip when there is no expert to counsel you

**M:** now if that method is brought [Pi, that is for the singles], because now we are looking at men, what do you suggest, what do you want to be done to ensure that if it is established when it is one of the ways you can utilize to know your HIV status, what would you like to accompany this method and what improvement they should put for you to ensure you utilize it if someone wishes

**R:** me I think it should optional, if one decides to use that method with the partner they should go ahead, but if not that then we should go back to the old method, like they should all be available….

**M:** yeah still they will be available

**R:** ok fine…

**M:** this one can be there just as we have the phones that you can access someone everywhere, but when you want to send something to Kenya you go to the buses, but you call and someone gets to know that you are sending something, if you decide that you have bought it then you go to the bus, now all these things come as improvement…

**R:** that method will help mostly with “on the spot” partners [all laughs in the background]

**Pi:** you just make a test when you get

**R:** you just find someone when you don’t know anything, she is new to the news they can use it

**Pi:** those with “on the spot” partners…….

**R:** you can use it the way you want, you go with it at home and tell your partner please first test [all laughs in the background], you may know that you are negative, they test you after two months, then you get someone, so it helps because you can buy the strip and tell her to first test, then you can be confident somehow…[all laughs]

**M:** now, which other category of people do you think that this method will be beneficial to? Because we want to see….

**R:** it is for the singles, those who people who decide for themselves alone, the man stays in his house alone, he may test and know his status, but if you are married, you may test, find that you are positive yet you are the one who bought it and the women is negative [people laughs]

Pi, she may even take it…..

**R:** that you have brought you disease [all laughs]

**R:** and first of all you should be trusting your partner but if you don’t trust her then you have to buy that strip and [left hanging] because you cannot tell, someone in a week can have over four women, now what do you expect from that

**R:** It can help the students very much

**R:** I think it helps because you stop testing using the eyes, testing that that one is negative

Pi, because some fear in the hospital, [laughs]

**M:** because I have heard it also that you testing using eyes…..

**R:** yeah, others [all laughs] he may move from hear and finds another and tells him that I found “him” going for the HIV testing

**R:** yeah, using eyes….

**R:** even some women have got it, they say that one has lost a wife, so…..

**M:** still also it is put there like condom we also go to the shop and buy them if someone has not moved with it then you give it to him so we want this method also to be like that, but we would like to know you as men what challenges may you face so that if there is any improvement it should be organized on the side of gentlemen, if there is such a method

**R:** that method then will work out, it is good

**Pi:** it is not bad….

**R:** because there are some prostitutes…..

**M:** now if it is established, in which areas that you would like to find these stripes to be used?

**R:** in the clinics

**R:** in the clinics

**R:** yeah as we said that you can go to the shops, so if they are establishing let be put in shops, those big ones….

**R:** in pharmacies

**R:** it is like you may go to the shop and ask for a condom and they give it to you, or just say give it to me [laughs]

**R:** you may have some other language that you can use, it like you going to the pharmacy to buy any other drugs,

**R:** you say give me that thing and I take it or aspirin [laughs]

**R:** because in the shops, there are those who may not be able to trade in it, though everything is just in the pharmacies, so you go and buy it from the pharmacy to go and it helps you out

**R:** and you buy it,

**R:** now just in a few minutes I was with a prostitute there she bought a pack of…., someone gave her condoms and they were like 20, she said only these ones are for two days [all laughs] and she even divided them and she was like these are for one night [all laughed] she says they are not enough…

**R:** she says you have brought materials and they are very expensive…., [all laughs] that was there when those things, the condoms, they used to be distributed though now days, but now someone came and gave it to her and she was like he has brought materials she divided them that these are for one night and these for the next night [then he laughed], they make use of them…

**R:** prostitutes protect themselves, though there are some difficult gentlemen who disturb them

**M:** I want you to speak louder…..

**R:** those stripes if they are to be brought in public to be utilized, they should consider so much the benefit of counselling in the health facility that if someone is going to test there is counselling, but what if I go buy it and I have no counselling then I test myself and find that I…..[left hanging], wont I commit suicide, before I even go to the one who brought it for either a women or me myself, counselling I think is very important, how I should behave, if I find myself positive what should I do, should I keep silent or I should also start spreading

**M:** now the aim is that when you know then you decide because even in the hospital maybe you have gone, then you decide whether you will go to the health facility though you go while knowing, so even though they are to repeat the test in the health facility, it will be just for the health worker to confirm because others say I fear they delay me at the health facility, so all these are coming to ensure improvement, now you as gentlemen, what would you like to be done, do you support that there should a method when one can have self-testing or it should remain at the health facility as it is today, when you could just go to the health facility only

**R:** another thing someone to do self-testing without being trained, it is not good, because some people are bad-hearted, after testing and knowing that they are positive, they start spreading, because they have not sensitized him that after testing you are supposed to do this and that, he will start spreading even before starting treatment

**M:** now let me ask,

**Pi:** these issue of testing whether am positive or not and even whether I go to the health facility or not, the personal believe, still you remain doing what you would have done even though you were to go to the health facility…

**R:** someone comes and say I cannot die alone, because I also acquired it through someone, but they will tell you if they test you positive, you are not supposed to spread it to other people

**M:** now let me ask what channels do you think can be used to teach people about this method if it is established, especially those that will reach the gentlemen, because you are now proposing for all men

**R:** me I see social media like Facebook,

**R:** radios only that these days some people are no longer listening, but the community radio is effective in teaching people, if the advert is given to then, they advertise it, because I have seen they have been advertising in these days….

**R:** even Televisions

**M:** now in this community where you are, if someone wants to pass on the information, say regarding health, which channel do they usually use, which channel can be use to ensure that at least I reach to about 8 in every 10 people

**R:** phones

**R:** phones,

**M:** Should they send messages or they make calls….

**R:** yeah, the message or they call….

**Pi:** some messages like those on whatsup,

**R:** but where will they get there numbers

Pi, even the MTN and Airtel, they always send messages on phone whatever category that they want,

**R:** but if someone comes here in the morning, they give out flyers to every person as we see the Pentecostals on the roads, they give to everyone passing by [Pi, they are flyers]

**M:** so should we assume that everyone can read and understand because it is the same issue with the phone message, because now we are generating ideas on the particular channel that may be utilized to ensure that if this methods is brought, [Pi, me I think….] at least the information comes first then followed by the testing-stripes

**R:** now you see, to write a letter to someone and reads it, gets it and reads, he doesn’t understand the same way if you find him and explain to him

**Pi:** when you give him the flyer he takes to another person, if the other person reads it and he informs him that this about testing

**R:** I even see people who give out the flyers, but some don’t know how to read English, somebody will tell you I don’t understand the English used here and another one will tell the Luganda used here I don’t understand, so….

**Pi:** we are not Baganda all, [he laughs]

**M:** can we develop it in at least two languages..

**Pi:** two languages,

**R:** not only two, even Swahili, but men understand Swahili….

**M:** yes sir what is your suggestion,

**R:** we can still use phones in form of caller tunes, let us say if they call you, it will be the first message to come, or when you call then that message comes [**M:** then you listens to it] [Pi, as you hear caller tunes], that method is better because for the message I may be busy and I postpone reading

**M:** do you think this method can also help people who are distant from Kampala

**R:** it is very possible to help them because the percentage of people having phones is now big and especially among the mature ones,

**M:** let me ask some another question, it is also about the same issue, you as gentlemen generally in a day or week how many hours do you spend at home

**R:** most of the time I spend it at work…..[someone laughs]

**M:** if someone would like to arrange for you or to reach you with these testing-stripes, may they are to be distribute, if someone take them home, can you be found at home?,

**R:** no [Pi, no]

**M:** now where can we get the gentlemen?….

**R:** at the workplaces..

**R:** at their workplaces….

**R:** at their respective jobs

**R:** now if you find us where we work from

**Pi:** phones are very important, communication, good communication is very crucial on whatsup, by the way if you can develop some caller tunes, they will be very effective, so everyone when he calls, it is the first thing to come, it becomes so encouraging, still don’t ignore the churches, have programs on health awareness, even in schools, if it becomes like a learning subject regarding that idea, it will be very helpful,

**R:** because even those who go around while advertising things, because many people gather as they always have music, even the message can be passed through that, as for them they only advertise what they sell, and they are ever mobile today they are in here tomorrow in Bwaise, they can be in Mulago, Nakulabye, still there will be some people who will learn from them

**Pi:** because for us they came there at Makerere

**M:** now if someone would like to get gentlemen and reach them with information like this one, he should to what particular places, you have told me of your respective jobs but you have not elaborated to me the different types of jobs like maybe for us we work from here other work from such and such places, because now….

**R:** yes, in the markets, arcades,

**R:** most places like markets, parks, the old and new taxi parks, men are always there, boda-boda stages men are always there, but if you say that you say you go home….[left hanging]

**M:** what about in these places distant from Kampala?

**R:** there are towns,

**R:** in places distant from Kampala, they have their main towns, and someone may plan and say I will visit the market, like the auction, and such places men are common there as one would simply say let me go to the market place and buy some fish, I will buy this….

**M:** now what I am looking at, what is it sir?…..

**R:** in the villages what I see, these radios should not be abandoned, men always want to move alongside with their radios, even when they are going to the gardens, there should be at least some programs in the pick hours, now like at this time where do you they are right now, they are in the gardens, in the morning at around 8, 9 and 10am they are in the gardens, then they are back, they are awaiting for the news, then such a message comes first, such a method can be very effective

**R:** now for my case, I see we should not separate these channels, they should be if something has come to benefit the Ugandans, all channels should be used like the phones, radios, televisions, because there are some people who only mind about listening to the radio and they don’t mind about the television or even he doesn’t have it, and there those who only mind about the television and they have no time for the radios, and there those who can tell you that I take long without listening to the radio, and there those who can say they said such a thing over the radio and he will say I never heard it, and others…….[Pi, others are ever on Facebook] and the other challenge of phones is that some of them don’t possess phones, or it got lost and he has not yet replaced it, because not everyone has got a phone, [Pi, you should not touch it….], now if he doesn’t have the phone, how will he access that information, which means he has to use a radio or television, there are other people who have neither the radio nor a television, so if there are some groups that could distribute the information in villages, especially in those villages because for us in town it is easy to access information, but in the villages everyone minds about his own business [Pi, especially on the landing sites] he has gone to the garden sometimes he has gone to look for food, all those [Pi, he just gets it abrupt that they even test HIV] so you may find that someone ends the whole day without access any information

**R:** on the news over the radio or television, because of the responsibilities they have they don’t mind about it, especially us the youth, most of the time many are on Facebook, whatsup, they really do not mind about the developmental issues if I see, and they are ever following the bad information [someone laughs], so I don’t know how you are going to sensitize this to ensure that it sinks in people’s minds [**M:** on their minds], then the second I have seen, because we are on the agenda of fighting the disease, not so, [**M:** yes it is], what I see before we even proceed further what has brought the over spread of these disease, even poverty, extreme poverty has caused poor conditions among people, a child let us say for you are a female child, and you are my sister but people have a mentality, you can find a girl of about 17, 19 or 20 years and she is with a man of over 50 years or 40, because he has money, for she has not considered her life, they only mind about wealth, it has also caused…..[left hanging] so consider so much the school-going children, the university students after graduation the person falls sick, they parents remain in distress, they have been suffering with tuition, they were expecting the child to help them in their old ages, maybe they have sold their properties, to support the child and brighten her future, but exactly after graduation she dies, because many old men who have some money they are dating young girls and because they also want money, she sees that the parents don’t have enough money yet she needs some essentials, it has also spread the diseases another thing is unemployment among people, because if someone has got a job that keeps him busy, you don’t over think about leisure, your minds are ever at work, but whenever you get ten thousand, they get the peers and they end up getting drunk and having sex, may be takes like 2 to 3 bottles of beer after they have sex with women because they see it as the end of the world, he has worked and there is no progress, there is increased poverty and problems, such condition may force them to have sex with a person who is not right and those are the things if the government is to effectively fight against HIV/AIDS, before they think of eliminating poverty, I think it will be difficult to end HIV, because they are the cause of poor conditions, children, look at the girl children they are dating old men but because of money, the youth are seen even advertising themselves on televisions, that they want wealthy women, which means if she is positive, they are not minding about their health they only need wealth, you see what takes him in such conditions

**Pi:** they love, around 18, they love those of 75, and for them they are after [laughs]

**R:** so since you are there, if you forward this information to the government consider those factors

Pi, that the youths are unemployed, there is increased poverty, so you find someone at the extreme end with no hope, in the village you see the conditions, even here in town you see the conditions, people have no option, if they can hide somewhere and someone can buy food for her the person can offer one hundred or two hundred thousand shillings,

**R:** we have a woman, she married to her grandson, but they were confident when [laughs]

**R:** the second thing I see is discipline, you see discipline is very important from the parents, long ago, our parents used to train us discipline, but if you just do what you want and grow without any guidance from the parents, no guidance in schools, the government imposed a lot of regulations and they are favoring the children so much, those young children the women, now even though you are a husband at home, you have less responsibility about your child or wife, if you say something or you have punished for wrong doing and never to do it again, they report you to police so next time if the child repeats the same mistake, you just ignore regardless of whether it is the right or wrong route that the child is taking, you consider the outcome if you are to punish. Discipline also, like here the Buganda government has tried to inform people about it, though people confuse it with politics and say someone has said this and they don’t consider the benefit in it, what has someone talked, because if people become disciplined, and have morals it can help in eliminating some diseases, because if you realize that you are infected, if you have that human behavior you cannot go and infect somebody’s child or let me go and infect someone’s wife, such things, the government should also consider them for me I think that they could be one of the ways that we can use to fight against this disease

**R:** even another thing, here in Makerere they have a vehicle, they do safe male circumcision, but that vehicle has helped in informing people, it moves around informing people that circumcision is free at Makerere, the men go and be circumcised, if such a thing is introduced and they go around advertising like that, as that vehicle of Makerere..

**Pi:** now like those girls, they met us at Bakuli, they requested us to go and get circumcised, we thought it was a joke and proposed that we shall come at a particular day, we mobilized ourselves, we were like 5 youths, some were of those we went with got scared and they went back, but when they saw us coming back with no effect, they also came and got circumcised,

**R:** now if they continue making such adverts, it can be reaching people because if the vehicle passes around, everyone here will informed and then they move ahead, that number reached will be much; like now that you people there is a new method that has been introduced and this is how it works such teaching methods are very good

**Pi:** in the villages….. [left hanging]

**R:** now just testing us, is there any hope that that virus will be fought and end in the population….

**M:** still there is more research, even those researching on drugs are doing more, now….

**R:** the reason why the HIV virus has persisted in Uganda…..

**M:** you see this gentleman’s ideas, the reason why this disease is complicated,

**Pi:** there are those who benefit from it

**M:** the disease is on us personally, it is due to personal behaviors and they are different from each other everyone has his own heart that is why we are proposing all these methods to ensure that people make decisions, it is an individual decision, even though we impose all these, it you to take decision

**R:** those experts, the professors, why is this disease not ending

**Pi:** after over 20 years, why is this disease not ending, except one thing, they should make for us a medicine to see that the medicine cures people [someone laughs in the background]

**M:** now I wanted to ask one question as we come to the end of the session, you as gentlemen if someone tests and he finds himself positive, how can someone help you to ensure that you are able to go and get medication and still comply on going to the get it so as to prolong life, because majority after you don’t go,

**R:** doctor the medicine of Uganda, doctor

**Pi:** the prolonging of life when am sick, it will not comfort me, ask me why; because I may be having hopes of getting more children like 3, in the future and they live and I may be having only one or two children, but I have been discovered yet I produced the other children when am negative, but I still have hope of producing more three children or four but they have informed me that I am positive, now even [all people laugh] am to produce the other four children yet I know that am positive, I just know even those children are positive, I just know that I have made a loss, they are the ones to follow me on death……

**Pi:** sir positive doesn’t stop you from producing other children

**Pi:** because when the woman gets pregnant, we have a program called MTC I think, it helps the child in the womb not to be infected and if your wife is to give birth and she is positive, she has to give birth from the health facility, so that the health workers can prevent the child from being infected and the child is produced when negative

**R:** now that is what telling you that you should go together with your wife and get tested, but for you have decided that you have no time to go for testing, you wife is either positive and you are negative…,

**Pi:** so if you are still planning to produce, you have to calculate, if you are positive and you don’t want to produce stop spreading to others, [someone laughs]

**R:** if it is on the card that you are negative, in the hospital they are scared, they may even fail to work on her if she was not tested, even if the health worker tests her and forgets to write on the card, she gets disturbed in the health facility, because they will be scared wondering if she is negative or positive, it has ever happened on me in Mulago

**M:** is there any other with another suggestion or a question on this concept before we come to the end, I have asked mine…

**R:** maybe my concern, you asked of how we can spread this information, another thing there are youths in the [**M:** speak louder so that they can all hear you], I mean especially in the villages, they gather in trading centers, there are big towns others gather at football pitches, other are in auctions/markets if possible that you can organize when knowing that a particular market operates on this particular day, you can go and camp there for that day and educate some people or providing flyers, you give out and they read

**Pi:** you also move around the homes, some you find at home seated

**R:** doctor may be my other concern is…….[left hanging]

M like at what time if you are to move around

**R:** like at around 1pm even the farmer will be ready home, you find them seated

**R:** doctor the other thing that I had forgotten, there used to be something when I used to be in the village those diseases….[left hanging], they were teaching people they were putting some sayings though I could not understand the meaning, but they were teaching people, they could suggest a day and say we shall spend three days in this particular town and they were teaching, but those things are no longer existing that we shall go to this particular town and we teach this particular issue, they have abandoned that method it used to be there sometime back, people could go around thinking about the teaching, so people understands as time goes on…..

**R:** Even in microfinances, there are some people who have never got education but they may come because of money, maybe if you could be going there and send a representative and he teaches, let say like pride they sit from 9am to 4pm now that person if they could dedicate like 5 minutes to him

**M:** for the case of money people can be patient

**R:** [laughs]

**R:** we are patient as we wait for money, all those are methods trying to…..

**R:** maybe another thing that I suggest, sending this message in two languages also, it is good but I think you should improve on it, because here in Uganda we have many tribes, there are those who only understand their mother tongue, when he knows only that, so if you put this message in English or Luganda, he will not understand it. So I suggest that you would have an organization then you know that in a particular district say Masaka, what language they speak; Luganda then you put that message in Luganda

**M:** Luganda

Pi, depending on the tribes, someone was saying he represents Lugbara, but he didn’t speak in Lugbara [laughs]

**R:** but know, if say in Mbarara what language they speak, Runyankole, then you send that message in Runyankole, let say in the north, what language they speak, either Acholi or Langi, because that language is easy as it is a mother tongue, even though you are a young child , you can understand it…

**M:** understand….

**R:** than English, as many are not educated

**M:** an implication even the direction for use on the stripes, they should be put in those languages….

**R:** yeah,

**R:** that is why even on the phone they give us a number of languages that if you want this language press two, yeah….

**R:** for I think it will be easy for someone, if every district we know the most used language there, say Lusoga, like in Jinja I know it is Lusoga, it eases to gather people and explain to them

**R:** there even the counsellor who will be there should be a Musoga

**R:** maybe another thing also, why don’t you go back to the old first method?, I think it was TASO that first used it, they were giving some incentives to those people they had tested that could motivate, there was some flou**R:** they were which type even, [Pi, they were yellow], yeah, so when they could test you positive, then they could give it to you,

**R:** there was even something they could give powdered milk….[people laugh]

**R:** then also use the method of these people who gather blood for donations [**M:** uuum], those of Nakasero Blood Bank, they provide some sodas [Pi, they also give biscuits], so not all that they donate blood because they are poor they just say that I will just go and eat, they accept and donate, so those incentives can also help in ….

**M:** improving

**R:** improving…

**M:** you have given me very good ideas…..

**R:** now, there is a friend of mine who came, then he told me that do you know I have been tested, but can you imagine after testing the doctor gave me ten thousand [people laughs], because he was positive, they put him in an ambulance, they tested them from Kitebi and they brought them to Mulago and started on treatment, then they gave them ten thousand shillings, so for him he came back praising that, [all people laugh]

**R:** me I see it is a good method and it would help us, like we are here in the market, as some time back there is somewhere we used to work from there were peer educators, they used to come and teach and they were stationed there, they were part of the workers, as for us here we may mobilize…..

Pi, you were…..

**R:** I was also part of that, we used to come to Wandegeya for training and we could spend like two weeks, so we could come, someone gets time and teaches, but he is a fellow worker…

**M:** but when he is your fellow worker

**R:** he starts and move around…

**M:** so do you think such a person can help especially amongst you gentlemen?

**R:** someone if interested…..

**M:** [uuuum]

**R:** now like you see the youth seated there, but someone is there to bring such stories

**M:** now what do you think are characteristics of the peer educators, they are used to like how, which they do they do, and how do they do it

**R:** they always teach people as I have told you, as you have come here to teach us…..

**R:** when they had gone for some training…..

**R:** they first got some training, then they also come and start teaching others at their workplaces

**R:** now for me like at Bakuli stage, I am responsible for visiting the patients

**M:** an implication if someone sends you a fellow gentleman as a peer educator it is easy you…. [people laughs]

**R:** it depends

**R:** now if someone just come, he may not dedicate that time, but for me if I among them it becomes easy for me to hear even their views and I also advise accordingly, like if you do this, you get the medication in time and follow the prescriptions, you stay alive and you even accomplish your future tasks

**M:** implying if someone could even give you those testing-stripes or condoms, you can easily reach your fellows, so they can understand the agenda very well

**R:** yeah…

**R:** now even when were for training, they were giving us condoms and we were distributing them we had our storage so if anyone come requesting, then I could give, but we were having them because for me I was part of it…

**R:** I had a friend of mine, he told me please am not feeling well, so I asked him why, he said it is like I got infected, so I encouraged him if he thought he was infected, then he should go for testing and verify, but he was like, they would test me yes, though I fear but I feel life is not okay, so by the time someone took him to Mulago, they had nowhere to start from

**M:** but if there are some people like your fellows here, do you think they can accompany them and one is encouraged from the other…..

**R:** yeah, because even the other person told him that we should go and you get tested, and by taking him for testing…..[left hanging] when they reached home, what surprised him the woman knew her stand and she was on treatment and she is still alive, but the gentleman died and we buried him sometime back…

**M:** sorry, ok gentlemen, I am so grateful for the discussion, maybe before we end, if there is anyone with another question, let him ask and we stop because ii don’t want again to put your work at standstill

**R:** doctor for me this is my last word is, for the case of the disease people have it or we have it, but the thing is they should try and get medicines, that is it

**R:** another thing, health workers in the health facilities are very rude, they are rude, if you just go to the health facility as they say that treatment is for free, someone we look at you like you are nothing….

**R:** and you have to first pay something

**R:** leave alone paying, but you reach there when he is rude and he feels like he is just helping you [some laughs], so even that scares people

**M:** yet the government has provided it…

**R:** yeah, the health workers in the health facilities are very rude, doctor

**R:** me, doctor on this issues of drugs, the medication for HIV/AIDS, people you should not be lied to, it is for free and you get it, from wherever you are seeking medication from you get it and it is for free and in abundance

**R:** but there is a lot of bureaucracy

**R:** and even the patients don’t go for it,

**R:** when super chargers’ wife came….

**R:** you don’t go there to get the drugs, the reason being, first give time I explain, because for me I have been in those people now like four that I have been working with when they are positive, but I could tell them everything, some of them even reached the extent of burning his book, that I went and stayed long on the queues and I got tired, so he went for the second time and they refused to give him the drugs, so we went together because I had also gone there, so they told me whenever this man comes here, he doesn’t reach the serving point for the drugs, they asked for the book and he never had it, so they asked for 5000 for him to get the book, and I told him of the 5000 that they are requesting you, yet you don’t have the book and they will give it to you at that 5000, so he paid, and as per nor he gets his drugs very well. But there other fine was because he never had responsibility over his life, he had lost the book yet he is supposed to have it so that they keep recording his status and prescriptions, and days of going back to the health facility and get drugs, it is there in plenty, people are getting, yeah seriously

**M:** but my dears as we conclude, the disease is there, but the government is fighting hard to see that the disease is reduced….

**R:** it has not done anything….

**M:** that is why they are even trying out this [laughs]

**R:** [all struggling to say something], by the time HIV came, went it came, you could all die and you could even have rushes and scratch the whole body [**M:** I lost some relatives], even though you could find someone on road, you could just know that he is really positive, the person could only maintain the voice for that first HIV

**M:** but it was serious…….

**R:** but now people, you cannot know, they are even my size…

**R:** my dears, the HIV that killed my father was of that type, but they were calling witchcraft, many people died in Masaka and they could even demarcate with crosses, they could lock the house because all people had died…

**R:** but now somebody dies at your own size…..

**R:** you can sleep and waken up dead,

**R:** yeah [all died]

**R:** as it was capable of fighting that first instance, now there is a new one, now it should fight the next thing, that the first phase ended, now this one is around and we are seeing it, now they should fight and say the medicine,

**R:** he said that some medicine at Frieca pharmacy, is only affordable to those with money, but it is different, this Super-Charger who was working on radio Simba, they hosted him and he was telling people that you should stop testing using eyes, because you can see someone who looks poorly…

**M:** because even testing using eyes is not good

**R:** and you think she is positive yet she is the one who is negative [some laughs]

**R:** if you have your money, [**M:** this disease…..] you can go to the health facility and test your blood when it cannot fight against the disease that you may be having, so you may say let me go to South Africa and they can remove it, it is your money, if it is infected again you and get another

**M:** this disease is in the blood, we don’t test using eyes that is why we want to mobilize people to test, let us test, individually you know what you do, the channels that the government put in place for testing, so encourage you friends to test, so that we can improve on lives.

**R:** maybe another thing doctor there are those who say these things you put on the road, so someone can say, I don’t fear that is nothing, how can you come to an area and you fail to get any positive case, they test like 50 people and the machines says that they are all negative, how is that thing, still they discourage us that those testing sites on road, no, [laughs]

**M:** now you see, those are just talking, this disease as you said, as you see the eyes lie to us, HIV is not tested with the ears; listening to what people are saying, everyone it is his blood, even this method that I was explaining to you, that we have been discussing, we want everyone to be a secret, as we said here in the beginning, we are going to leave when no one knows each other’s name, so we want to keep the one’s secret, so it is the same with this disease, so no one can test you and later brings it out in public…

**R:** that he is here [all laughs]

**M:** that please I have found him because there are so many things as you have been sharing with me, that may wife, I will chase here, or me myself I will be going, so because of all those problems we don’t want that because one has known his status to be the end, but we want another method if you can use the drugs, take it, even if am positive, I should not be bedridden, I will do work even I will be going for journeys, I will support the children, even producing we have known that we can continue to produce if you have started treatment\. Gentlemen am so grateful for your time that you have dedicated to me, even the questions and the suggestions it was good, and I will forward all of them to ensure we improve the program

**R:** in the services….

**M:** to ensure that gentlemen continue to go for the programs like HIV testing

**R:** we are so grateful madam….

**R:** and those stripes to ensure that they help people to test and know whether negative or positive, but it can be a good beginning

**R:** they should also be available, because it takes like just 20 minutes

R it can also stimulate someone that let me go and do some test

**R:** they help to do test in hurry, like let me go here and do the test, after 20 minutes I will be through

**R:** 20 minutes are even much, just five….

**R:** ten minutes, yeah…

**R:** madam on behave of the gentlemen, we are so much delighted, you have done so nice to get our ideas, now our request that those ideas are implemented, but we hope that we shall be healthy..

**M:** it is true sir thank you so much
