# Supplementary material for: Prevalence, associated factors and perspectives of HIV testing among men in Uganda
Source: PLoS One. 2020 Aug 7;15(8):e0237402. doi: 10.1371/journal.pone.0237402 (PMC7413494; doi:10.1371/journal.pone.0237402)
Supplement: S1 File — (ZIP) [file pone.0237402.s002.zip › manuscript data/FGD Men 3-Eng.docx]

**M:** ok let us start, gentlemen I am pleased to meet, my name is Joan from Makerere university college of health sciences, and the aim of our meeting here today is to gather your ideas as gentlemen on the issue of testing for HIV, the ministry of health is looking at making some adjustments in the way these services are offered to men, so this should be built on your ideas and what you face, so before we continue I would request that we introduce ourselves so that we know each other you are free to introduce yourself with only one name today,

**R:** I am S

**R:** I am M

**M:** you will be a bit loud

**R:** I work from Wandegeya….

**M:** I glad to meet you sir

**R:** I am M

**R:** P

**R:** I am called C

**R:** I am called K

**R:** N

**R:** I am called B

**M:** now we shall be speaking loud enough so that the voices can be captured, now as we continue I would like to know, this issue, the reason for our gathering here, the issue of testing for HIV, you as gentlemen, the youth what do you think about it?

**R:** I would think that testing for HIV would help us in both sides, to know the status of your partner or even yourself to know your status, it helps to protect yourself and also to continue with your partner or to leave her so I think testing is not bad to know your status, for that reason madam it is not bad in any way to test for HIV

**M:** what about others, what do you think?…

**R:** My idea is, testing is not bad, but the challenge in testing, if I go for testing and then inform the wife, she starts a struggle, that I am the cause of the disease so I have realized that I am weakening then went for testing

**R:** the side of women, if they go to the health facility during antenatal and they are tested positive, if they come back home, they don’t reveal it, they don’t show you the testing results, that is where there is trouble, the other side if you say I will be honest and say reveal, it will be great struggle at home, she may even divorce and if the women goes to the health facility and she is tested positive, she doesn’t reveal to you, she keeps quiet, that is the challenge, that is what you should research, but testing is not bad….

**M:** what do the others have to say?

**R:** maybe my suggestion, I would think that testing is not bad, it is good you know your status and know your stand and also your partner you know her status, and also what disturbs us gentlemen in testing, the women can be pregnant, she goes to the health facility, they tell her that they will not work on not until the husband comes along, so we as gentlemen, it is disturbing, sometimes you are for work, so I would like that method to be changed somehow, if the women goes to the health facility for antenatal, they will not serve her if the husband is not there, so I would request that they simplify the system for us

**M:** what of the others, what are your suggestions?

**R:** my suggestion on the issue of testing, it wouldn’t be bad, testing is good but most of the time if you are married and tell you wife that your wife that you for testing, most of the time it creates a condition which is not good, the reason she will put forward is, that why do you go for testing yet all days we have been together it seems you got another person and now you are scared, so you cheated on her so most of the time if you are married, there is some struggle to go together to test and know the truth, so I would request that we all join and there should be no struggle so you go and know your status so that you can prolong your life, that is what I was suggesting

**M:** now from your contributions here, I got two questions, first I would to know, what challenges do you face, some you have mentioned a few challenges that you face regarding testing for HIV

**R:** sometimes there are cases you go for testing many times, I have a friend, he tested himself many times and they were telling him that he is negative, but after going to another place, the other first three test were all in one place, then when he went to another health facility then they told him that he was positive yet everyday they were telling him that he was negative, so I don’t know such a thing what are going to do to ensure that if you go for testing you get the correct results

**R:** in addition madam we have discovered it among our friends, if you have your partner if she tells you that we go to this particular health facility, sometimes by the time you go there, she has already bribed the health workers and they give you wrong results, so for you go when are confident with what they told you yet it is wrong, that issues I think we need some sensitization, so people should be strong and receive their true results….

**M:** and testing should not be after planning, if you decide to go, it should be abrupt, then you decide and go….

**R:** but still on that madam as I add on, there should be someone with a stronger need for testing, so that one with the strong mind on it is the one to tell madam, let us go for testing, so you find that the partner was not prepared, so one of you will get that feeling….

**M:** now you as gentlemen, how do you think you can lead in this issue of testing, what can you do to ensure that you lead it, to ensure that you go with these wives and test or even among yourself to mobilize yourself to go for testing

**R:** it is true to mobilize to go for testing, but the challenge is that you may get your partner and before having sex you decide and she tells you that we should go for testing so that we know each other’s status but as my fellow said, those are the challenges we face, you partner will first bribe where you are going to test from and it is where she will take you, so they will give you results that she is negative and you also negative yet she is positive, that is the challenge we have with the health workers who are bribed to destroy peoples’ lives, I don’t know how we are going to go about it….

**M:** that issue, do you have a specific area that it happens, or what are your common areas that you usually go to for testing, because sometimes it may be a particular area, that is why I am inquiring that you as gentlemen, where do you go for testing if you decide to go

**R:** in most cases, it is with these people who move in every zone, they are in ever zones in most cases, and you find that if the person who is positive is tested negative

**R:** me I think like this, the government should come up and select particular health facilities and inform people that these are the health facilities were you can get true results and people know it, otherwise people are being lied to, someone goes to a place expecting a different something and gets a different one, and the other issue that disturbs people most on the issue of testing, as you see we are seated here today, I may be remaining with only 20 minutes to leave, but you may go for testing and find a very long line and you have to sit there, yet you left children are demanding for money as you know our economy we are struggling to earn a living, so even that one I forward it to the government, it should find ways of helping on the issue say if someone comes here for testing, he will be given some money, though it is good for your health, but the time you spend there, the government has not considered your time your spend there as most of the people, we have responsibilities, at home you have people demanding for money, so when you calculate it well the hours you will spend there, though it is for your health but behind you, it is affecting you…..

M …affecting you

**R:** you can sit for 20 minutes and leave, because I have left children at home with no food,

**M:** and others what do you think…..

**R:** me, I have just a question, if someone takes septrine really and he is tested, is the virus not seen?

**M:** that issue I have heard it, it has been raised but……

**R:** because they say if you tell her that we go for testing, then she swallows 2-3 tablets, then you the machine doesn’t recognize the virus

**M:** I have heard some statements, but what I can tell you is that, most of the time if someone tests and find that the virus is not seen in her blood, there are two reasons; first it means that since she acquired the virus, because it takes some time that is why sometimes when you go to the health facility they tell you to go back after three months, you may test and they tell you, that you are negative because you acquired it last night, still you cannot expect to visible, but after three month, that is the duration that was suggested through research that at least it will be visible in the blood, so if someone has not been moving well and tests like four times in one week, still they will tell him that he is negative, but if after four months he goes to another facility, they will tell him that he is positive and the other issue that you may find someone that he is negative, if someone is aware, let say I was in Kyotera then I realize that I am positive and start on treatment from Kyotera, I take drugs, but after a while I leave that place and now come to Makindye, I get another job, but remember that I have been on treatment, you find me and say let us go for testing but I have been on treatment for a full year and I have been following the prescription, because the viral load has reduced, even though they will test, it may show that I am…..

**R:** negative

**M:** especially if I had been adherent to treatment, and remember this disease also differ according to individual bodies, it is there, so this problem remains on individuals, that you should be honest, it is the cause of that challenge, that is why we are looking for improvements to ensure we get solutions to such, what should be done, that is why I was going to ask you another question in addition, that now as you as I have heard that sometimes you go somewhere……, so now you as gentlemen if you decide today that I am going for testing, what channels do you have that you can use to for testing, which is not a hospital because you talked about it and stated its challenges, where else have been going for testing?

**R:** testing we always go to these people who gather in a place and set up equipment

**R:** in the clinics sometimes we go and test

**R:** the challenge we face is that you may test for only one time, and it is challenge to spend the whole year the three months they talk of, it is difficult to go back for the remaining two times after the three months

**M:** the consecutive ones…..

**R:** and me I see that the biggest population, most of the time they only test once, what he is given there is what he considers, me inclusive I won’t lie to you I have never tested for three times, like I tested and then required to go back after three month I go back, it is difficult, most of time for me where I see they have set up and there is testing, then I go that is the end

**M:** and others…

**R:** me I have gone there for three times at the Makerere building as you slope to Makerere, I have been there three times, the first time I went there in December the second time I went on 12^th^ April the next was in June…

**M:** you were so consistent which many people fail

**R:** and for us we find this……, as you asked that which side do we always……, these services sometimes we find them in roundabouts of roads, when they have setup tents and they are testing blood and sometimes they come here at the market were we work from and they say they are testing blood, in fact you may find that there were we go in the health facilities you may witness that they pick it and place it somewhere, but these people we find in roads, in fact though they test, sometimes people are not satisfied, as they will just test and tell you that you are negative, I have never seen a positive one [all laughs]

**R:** it is true

**R:** what I was trying to say….

**R:** even the positive one they say they are negative

**R:** if one sees you at the testing site, he considers you to be positive, because those you find there……

**M:** the implication you don’t want…….

**R:** you cannot got there, still we should just test with these ones here in clinic like that….

**M:** now let me ask this…….

**R:** but there, they are here ask them

**M:** let me ask the question, do you have a suggestion…..

**R:** I was also asking a question

**M:** ok, ask you question….

**R:** what am asking, there those I hearing saying if you have sex with a positive person and then you hurry before elapsing of what minutes……

**R:** 24 hours

**R:** yeah 24 hours, that there is medicine you can take that can prevent that virus, it either called PEP, or what, i don’t know….., so we want your clarification on that

**M:** as you have said it, it is true…..

**R:** it is true….

**M:** but [all laughs], but be kin with this ‘but’, my ‘but’ says, yes that medicine is there it helps but they cannot offer it to if you go the health facility when it was intentional, you decided that you are not going to test, that medicine was established to help those in trouble, it was an accident…..

**R:** you have been raped….

**M:** you have been raped, there was some situation that occurred, you were beaten, may be thieves, but the thief might have had crushes so in the process you are bleeding unknowingly, such circumstances, so it is for helping those in trouble, yes you have been infected but it was unintentional, and the others to be helped like say we went to a party. I drank alcohol and I didn’t understand what followed, so someone comes pleading, in the morning she cannot understand what happened, it was uncertainty, it is there to help such people, but not this one who says I am going to do……,

**R:** now…..

**M:** let me first explain to you this, because the very medicine they offer you to prevent is the very medicine used for treatment, if you decide to use the dosage of coatem that was a remaining for the child’s treatment because the child is now fine, now if the mosquitoes bite, what will you use for treatment, already you have put coatem in your body yet you have no infection, now if they bite you, implies you are to die and you how dangerous the malaria is now days, it very dangerous not like that of those days, so that is it, now that is why I was asking you as gentlemen, because you sometimes go to the health facilities and you get tested, what do you about the current method of blood testing which is available now days, if you consider the blood drawn from the finger and the one from here, do you think they are different or you trust them that whenever they draw it from what do you think about it

**R:** on that issue I would say like, the number of times that I have tested myself, this one drawn from the finger they get very little blood and it may let me think that it cannot reveal the virus than what they would draw from the vessel of the hands, as that one is a bit much, it can help to reveal the virus very fast.

**R:** now I was asking, that blood that is drawn from the finger after they prick me and put on the slide, can that contain the virus? [all laughs]

**M:** let us first get other members suggestions, others what do you think about it

**M:** me to, that one drawn from the finger I am not satisfied as I am satisfied with the one drawn from here, because some time back I went to Bamani, when it was just starting and my blood spent like two weeks, even that side where we donate blood from they tell us if you want to know your HIV status, come back at this particular date and you go back, but these people who draw it from the fingers,

**R:** am not satisfied with it, I like that one which deals for a week before getting the results, come back after two weeks and get your result, there what they tell me, I cannot doubt I just know what they have told me is correct

**M:** others what do you have to say about it

**R:** me I say this style of getting testing blood, were they prick here at the finger I just say they pretend to work [all laughs], they are fake

**R:** they are fake

**M:** but you know that it is all blood…..

**R:** what I want to ask……

**R:** it is all blood but……

**R:** someone draw some blood from my finger there at Makerere building, and I was asked, the remaining should we keep it or we leave it, I told her to just leave it for a reason, the amount of blood they had got was just one drop, so I was wondering how are they going to keep, that is the reason I said they should leave it, so for drawing blood from the finger to me it has no meaning, they should draw it from the hand…..

**M:** implying you here, you mostly want to draw much blood and then you will be satisfied…

**R:** yeah, you become satisfied

**R:** because that of the finger you cannot be sure, and me drawing blood from there are those who get your blood, then after like three minutes they tell you that you are negative, to me I leave uncontended

**R:** yet you know you are positive

**M:** now how did you come to realize because this disease is not tested with eyes…..

**R:** now depending on where you engaged, as you know well…. [some laughs]

**M:** it is not like that, this disease is not tested using eyes

**R:** in addition, with this much, they can even keep some, if you doubt you can go back and they test, and they will not have to prick you again….

**M:** now let me ask, the ministry of health of recent, there was some trending news, there is a new method of testing for HIV, but this time they use saliva, who heard about it, the stripe you rub it around the upper and lower gums, who heard about it….

**R:** it is true what you are saying, I have ever heard on the radio and it was published in the newspapers, but seriously I don’t think especially with my fellows here that there is anyone who has ever seen it, because I think they are still scarce among people, but they will be of great benefit if the ministry of health distributes them among people, that is what I would request the ministry of health, that they should make them popular in the communities, so that they can reach the poor also, not only to be for those on top….

**M:** and they are not only for top officials, that is why they established it to ensure they improve, so today we would like to know your ideas, on such a new method, what do you think of it…..

**R:** still I was requesting, those top people should tell us and not fool us around, they tell us that HIV is blood to blood, and now I hearing of saliva my dear they should help and explain to us about it [some laughs], how the saliva connects with the HIV, because they tell us that HIV is blood to blood, so how does the saliva come into the blood [all laughs]

**M:** we are going to discuss, you should remind me about it as we continue [all were laughing], yes M, what do you think of this method…

**R:** that method would be good, but they cannot circulate it in the entire public, because they are very few who knows about it…

**M:** again why, have you heard about that method….

**R:** I have never heard about it

**M:** you have never who else has ever heard about it,

**R:** I heard about……

**R:** I heard about

**M:** that is why I wanted to know your suggestions about it, because if this method comes, it is yourself to make the test……

**R:** I don’t like that method…..

**R:** except when they have taught me I and am satisfied

**R:** it shows you that you are positive…..

**R:** if it is all about saliva…….

**R:** where I cannot believe that method, there is a friend of mine, a health worker he used to test the ladies for HIV, but as I speak he is positive, he could test them before having sex with them [laughs], but now he is positive and he is on treatment, yet he used to test the ladies, he could use this stripes, you cut and draw some blood on it, then after like 30 minutes, he will be sure, but now he is positive, so if you tell me about that stripe using saliva, I am not satisfied and I cannot use it as me

**R:** what I have not yet known about that stripe, it is good you have told us about it, is it like a toothbrush that you will us it alone or after me using another person can use it again?, if it is mine alone, it will be good to help me, but if it is to be given to another person to use it after me, I will not be satisfied with it

**M:** now it is like this……

**R:** that stripe, does it work only once?, now I may go to the shop and get it after knowing that am going somewhere and I will see that am negative, now does it work only once and it is finished

**M:** it works only once, when you get it you rub it around the upper and lower gum, then after you put it in the bottle containing some liquid, within like 10-15 minutes you will be able to know, but the aim is, as maybe we started some time back let say you wanted to buy a cloth, you had to board up to Kampala, but now things have come closer even those days the condoms you could go to Mulago or where, but now even if you wanted, even at your neighborhood shops, you can buy it as any other person who would come and buy a bread and goes, you can also buy it and move smoothly [some laughs], so it is the same aim they want to extend it to the public so that you can get a beginning that personally I can do this to know, even you can buy in bulk and take for your friends so that they can also get to know, but today we would like your suggestion, what do you think of this method, when you are testing yourself

**R:** now it is like this, let us say you have bought even for her or when you married, maybe the woman has bought and even buys for the husband, the woman or man may use it then after knowing that she is positive and she fails to tell you

**M:** but you will be seeing it there, because the aim is that you have agreed before bringing it

**R:** she may use it herself after knowing that she is positive, then she throws it away, that mine got lost…. [laughs]

**M:** how, you just go back to the shop [all laughs], what others have to say

**R:** that method may have no harm at all, but the challenge is teaching it to public and people are satisfied with it, because it is also good when am the patient and am directly involved, I test myself and know my status…..

**M:** then you decide whether you go the health facility

**R:** the major issue is to be satisfied well, what am going to use, what effects it has to me or no dangers and are the results correct

**R:** on addition to that, this method what may lead it to be not good, among we people the intended users, you will go and get it, but you will not get a counsellor to explain to you as it has been, today before they give you the results, they first counsel and ask if you are found positive, what are you going to do, some say I poison myself, others say I commit suicide, but here you will die alone [people laughed]

**R:** by morning you are dead……

**R:** you will poison yourself or commit suicide, which is not good……

**R:** you can easily commit suicide…

**R:** yet there you would get some counselling, you are encouraged and you become strong

**M:** now I have two questions, first, if someone would like to deliver information, to pass on the information regarding this method, which channel should be used mostly, which is good in that if one uses that channel, the information will reach us and we learn how that method is used….

**R:** on that, I would think if they are not extended to LCs or zones, they should put such information on the radios

**M:** now we are looking at the gentlemen, you as gentlemen if one wants to reach you with this information, how can it reach you? because today I found you here……

**R:** the challenge is, even though it is to be on radio, by the time it is on, some of us we are not there, now it requires health talks…..

**M:** will be able to attend them?

**R:** yeah…..

**R:** now when you came, are we not here

**R:** in all it needs mobilization…..

**R:** still on radio…….

**R:** they can try using these small papers, and they distribute……

**M:** the flyers

**R:** so each go on to read about it

**M:** if one gets them……

**R:** not all of us know how to read, but the radio wouldn’t be bad, but as M stated, they should extend health talks to people, because we face challenges reason being they bring most of the methods and we are not taught there operation, so those who know how to use have an advantage over those who may not know

**M:** yeah it is true

**R:** so they make use of that because he is aware, yet for you don’t know, then he puts you in his trap….

**M:** true….

**R:** another one madam you see these rich people, they publish their business by making use of the time when people are eager and must either watch it or listen like the Agataliko Nfufu news, many people, many commit their time and watch agataliko nfufu, if you can put such session teaching about those stripes, we learn because possibly many people will be watching and they can inform those who missed, I would request that….

**M:** ok, and others what do you suggest…

**R:** I add on what he stated first, those health talks are good but they should be through LCs, because most of us work maybe up to Friday, so maybe every Sunday there is announcement throughout the LC, informing people that we shall have a session about this and this, so that people can participate….

**M:** now sir if one would like reach you with this information or these stripes, you as gentlemen, if he has not met you here, where else can you be met ?

**R:** in the village….[all laugh]

**R:** that is what I have told you

**M:** where….

**R:** in the village….

**R:** LCs as I told you

**M:** which day and what time, because most of the time you said……

**R:** I told you that weekend, maybe a Sunday, you mobilize through the LCs, and people are informed….

**M:** at what time

**R:** in the evening like at 4pm…..

**M:** when do stay at home, let us first discuss that……

**R:** in the evening….

**M:** evening at what particular time

**R:** at around 4pm

**R:** at around 3pm 4pm and beyond

**M:** even people who are distant from Kampala, should we imagine that it is the same

**R:** Madam let me tell you, for a man to be brought to the LC, it is not easy [one laughs]

**M:** but you are the ones suggesting that

**R:** sessions as you see here, they can be organized in the community, even in town like that……

**R:** now madam we go…….

**M:** wait for awhile

**R:** but if you are to say people to go the LCs, how people will go there

**R:** many times, here there are sessions, this is not the first, but we always have sessions on other issues, they always mobilize with speakers, and not only here, they maybe even on zones that we shall be there on a particular day and time, we shall have a session regarding a particular matter especially like the evening time when people are from work, they can move around, people tend to remember such days even that time that they have decided, they always comply, or those stripes, should be brought to the villages, that is what I think

**M:** I wanted still to be elaborated to, what challenges do you foresee if this method is introduced, what challenges do you anticipate except what you have told me, the few, because you have told me a few that you need to be taught, what other challenges do you anticipate if it is introduced, do you think it will help you as gentlemen or you foresee challenges, we would like to know that also….

**R:** that method madam I foresee, it is going to make us loose two times, we shall not be knowing what we are going to do that is one, second we shall not get counsellors to teach us on how to handle our lives……

**M:** actually I had a question on that…..

**R:** when we are found positive

**M:** I had a question on that, if you are given this stripe, when it has instructions of use and how to operate it, do you think it will be enough…..

**R:** it is not enough because, now Madam, seriously something I have just read, it is very difficult for it to sink in the brain than what you have explained to me, because you may explain as you touch me, so you may find that you get the morale and……

**M:** you become strong

**R:** you become strong and do not do what you would have done, and am strengthened by your words, but what I read, they will not answer me anything, that is my suggestion, so….

**M:** and others what are your suggestions

**R:** you have talked about that those things of just reading, most of the time, those condoms have directions for use but you can find that 10 people of 20, don’t know how to use them and they end up doing what they wish, so reading may not be effective, though it may work in other places, but people want to just be informed face to face, there are those when they get some message from a person, they even ask some questions and they can be answered, but if I ask the paper what will it answer me, I may have my questions and I have nobody to answer me, but the paper you cannot ask it, it is just to read it and do accordingly [people laugh], have we understood each other……

**M:** yes sir

**R:** the second reason, this method is good yes, it cannot be with advantages only, but in the other method, the counsellor can tell you, you are not supposed to do this, you are not to do this if you are positive, but in this one, you know the gentlemen decisions, my wife if I find you with HIV go, whether we have children or not, when I found you positive just go, some even breakup from the hospital, yet there, there are people, what of here when we are just two [some laughs], after testing you that have you seen it, some may even, like me personally, by the time I bring it for test, I have my machete, if I find you positive I slaughter you, so it requires that people should be taught by counsellors. It is true everyone can afford this method, here someone can test and alone then hides it, but there the counsellor will first call, you know these are the results, but I don’t want you to do such and such, but here what comes into the mind is what you act, can wake up and decide to go and fall into the moving vehicle or decides to divorce, homes are going to separate and families will be no more, and child will take their decision and some people have sayings to the children, now you see what your mother did to me, yet some children are grown-up, now you see what you father infected me with HIV, the child will grow having that hatred for the father or mother

**M:** hating the mother or father yet if you are two on this current method, you are explained to and everyone will……, [left hanging], but there, there are some people who have no control, he will be blaming the other as the cause so for that reason, we need people to be taught, but when they are taught by individuals, by people close to them…..

**M:** physically…..

**R:** physically, many people have questions to ask, we have different understanding, they may be in Luganda yet am a Munyoro, I don’t understand Luganda well, because here in Kampala, we have so many tribes, if you say English, you may be able to read it, but explaining and understanding it, so you will read it truly but what is the meaning, so for those treasons, we need people to be teaching, maybe with time they can bring, because now you can find an adult person when he does not know how to use a condom now that yet they have grown-up when condoms are in existence, this is just coming, the government should help and extend health education down, here in the market we had MAVI, that was teaching people to prevent HIV, and LCs would work for us, though people we are not common on LCs, as me personally I may not be knowing the area LC chairperson yet I know the one in charge of my workplace

**M:** that is why I asked if one would like to find you in other places other than you work place, where can you be found, where else can you be found to reach you with this information

**R:** places where they maybe many people……

**M:** especially you as gentlemen, if one wants to meet you, where does he found you

**R:** these places below could be gathered in by gentlemen, they can be in places like at football but you will not get them to be taught such issues, at that moment, the program is about football

**M:** football…

**R:** because if you go to showing premise, the biggest population is of gentlemen, but again to introduce this, for you are not on business, they will ignore…..

**M:** that half time [all laughs]

**R:** yeah, they are eager of the half time, yet here at his workplace you can easily get him…..

**M:** now let me ask, I have just developed a thought, and can someone talk to you if he met you at betting place

**R:** it is impossible,

**R:** me I would think, there is no area you are going to find people when they are there idle, when they have nothing they are doing…..

**M:** I ask of gentlemen…..

**R:** but I would think like this, people at their workplace, it is the right place they should be met, because they just dedicate some time as we have done it, they come and sit in such sessions, those who maybe wishing, because they cannot all have the same wish….

**R:** sometime back I used to see the ministry of health vehicles with loud speakers, it could be parked somewhere, even though you were doing something, you get to hear of something touching you and you listen to it, by the time the vehicle leaves, you have picked it, even the Makerere vehicle always moves around mobilizing people for circumcision, many people have been circumcised from Makerere and I don’t think if you reach there they just grab you, still they first teach you about it and you consent by yourself for circumcision, that vehicle mobilizes many people, the vehicle my come and stops in the compound and they start passing on the message, all people at all level, top or bottom they all be listening, that we have brought a particular method and this is how it operates, and you will grab something let me go and ask for it from the shop or pharmacy, but still they teach you how to use it, that is what I think, the government should get many vehicles [phone rings]

**M:** so it moves around and explain about this method…..

**R:** yeah, like they move around advertising about voting, the vehicles are loud enough, and they are doing most of the adverts now, not on television, there are many televisions, but most of them are for Pentecostal, and most people are on such stations, and they have praying only, the news agataliko nfufufu, sometimes they come when you are not yet there and radios, there are many stations, they are no longer like the those days, I may be listening to super everyone has their own station of interest

**R:** but still many Ugandans, the biggest number are stubborn, according to me most of them are sick, that is why such things, they don’t mind much about it

**M:** now as we conclude, some of the changes that you want to be proposed have been heard. You want to be taught, what else you think that should be done to improve this method if it is introduced, as you answer me that one, I would also like to know, apart from you that I have met here, you think other people can also benefit from this method if it is introduced

**R:** yeah, it benefits them only that they don’t know

**M:** like which category that may benefit…..

**R:** gentlemen, most of the time the ladies, we gents are easy in testing, but the ladies are a challenge, you are very challenging the ladies…..[some laughs]

**M:** are you sure….

**R:** you don’t accept, you have many struggles

**R:** they will say, do you expect me to be infected

**R:** for us gents, you can be with you one wife without cheating, but ladies are easily tempted, she can get like 15 men in one day proposing to her but you can find that we gents after getting your wife, when you cannot date another woman, yet most of the time they are working, but ladies are tempted so much, even those things of testing,

**R:** they are even against it, why should they disturb me….

**R:** it is impossible the women cannot suggest that we go and test

**R:** they are so kin those ladies, most of the time…..

**R:** after they get pregnant

**R:** everything is at their home, medicines and others, she puts at the parents’ home, if she wants to take it, then she goes there, then she comes back for you will be thinking you have……..

**R:** right person

**R:** yet no [all laugh]

**R:** you are on the infected one

**R:** now there are some fellows, here in Kampala what I expect gentlemen, the biggest portion are boda-boda riders, so this method should be extended towards these boda-boda riders at the stages mostly where they stay, in the situation at hand, that the boda-boda man here will be knowing the fellow in Nakawa, will know the one in Bwaise and Kawempe, so in that instance if you come close to the boda-boda men, and usually they have no time, but they create time [phone rings], you can meet boda-boda gents when they are the ones listening to the gospel, the Pentecostal gospel, you can pass by the boda-boda men when they are watching the comedians, they are watching people fighting, when they are all gathered there but still there will be others which will be moving, but if those men are approached, I think door to door many stay on the stage and they spend there the whole day, so if one rides and goes when you were teaching, when he comes back the fellow can explain to him what happened, they easily inform each other as if one here informs the fellow in Nakawa, that there is such and such session, by the time you reach there, it will be easy, so they can get their time, yet to me I may not have it, you may come a time when I am going to collect money from debtors and they have sent me there and I get caught up, so those boda-boda gents, I think they should first be approached, now here the biggest number of gentlemen are boda-boda riders, so for that reason, these boda-boda gentlemen should be brought closer because they are a category I know who ignores much issues about testing, why should I test yet am married, so they ride away, for a lady I just date her once, the next I don’t mind, so you know the next thing, me a lady I don’t want to know, after two days, all the time, so we need to be taught we gentlemen especially, the sessions should be addressed to those gents, because they easily circulate the information

**M:** now if one first brings these heath talks, so you are taught then they bring these stripes, you think they can benefit you

**R:** they can beneficial that is why I told you that we need to be taught because for me I know that HIV is blood to blood, now if you tell me that that stripe uses saliva

**M:** I still have your question to answer [all laughs]

**R:** now if you tell me that the stripe uses saliva, seriously I don’t understand it completely, and in me, I still have that doubt

**R:** even fakes, in Uganda we have many fakes, you can have one which is original but when the other is fake, now what will you do?…..

**M:** to differentiate between the fake and the original…..

**R:** yeah the fake and the original, how to differentiate, because for you go there with your aim you have got your date and you want to test whether she is negative and you buy them two, now if you bring them but when they are fake, because they maybe similar…..

**M:** it is also very crucial issue that you have raised, is there any other person with another suggestion on this issue as we come to the end…..

**R:** to me I will not deceive you, that stripe, I got scared of it and you have to teach a lot [some laugh] to believe in it, because for me I got scared of I can’t deceive

**M:** this stripe is like a starting point, what it gives you, then you decide and either go to the health facility

**R:** now currently…..

**R:** you see for us gentlemen, I can be there with you when I have been eyeing on for a long time but when you don’t know what I feel, after me scrutinizing then I approach you….

**M:** within your heart….

**R:** and then I tell, if I propose and you accept, then I go to the pharmacy I come with my stripe [**M:** laughs], now I don’t take what you tell me, the stories, then I will no longer trust you, it is only the stripe that I have trusted, that is why I ask if it is fake, what should I do, as it will be finished

**M:** that is very important concern that we have to raise to ensure the ministry prevents it, I think my questions are getting done, except……

**R:** me I was thinking of something here when I was quiet, I think it may work as there are some stripes that you use as ladies, to test for pregnancy I want elaboration, do they confirm or not, if those one are right, even this one may work

**M:** they reveal [laughs] they seriously reveal, [all laugh], as I told you at the beginning things are a bit distant, but to help people to utilize something, you have to bring it closer that is why I asked, if somebody want to bring closer these testing stripes, where do you want to find them where can they place them and you know that I can pass there and get them

**R:** now madam on that, at least we got a chance wherever there is a station, now I talk in terms of condoms, they used to make us boxes in every station and they could place them there, sometimes we get them at the clinic sometimes they could just offer to us for free then after they were selling them so I thought that if they can take-up such methods, we can be picking them in the boxes, then we go and test, if they are not destroyed by heat, because you may put it there and if there is no building, it may be destroyed easily, but all that we can use them when we have learnt about them when we understand what they are supposed to do, as you see a condom there are those after opening it, they just grasped it they don’t even have to read the instruction, they just open and use,

**M:** do you think if you are taught how to use it, can you direct your friend, can you afford to teach it to your friend

**R:** it is very possible…..

**R:** it is possible I can teach it to my friend….

**R:** now it is like this, when those organizations came in those working in HIV like Uganda cares and another one, whenever they could come, they could bring many boxes of condoms, because even here in the market, they brought many, those who were taught and they went on giving to people, so even these stripes to know them they should go on and teach then after they offer them to us for free when we find them somewhere

**R:** but on my side I think that stripe should be sold expensively, because I see it as another disease [someone making a call in the background], even more than HIV, because it will lead to many death…..

**M:** how will it lead to those deaths…….

**R:** because after testing like this I may decide to commit suicide as he has suggested that they should be placed everywhere, so that stripe should be expensive on my side

**M:** and others what do you say about it, now is there anybody with a question, because mine are now done, except this gentleman…..

**R:** first answer him….

**M:** now let me explain, the HIV virus has not changed, it is still in blood, and it is blood to blood, I want to use similar words as he stated, but this is what happens, if the virus enters into the blood as you see everything produces antibodies, when it produces those antibodies, they are distributed in many parts, now in some parts the antibodies that come directly from the virus, where they are distributed is saliva, now this stripes tests the antibodies that are produced from the virus, implying if there are antibodies, then the virus are there, it is like you find flooding water implies there is a well, so as you see following water it cannot just come from nowhere [all laughs] it doesn’t mean that where there is water is where the lake is located, so it doesn’t mean that the virus is in the saliva, it is in blood, but while there the more it generates the antibodies that are distributed throughout, that is why it is not where they started from but it was later revealed that it generates antibodies and you can find them in the saliva, implying any person who would like to test but doesn’t want to be pricked, if you test the saliva and it contains the antibodies it means that you are positive and if the antibodies are not there then also the virus is not present

**R:** what are those ‘obusimu’

**M:** in English I can call them antibodies

**R:** antibodies….

**M:** which come directly from the virus, let say if I pealed, but you never saw peeling, how can you be sure that I peeled, if I show you my hands, you have to see the sap

**R:** the sap will be in the hands….

**M:** now that will show you that I peeled, even though I hid the knife and there is no food, I peeled from Kyakatebe, then I left and came to Kampala, you will automatically know that I peeled if I show you the hands, and you can either it was for the sweet potatoes, and or for the pumpkins, so even this is in the same way, if the virus is present it leaves some signs that it may not be there itself, but it sends a representative, so if you find that representative just know the virus is already present

**R:** at that very point, can saliva cause someone to be infected

**M:** saliva doesn’t cause one be infected…..

**R:** then why do we find the antibodies in the saliva…….

**M:** they are just antibodies from the virus, because the virus cannot survive in any other location except blood

**R:** doesn’t the government work with parliament?……[all laughs]

**M:** now what the virus feeds on is only found in blood, even where we started from they could tell us, that if they inject me using an a needle or let us start from the razorblade, if they cut me using a razorblade and I bleed on it, you should allow to be cut using it, they may use it on me in the morning and one throws it on the ground, then say it was a lot of blood, I have used a simple example let say I have given birth and a health worker works on, yet there is a lot of blood, then she works on me without using gloves yet she doesn’t know that she has wounds in the hands yet she has been exposed to blood, it is very easy if she has a wound because she has been handling blood, and it is blood to blood, she will be infected, but if you come in the evening and you are told to clean the room when the blood has dried, when it is just clots to be removed, implies everything in it has died but because it is dirt, it doesn’t require to use bare hands, if there is a needle where blood could have entered and within the blood has not yet dried, this virus its life is in blood, expect another thing which is not blood…..

**R:** I have my thinking……

**M:** where they stay is the fluid in the private parts, only that part, but here in saliva, it cannot live there, because even this saliva is alkaline, they work on the food you put in the month, they destroy everything, all that so there mechanism as saliva, it cannot survive there

**R:** whether my thinking is just weak and I have shallow understanding, as you said that this virus is good at being in blood, maybe in saliva it just urinates there [all laughed] now if you test like this then it just urinate on you…..

**M:** you may think like that as I have told you….

**R:** there is no virus, there is urine….

**M:** you have made me laugh, but in reality there is no virus in the saliva, but as you see where we started from if someone is infected, they don’t want even to share a plate, but it doesn’t affect you, because on a plate, there is no virus, that is what they were saying if you treatment be kin if you have wound, so even you it helps you to be kin, cause sometimes we get wounds unknowingly, and were you can think of virus if she has wounds in the mouth and then you use, maybe toothpick and it comes with some blood and you also put it in your mouth when it is still having the blood, but it should be blood, now that is it….., maybe if there is another one with another question

**R:** what I ask……

**M:** but it would be good we ask our question when they are part of our today’s topic

**R:** even in the sweat, can it be transmitted

**M:** no, it is not transmitted, now let me first pause for a while,

**From FGD Men 3b**

**R:** when someone can be able to get, it is also very good, it helps, 24 hours if you have not done it, for me I will be through

**M:** still this stripe is aimed at quickening, so you test yourself but you should be able to now

**R:** yes they say we cheat, but even I go for unknowingly that she is positive, it just get me accidently

**R:** sometime you go for someone when you don’t know, then those who see you getting out, they tell you that where you dated today you are infected, so you should get help [all laughed]

**R:** now that PEP, sometimes I don’t have money but if I go to Mulago, they will not serve me fast, they will put me in story and telling me they only offer it to those of accident, so I was just requesting that as they quicken those of accident, even as who are not of accident but I was just infected, they should also quicken for us, I should also be given that PEP

**M:** now you see these things, it is true they can give it to you, but what fails people to get it, it is one problem and it is from us people who go to get drugs, when talk about people who seek medication, because even me I may be talking with you today because I am health worker but if I acquire it I will also be like any other because am also human I also need someone to help me in that case….

**R:** you also go and pick it

**M:** the most important thing is that if some will help you, especially in terms of health, try to speak the truth, don’t lie, because if you speak the truth, I don’t doubt some even though you speak the truth and they cannot help you but most of the time if you come and talk the truth, someone cannot refuse to help you, you explain to the health worker truly they will help you, at least if it is just little which is available, they can offer half, to go and come back later

**R:** I also, in addition, someone may help me, but do you know that at this time our country is covered with corruption

**M:** that is true, but I want to emphasize on that issue……..

**R:** let me first finish, that you will reach in Mulago when you need that medicine, because I see, here even if they are offer us, they always advertise they put it in government health facilities….

**M:** of government…..

**R:** that I will go to Mulago when I need it, I personally I have ever been in Mulago, but the drugs you can get in Mulago, when they direct you to the dispensary to go and get it, it is only Panadol but the rest, they will direct you to and buy it from outside, but do you think it is not there or the government doesn’t supply it, still now if you are telling me to speak the truth, that is when corruption comes in, the health worker will tell me that I have now known the truth but…..[all laughs]

**R:** but what have done…..

**R:** he will tell you that I have known the truth, but what are you putting at hand, so have somehow that you should do it and we proceed, so I don’t know how the government is going to help us on such issues

M , those things are in existence, but today in Uganda any medicine regarding HIV, it is for free

**R:** for that I may be knowing it better than you…..

**M:** you are not supposed to pay for the medicine, even though it is PEP, it is for free, the reason why it is for free, that medicine is very expensive, so if someone if to pick from his pocket on a daily basis to buy that medicine, he will not be able to live longer but why do they make it for free, to prolong life, you may I don’t want to know those free things, but today, me and you and your relative and everyone who works, if you work and you have anything that you have ever bought when it is taxed, and I know everyone here pays the taxi in any one form just know you also pay for that medicine, so what we can do is to see that the number of those getting treatment reduces as for us we prevent ourselves from acquiring the disease so as to safe the taxes, I know some are embezzled as you know but even then we are still the ones to pay for the rest, because it doesn’t just come by any chance, tomorrow if the increase the price of one commodity from one hundred to two hundred shillings still know somewhere that at least twenty shillings will be sorted for that revenue and it will be for the drugs and still you are the one responsible for paying for the drugs, that is why they provide it for free…..

**R:** and this one…..,

**M:** now we are coming to an end and you are the last one

**R:** I wanted this one to be last and inform of a message, madam you are a beautiful lady and you look nice, but as we are here in learning session or even where you spend your time many test you using eyes, they don’t have testing equipment for HIV [all laughed], but on that I was conclude by requesting where you are delivering this information, that they should hurry to organize health education such that people should stop testing their fellows with eyes

**M:** very true…

**R:** it should be initiated in people, you are very beautiful lady [all laughed]

**M:** ok, am so grateful gentlemen for your time
