# Supplementary material for: Prevalence, associated factors and perspectives of HIV testing among men in Uganda
Source: PLoS One. 2020 Aug 7;15(8):e0237402. doi: 10.1371/journal.pone.0237402 (PMC7413494; doi:10.1371/journal.pone.0237402)
Supplement: S1 File — (ZIP) [file pone.0237402.s002.zip › manuscript data/FGD Men 4-Eng.docx]

**M:** now I have switched it on, ladies and gentlemen, am the lady, there are no other ladies [laughs] I welcome you once again, as I talked just a few minutes back, am AA am from Makerere University College of health sciences, and our aim today as we have gathered here is to get your ideas as gentlemen regarding the issue of testing for HIV [noise from the mosque], and maybe be we continue I request that we introduce ourselves and we know each other you are free to introduce yourself with one name that you wish to use in this meeting and we know that that is how you are called

**R:** I am Jo

**R:** I am Bu

**R:** I am Go

**R:** Mu

**R:** Ca

**R:** Fr

**R:** Mg

**M:** what….

**R:** Mg

**M:** am glad to meet you all, some names are complicated, whether you will repeat them I don’t know, but we shall move on, now the voices are need, but am going to request you as we are in a noisy place I am going to request you to be a bit loud so that we can be able to get the recordings [noise from the mosque]. Now from as we start I would like to know and as you also discuss, you as gentlemen, the issue of testing for HIV, what do you have to say about it, what do you think about it

**R:** according to me, testing for HIV isn’t bad as everyone will be able to know…..

**M:** let us be loud so that we can all hear you……

**R:** ok, so everyone to his status, so if there is any testing site, to me I wouldn’t oppose it in any way and I can also go and know my status whether I was negative or positive and you advise as health workers on what I should be doing next, or if am positive am supposed to do this so as to live longer

**M:** what do others say about testing for HIV?

**R:** testing wouldn’t be bad, it is very, very good, but there are those who fear to test, they fear not only me but even the rest…..

**M:** you as gentlemen….

**R:** yes, whom I cannot mention that so and so

**M:** still it doesn’t require to mention them…

**R:** as one will be amazed, one will say, I go for testing if positive even the one who had weight will reduce, others get stress, others are undecided, but it would be good to know your status as a gentleman….

**M:** what of other gentlemen, what do you suggest on the issue of testing?

**R:** testing wouldn’t be bad, it is very good but you cannot test alone yet you have a wife at home, to me I would think the couple should test together that is what I had…..

**M:** others do you have what to add on, the issue of testing for HIV, what do you have to say about it

**R:** I also second testing so that one knows his life status…..

**M:** ok, now as we continue, only that I have not heard from others, you as gentlemen, or any other man, or a youth, how many times should he test within a year let us start from there…

**R:** I think he should test at least three times

**M:** why do you think so….

**R:** because if you are testing after every three months or four and you go for testing to know your status, you may say I will test only once in a year but in the due course of year you may be dating other ladies and you may acquire it like that, but if you test like three time, you may confirm if even the most recent date was safe, yeah….

**M:** others what do you think?…..

**R:** as the former speaker proposed, even me I would give it months, at least after every three months, you should test yourself [noise of some music]

**M:** what about others, what is your suggestion?

**R:** it requires to test after four months, so after four months you are required to retest yourself, because I personally I test every week on Thursday, I have to test every Thursday… [noise of crying baby]

**M:** every week

**R:** every week [all laughed]

**M:** what motivates you to test every Thursday, because you need to share it with your fellow gentlemen [all laughed] I see them wondering….

**R:** there are some times when I see life is not good, so I go and test for everything, I test for hypertension, diabetes, ulcers, all, so me every Thursday, even yesterday I was at Abbey clinic, I have to know whether life is fine or not fine

**M:** now how do you make it, how do you tell your fellow gentlemen?

**R:** me I think every person should be testing every end of the week on Sunday, not waiting for moths, because you may wait and then acquire it within the due course, but when you test every week, you know whether life is……

**M:** now I have some two questions and we are going to discuss them those who go and test [crying baby], like every after week or three months as you have been discussing, what motivates you to go for testing , what do you think gives the gentlemen motivation to go and test…

**R:** where we explore, since we are gentlemen, some of us we are not sure, there is when you date someone and when you are not sure, though I have left safely but let me go and do some test to know my status, that is what makes us do that….

**M:** you had a suggestion, sir…..

**R:** me what I think, there is when not only testing for HIV/AIDS, there are other diseases like diabetes, hypertension and syphilis, to me, such diseases also need to be tested for because others think that HIV is the only disease, but yet other diseases can kill you, like hypertension and cancer

**R:** even cancer….

**R:** now like those ones, cancer and syphilis, they can also kill you…

**M:** but now today we are more emphasizing on the HIV/AIDS, what may drive you to decide to go for testing?, one gentleman here mentioned fear others to know their status, others what drives you, I have not yet got your ideas, what may drive you and you decide to go?

**R:** you have to go, as the conditions you go throw, you may be working from another place, yet you put up in another area, [crying baby] and know that we youths, we don’t have the same hearts, there are those who want to protect themselves and those who do not want to protect themselves, so maybe after last week, you would like to go and test to know if you acquired the virus, because the virus you may not know whether you acquired it or not, so that is why maybe after a week, you should go and test yourself

**M:** today it is like the people am having here are good at testing, but do you think even gentlemen outside there, do they also actively go for testing as our suggestions are today

**R:** there are others, they say, he has some common words he uses, I have a friend of mine who always say that “Was HIV intended for trees or humans?” [all laughed], now is there anything you can tell such a person, you cannot say anything, and God gifted him every lady he engages with, gets pregnant, he has nothing not even a building, he has nothing and yet he is aged, and he works, but the money he just uses it for leisure

**M:** now that mean…., what do other gentlemen out there think about testing for HIV or your fellow youths there?….

**R:** there, some gentlemen or youths, they fear to test to know whether they have the HIV virus, they say if I go to test and am positive, if someone gets to know it, how will it be, it is not a problem to go and test whether you are positive, it is not a problem now this one said that the virus was from trees [all laughed]

**R:** others say, will they be used to make timber? [all laughs]

**R:** yeah, will they be used to make timber so majority of us in Uganda, we are positively living with HIV, majority, we are sick, so we also need that method especially the youth, because the youth don’t want to test, even me I have ever tested but I can take more than three months minus testing again….

**M:** what do you think could be preventing the youth or gentlemen from testing, except the fear that he talked about, because you said that the youths don’t want to test, what do you think could be preventing them from testing?

**R:** mostly they say, they don’t want to know that may be am positive, so the results, how will they inform their friends that they are positive implying if he tells one friend, also the friend will tell another one friend and they will go on spreading that so and so is positive and everyone comes to know it…..

**M:** so issues of rumors prevents you so much…

**R:** yeah [people laughed]

**R:** it is true

**M:** what are some other obstacles that prevents the youths from testing?

**R:** what prevents them from going for the testing, not differing from what I first stated; some fear when one realizes that he is positive, he thinks that is the end of life, sometimes I hear over the radio that so and so after he was tested positive, he committed suicide, those are some of the reasons that prevents them from testing, but truly it would be good to know your status

**R:** testing wouldn’t be bad, but the challenge after they test you positive, there are no drugs, in Uganda the medicines for HIV is still little, even though they test you, you have to lineup, some even fear to test and know their status, they will be seen lining up for medicines, because it is for lining, and in Uganda the majority are positive, they are many, because if he goes on the line, he may find a friend, and they will be questioning him what have you come to do here [some laughs] and the medicines are few, but testing wouldn’t be a problem in the youths, but the medicines

**R:** but the HIV of today is not as strong as that one of those years…

**M:** but now, the issue of people on treatment seeking medication, do you think it is good or bad, if let us say that in every ten people you find there are 8 positive ones, now for that, I am looking at it in terms of seeking for treatment, so don’t you think it would help, it is like we all go to the shop, who doesn’t consume bread here, if you find me buying bread, is my problem yours

**R:** now see, in a country to have more cases of HIV, it is bad, because of one or two reasons, these days many old gentlemen date young girls so much, of which they would be dated by us the fellow young ones, yet they are the most common with HIV, in addition there is no any organization that will propose that they place marks on the HIV positive individuals that we may be able to identify them so in that case even though you have tested or not, you will die while….

**M:** die while seeing….

**R:** while seeing [some laughs]

**R:** but even the children of these days although he said that only gentlemen, even the boy children don’t want to work, you hear someone telling you, he is looking for a woman who has constructed, has enough money and she will only give him the job of just staying home, I always listen to them on Dembe FM in the night, at midnight, A young child of 16 years can call someone and ask; do you have a house?, are driving? [some laughs], very young just like 16 years, one of them I know he is my friend in Mukono, for us we wake up in the morning, come to Wandegeya and suffer I don’t have a decent job, but still I thank God for His Grace, I don’t beg, I don’t steal, but children don’t want to work, yet they want modern things, now he got a girl, actually a mature lady, she infected him then he went to their village, he felt sick that they were bewitching him after going to the village, then mother was even well aware but she got him someone’s daughter very young and attractive, they married within just one week, now the boy has died, we buried him last Sunday and the girl has gone to Busia while complaining that they bewitched her husband, even the orphan they had will be buried today

**M:** now look at such a situation, it is so worrying, now like in such a situation, if one wanted to mobilize gentlemen to test as the ministry is organising, what do you think that should be done to ensure that there are improvement and we encourage gentlemen to test, even the youths?

**R:** it should not only be among the male youths, even the girls, because the girls, they are so much in relationships

**R:** even the married gentlemen

**R:** the girls are more than the men, because a girl cannot have one boyfriend….

**R:** even the married wives…

**R:** she can have like 8 boyfriends and play around with them all, but if you go and test with your boyfriend or girlfriend and know your status, the good thing if you go for testing and you are a couple they will give you two people, if you see that she is positive and you are negative, then….., but the girls they are the worst, the girls,

**M:** now the ministry has an organization of bringing closer these issues of testing as you remember how we started sometime back that condoms were only found in health facilities, but now even in the nearby shops, even in the village, at least in some shop somewhere you can get it, even those for free are also available, now they plan to introduce a method that will bring these HIV testing kits closer to the people, if we are to start from there, do you think it will be good if they establish a method that will be closer to people and not in the health facilities this time, of testing for HIV…

**R:** let me first ask you one question, that method you are talking of that they will bring closer to people to test for HIV, as I asked you earlier how will I know that you are positive, so I avoid you, how I will know that you are positive and the other is negative, so I avoid this one and go on with another….

**M:** yeah, because you will be able test yourself, that is what I was explain to you

**R:** yes, I may test myself personally and know it but my friend may test and hide it from me

**M:** now the aim is like this, if we bring this method, you agree with your friend that you have even though your brother if he says that let us go and test today or your workmate and you decide to go and test today, if you have agreed you see…., but the aim in all this is to ensure that the way people take this issue changes, so that is the aim of all these changes, because those challenges, as I have told you we are looking for improvements, that is why I am asking, what do you think if there is a way in which one can test himself?, you test individually ….

**R:** and know..

**M:** and knows, what do you think about it, because many gentlemen in the health facilities, they are not common generally, what is your thought if they can establish such a method?….

**R:** the method of testing would not be bad…

**M:** when self-testing….

**R:** yeah, when am testing myself

**M:** yeah

**R:** then it is not bad, I cannot oppose it

**R:** to know as me individually, it is good, but, there is that issue, still I will not come and tell you that I am this status

**M:** implying that that is on you personally….

**R:** no, [**M:** laughs]

**R:** now let me ask…

**R:** first wait for a while, madam, let me tell you this one first, why I will not tell it to you, because even the one I have if she comes to know, she will also be quiet and these days, ladies and gentlemen, if one realizes that they will be going for testing, I have decided to go with the husband for testing, when going to the health facility, there are drugs that they first take, have you heard about it?, so when they test, they will not see the virus, yet she is positive, so me am asking is there any solution that can help to eliminate all those challenges, when you can know the true status of someone

**M:** that issue of drugs, you will bring it when coming to the end I will answer you, that question has been asked me for over the entire week, many times, others what do you think if they have established a method when you can do self-testing or you have your partner without now going to the public like health facilities

**R:** in your bedroom or your sitting room….

**R:** now if you test, how do you come to know…

**M:** now, a few days back, like 2-3 weeks, there is a method that the ministry of health published, the testing stripes, it tests using saliva, and you rub it on the upper and lower gum, then you place it in the bottle as you see these pregnancy testing stripes

**R:** yes

**M:** yeah, so that if you see two lines, then you know that I am pregnant, so even this one works in the same way, but it tests using saliva, one can purchase it if it is brought officially, one can go into the shop, whether you have decided to buy yours only, or yours and the brother or yours and the partner you take and test together how many have ever heard about that method, who heard about that news?, it was all over the radios, did you hear anything about it?, do you have something to say?, nothing….., who heard about that method?, what do you think of that method?, if they introduce to you a self-testing stripe for HIV when it uses saliva,

**R:** at least we rather remain to the old method, I go and get my injection and …[some laughs]

**M:** you go and get the injection

**R:** I may not like them what if they are not clean?, when they…., some may use them and place them back, how will I know that this has been used and this one has not been used?

**M:** it is only used once, it cannot be reused, it operates like these new model of the injection needles, have you ever seen them? when you push it and the medicines is over that is its end, what do you think if someone can set for you a method when you do self-testing using such a test, do you think it can help you gentlemen?….

**R:** now that if someone tests himself, he will not encourage himself to go for medication, he will just keep quiet, but if you go to the health facility, the health workers will counsel you and tell you what you should be doing, but if you test yourself, you just keep quiet, let me remaining doing such, now how knows about it, but in the health facility, they first counsel you that if you are positive, you are supposed to do this and you are not supposed to such, but if you do self-testing, how will you counsel yourself and even encourage yourself to go for treatment, you will just say let me remain here…

**M:** like I should die silently

**R:** another thing, I may deviate slightly from the agenda but not much, we love beautiful ladies so much, but those beautiful ladies are the ones who are most infected….

**M:** that is what you assume….

**R:** not assuming, sometimes it is the common thing

**M:** how will you confirm that she is positive?…

**R:** not how I got to know, but most of us gentlemen, we love mostly the beautiful ladies, you get me….

**M:** uuum

**R:** that is where we are infected from…

**M:** now how can someone help you, you as gentlemen to ensure that you come to know, how can you be helped to ensure that you come know, even yourself to know your status, than the way it is today, if they bring you such a method, do think it can be beneficial to you?

**R:** that method may workout, but Ugandan things, they may talk about and it takes like many years without being established, people are there, but we have challenges, here in Uganda we have so many cancer patients but we don’t have any machine to treat cancer but even that one we shall discuss and appreciate it but it will end here in words, and we shall be saying, we said such, but if we continue with the current method it is enough, you go with your partner get tested and get to know the status of each other

**M:** if someone would like to improve the current method, what changes would you like to see as gentlemen [noise from the mosque] to enable well to test for HIV?

**R:** today we have condoms, they are many, and they even offer them to us, but they say that the fluid within the condoms are also not good, they cause some diseases in men’s’ sperm ducts and the women’s tubes, I want to ask you is it true or false?

**M:** it depends on what you have used, some time back we got some challenge, they had supplied us with false condoms, but they were captured and they were all burnt, hope you watched it in news, sometime back, time has elapsed like two years….

**R:** because for me I have been hearing it from men….

**M:** but still condom we would talk about it, but today…., yes the condom is available and can prevent spread of HIV, but our agenda today is testing, because it is the only way you can know that you am positive or negative depending on the services we have today, how can one improve them to ensure that gentlemen are encouraged to test? [noise from the mosque], what can be done to ensure that it will be easy for you?

**R:** I want to ask, testing is not bad, but if you test and you are found positive, are the drugs available?

**M:** the drugs are there

**R:** then if it is available, then testing is not bad

**R:** you should test, you should test after every month, but while testing with your partner not the gentleman to test alone or the wife or your friend alone…..

**M:** so what can motivate your friend to come and test?, to come along with you…

**R:** now you have to agree…

**M:** do you think that can help all

**R:** you agree then you go for testing together but not going there alone

**R:** if you discuss and she refuses, then you leave her and get another person who will accept to go for testing, then why should I harden on you, I will be helping your health….

**R:** if you have a wife and say we go for testing and she refuse, then you just know she is positive

**R:** now if someone rapes and pregnant you daughter for him is positive and may poison himself, we always watch them on “agataliko nfufu”

**M:** now if someone wants to improve for the gentlemen, because that is the present gap, what can be done to ensure that we improve such that gentlemen can also be motivated to go for testing?, What would you personally like to be improved? [noise from vehicles]

**R:** I want to ask you, you have just gathered only gentlemen, and in your research did you find that there are more positive gents than the ladies…..

**M:** all people as I explained to you earlier this disease is for all people, ladies and gentlemen, but the people of interest, the ladies have their own interests and gentlemen also have their own interests, if you are going to make changes to anything, you can take what ladies suggested and then generalize it to gentlemen also, that is why today we would like to know, as gentlemen, what would they like to be improved?, so that they can go for testing and they know their status, as gentlemen, because women, there are also those who have talked to them to gather their suggestions, but now the suggestions of ladies is not similar to that of gentlemen

**R:** not that of gentlemen, it is not good to say that because ladies suggested, are you are a lady, you also have your personal interests, that is why we want to know that we as gentlemen, we want this

**R:** me what I would say, now as you have come and called us, because it will not be only to call us just few, but it should be for the majority and health education goes on well, because someone to accept something you have told him it is through thorough explanation and he understands, but now if you have not informed us like the way he came and picked me up that we should go and learn, but not understanding which learning, still I never asked him I was like let me go and see that learning to know the learning we are going for so it needs such sessions to continue such that people can continue to…..

**M:** understand…

**R:** to understand, that was my suggestion….

**M:** what do others suggest? Isn’t there any other person with another suggestion on this issue?

**R:** another thing, as you came to teach, are you going to direct us? and are you still a student or you are done?

**M:** I finished long ago

**R:** you finished long time, you are a health worker now. Are you going to direct us where to go for testing? and if we are found positive, are you going to provide us with drugs? [all laughed]

**M:** they have laughed, first answer him if you have the answer [all laughed] answer him if you have the answer…., but the issue is, today as I have come here, let me hope it was your first time to see me, but it was not the first time to hear about issues concerning HIV/AIDS and it is not today that you have heard that in Uganda we don’t have any hospital, in life you have grown up to that level, you don’t have any person either a neighbor or workmate who has ever suffered from HIV/AIDS and you have never seen anyone on treatment and where he gets the medicine from today I have come here when majority we know that people get infected and they get treatment, not that I have come with sucks of drugs, but I don’t deny there are those who are positive and are getting drugs, there are those who have tested and those who have not yet tested but they face a number of challenges, if anything, you say you have made a shirt, the first shirt if they show it to you, so if we just remained at that, still some could be still wearing backcloths or others moving naked, but everything goes on….

**R:** changing

**M:** there is improvement, and this improvement is through getting individual suggestions, someone would suggest that maybe we make some reductions here, that is why you can see others are small, other are big, today everyone buys his preferred size, but they have been suggesting and suggestions not through gathering many people maybe in a field that everyone puts forward their suggestion, but you cannot get such a condition, but if you can get a small number like we are here today and people put forward their suggestions, still it takes time am also not alone that am doing it but there are many others, to ensure that we get your ideas to see how we can make changes, because if you are creating a system it doesn’t depend on one person, if tomorrow I die, it doesn’t mean that the remaining people will not get help, they will not get drugs, that is why I cannot tell you that as I have come here today and maybe if I go to Mbale am the one with drugs, you have to come to Mbale, so that is what we are doing now, who else has a suggestion regarding our topic today?

**R:** so some of us here, we have not understood that testing method, they didn’t understand it well, explain to them they did not get it…

**M:** the ministry has a new method of testing that they want to bring closer to the people, this method not like the old methods which uses blood, it uses saliva, the test uses saliva, you rub it at the upper and lower gum, then you place in the bottle which comes along with it, on the bottle you can be able to read and understand whether you are positive or negative, and that method how I can make it possible for you to understand it, it similar to the pregnancy testing stripes among ladies, in that even though someone is deep in the village, can access…

**R:** the pregnant test

**M:** yeah….

**R:** even though she is not educated, even though am not educated, can I be able to know or they are different, it may require to read where it has reached on the scale so that I determine whether am positive or negative

**R:** imagine how I get it and take it to someone to inquire that how it is, or even though am not educated I can be able to know that it is like this

**M:** still you can be able to see it, but as anything new is coming, it comes along with instructions, but before we reach that point, what we want to understand today that you as gentlemen, would you like to have such a method when you can have self-testing, the old method is available and even this one is available, it is up on us to select what to use [noise from vehicles]

**R:** we need them all, because there are those who cannot afford to reach the health facility [noise from ringing phone] so that testing method where you can test from home, it is very good, Isn’t that what you are telling us that there is a new testing method that going to start…..

**M:** that is exactly what I said…..

**R:** that is what you are going to teach us…..

**M:** that is what we are discussing

**R:** now that method, does it test in only women or even gentlemen?

**M:** as we said, the HIV virus, whom does it affect? let us start from there, Who does the virus affect?, gentlemen only?

**R:** start with the young ones also….

**M:** does it affect the young ones? the girls only? Or both?

**R:** it affects all…

**M:** all, now if this stripe tests for HIV, do you think it tests only among the males only? [all laughed] let us ask that…., do you think it only tests among gentlemen only?….

**R:** everyone [laughs]

**R:** everyone

**M:** now you as gentlemen, what do you think about it if someone introduces it, will you go for it? or what can you do?

**R:** what is its cost? …

**M:** now that is the government decision, but you as public if someone brings for you such a method

**R:** it has no challenge to me, me as an individual….

**M:** how do you think it may benefit you if it is brought?

**R:** to know the life status

**M:** do you think it may be quicker than the previous method, if it is brought? What is your suggestion sir? [laughs]

**R:** [laughs], anyway it is a good method, but for me I will know it within me personally, but those counsellors so I may go to the counsellors and they continue to counsel me but when I have known my status, because sometimes even though they come and test here in tents, still people ask you that let me see you results and if you deny, they we assume you are positive, but now here if am testing and I even throw it away in the latrine, you be like I died long time [all laughed]

**R:** it means work early and eat your things, to look for the drugs not….. [all laughed]

**M:** the aim is to seek for drugs so that you can live longer, because where we have reached today it is not like those days as he stated it, the HIV now days, as you were infected, then they start moving you towards your grave…..

**R:** for us we have a brother they were pretending that it was witchcraft, he has produced eight children and they are all died, but still he wants to produce, he was even taken to the shrine, they removed him and now started him on treatment, but now he is dating young campus girls and I cannot tell someone, this love is blind though you tell your sibling that; that person is positive, he will just abuse you, he even reports, the person hates you and may even kill you

**M:** true….

**R:** you have not lied, it is true

**M:** ok unless one has a question, we have come to the end, Is there anyone with a question regarding our topic today? that he want to ask, nobody has a question

**R:** some Busoga even sang some song that HIV is a disease that causes the AIDS and you filled with…... [All laughed]

**M:** What does it mean?

**R:** that HIV is a disease of long time, it brought the HIV

**M:** ok gentlemen, am so glad for your time
