# Supplementary material for: Prevalence, associated factors and perspectives of HIV testing among men in Uganda
Source: PLoS One. 2020 Aug 7;15(8):e0237402. doi: 10.1371/journal.pone.0237402 (PMC7413494; doi:10.1371/journal.pone.0237402)
Supplement: S1 File — (ZIP) [file pone.0237402.s002.zip › manuscript data/FGD men5-Eng.docx]

**M:** now let me introduce myself once again, my name is AA and the reason for our gathering here today is to discussion ideas on how we can improve the services of HIV testing while targeting gentlemen. What changes can be made in relation to the current situation of HIV testing, and before we go ahead, I would request that we all introduce ourselves so we can know each other

R, I am Is

**M:** let us be loud enough so that we can capture all the voices

**R:** I am Is
**M:** Is…..

**M:** nice to meet you, others

**R:** Jn

**M:** sorry…….

**M:** Jn

**R:** Ms

**M:** Ms

**R:** YC

**M:** YC

**R:** Nb

**M:** Nb, now am also AA, sir what about yours

**R:** Le

**M:** Le, before we continue, the first question would be, I would like to know you as gentlemen, the issue of testing for HIV, what do you have to say about it?

**R:** that issue is good to know your status, though some procedures are not good the way you conduct them.

**M:** sometimes you make it had hard for people that go for to the hospital for testing and sometimes when you come for testing, people are for work, you come during working hours so someone cannot leave work, if you would make it during the after work hours so when one is off with work he can test when you are still there, but when you come for testing, you can at midday and end at 5pm yet for us workers by that time we are still busy, we end at around 7pm or 8pm if you can make it at around 9pm someone can go and test, this is for us who are working. The second, you people who are testing, you have led to increased HIV spread, you test someone positive, but you keep it secret, it is good, though it is also bad, if someone knows that he is positive, he will know it all by himself though to me I will not be knowing. I will not know because he will not tell me, though you the counsellors you will be knowing, but the majority when they come to know it, they just go on spreading, because no one knows that he is positive, except when he gets some signs, that is when you can start realizing, so because he has not informed anyone you still remain unknowing that an individual is positive. So they just continue spreading the virus, but whoever has decided to test by themselves, it should be public not private, it would be more meaningful, so know that you friend is positive and you make some arrangements for him to avoid further spread, but that method of privacy, no one knows even getting ARVs are in privacy, it is so bad and dangerous, you have contributed to spread HIV in Uganda, it would have ended long time, but whenever you hide, even drugs are provided privately, no one will know that so and so is on ARVs. The reason why some people want it to be private, they don’t want to be known, he will love someone and continue spreading, but if it is known, and being positive is not a crime, it is not much different from being of any other disease, they are all diseases. You should make it public not private and we know that our friend is having such a problem so we support him like this….., because HIV is known now, it is no longer scaring in Uganda, we know that it is there, there is some drugs; both complementary and modern medicines and people are using it, the challenge is that the secret cannot help us, it only help the positive one to spread to the negative individuals. If you make it public when people’s aim is to get treatment but not spread, then why do you make it in privacy, we should make it in public, if one fails to make it in public, he should not get medication, he will get signs and people will know that he is positive because he has not got treatment, he will not spread HIV to others, though majority; I tell you they go for treatment simply not to get rashes, to avoid cough but when continuing with the spread, that is why now the positive are more nice looking than the negative individuals, because you provide them drugs which nourishes them very well. The wondering issues, you can go to the health facility to seek treatment when it is not available but for them it is ever there, it ever available, though diseases like malaria, they will always tell you there are drugs in the health facility and you will die of malaria yet the one of HIV is still living, so you should make it public not private, the world will change for good, that is my suggestion….

**M:** ok others what do you have to say about that issue, you as gentlemen, because the aim is to get ideas from gentlemen, what do say about testing for HIV virus

**R:** the issue of testing is very good……..

(Interruption from others)

**M:** send him the microphone…….

**R:** they are recording on camera……

**R:** ………, testing is good to know your status if you are positive then you start on treatment…..

**R:** in privacy or public……

**R:** in public……

**R:** then tell them why public…….

**M:** let him give us his suggestion as it is, because even others we have not yet heard your ideas, others what do you think about testing for HIV virus, because what we have talked about, we want to know as per today, you as gentlemen what do you have to say about testing for HIV

**R:** me I think it would be required that every gentleman get tested, they test and know their status, it is very important because, because if it is a must that you should go and get tested, it helps to know the status, the situation is not good, but you can help your friend and say someone has ever used this particular drug, or you can use this drug

**M:** others what do you have to say….

**R:** if like the way women go when they are pregnant and it is a must to be tested, even gentlemen when they are sick either suffering from malaria, they should test him inform him what he is suffering from…..

**M:** even though you are suffering from malaria, they draw blood and test everything…….

All respondents, yeah

**M:** now still on that very issue, as per today the way things are do you have any challenge that you can state, this gentleman had mentioned some challenges that they face when they go for testing, others what do you think you could be facing when going for testing, because as far as gentlemen today we don’t know what exactly…..

**R:** if you married, you may find it difficult to inform your wife that I am positive……

**M:** uuuuuh, what of others what do you suggest?

**R:** now as you distribute condoms to people for free, if you could distribute the HIV testing stripes to people in plenty as you distribute the condoms, because that condom the person can use it three times but the fourth time, he will not use it, yet you are not tested, but if you can distribute the HIV testing stripes in plenty as you distribute the condoms, when she gets a gentleman, she will first demand for testing, if you are negative then you continue, if positive then you separate, bring those stripes and teach people, many people don’t know that they exist yet they are there, yet they are very easy, you test and know your status. If I get whom I have loved, then we test there and then if we are all negative then we go on, even though they are not update that you cannot recognize it if it has not yet established itself, but they can help as the same way condoms do, so distribute those stripes to people and teach them how to use them many people don’t know them yet they can afford to buy them yet they don’t know that they exist, teach people, other than encouraging people that when you circumcise you will not acquire HIV, that is wrong, that a circumcised gentleman does not acquire HIV, that is wrong tell people that even though you are circumcised, you can still acquire HIV, you still acquire Syphilis, you can acquire gonorrhea, so teach people that stripes are there and you test yourself and know your status. So you test with your wife, friend or any other date you may be having, so teach them to people, advertise them and still provide them if you really want to help and not spreading, but to me I see as if you want to spread the virus as you do those tests in privacy.

**M:** now you have brought in more ideas, there is one idea that I would us to continue discussing about, as we share ideas….

**R:** like which idea….

**M:** you have heard him suggesting that we bring these stripes when they can be available, so do you think people today……., can you test yourself, can gentlemen test themselves if the stripes are provided

**R:** me personally I can test myself, but……..

**M:** self-testing

**R:** yeah, but in addition to the other gentleman’s suggestion, you people who do the testing of HIV, you have contributed to the increased HIV; ask me how? You ever telling us to get circumcised that a circumcised person doesn’t acquire HIV meaning my life if I get circumcised, I stop worrying thinking I will never acquire HIV yet we have seen even the Moslems who were circumcised long time, they are dying of HIV, you should change that message, make it clear that circumcision just makes someone clean but you cannot stick on it that when you get circumcised you cannot acquire HIV

**R:** that he cannot acquire HIV, you should look critically into that issue

**M:** I have heard that one……, but when we are ending please remind me I will make some elaborations……

**R:** because they are make who explain to us, there where we stay, they say please come… [left hanging]

**M:** I will add some elaboration

**R:** that is my suggestion, madam….

**M:** but there is another suggestion that you had talked about that you can do self-testing

**R:** yeah I can test myself, but like my father has been here, I maybe discovered, there is even my friend whom I know, they had just finished their introduction, but the gentleman, I even don’t know what happened to him he went and tested himself and he was informed that he was positive, since then he is still admitted, the woman he has just introduced is still on their other agreement of good and bad situations but still, the conditions indicates like that was the end of their family, what can be done for such scenarios

**M:** ok, I will also answer that…., others what do you have to say

**R:** still like my fellows who first suggested, I have no much difference with them because me I know that in many circumstances when one knows that he is positive, in most cases they just continue spreading, because even the condoms they cannot put on, they will develop that heart and infect those who are negative, so it would be good if all people test themselves, because when these people get to know that they are positive they don’t mind putting on a condom they say I will not die alone, meaning he will not use a condom he will go on spreading to others, he finds even the one who is negative and infects her. So it is very important everyone knows his status….

**M:** now what do you think that we would help the gentlemen to ensure they come for testing, because as per today gentlemen don’t go well for testing, but we want some improvement so these suggestions should be from you and say we as gentlemen, if you can do this, we as gentlemen we suggest this….

**R:** sometime back, I think I was still young, I think you should bring back that method, to visit those people and ensure them that being HIV positive is not the end of life that you are going to die, because some time back in our area, we people could acquire that disease, we could see ambulances coming to visit them that issue but I don’t think that it is still done, it is you personally who should mind about your health if you come to know that you should go and line up for drugs, such things, they need to be……

**R:** I think what she is saying is that what they should do because gentlemen, we are not going for testing like women, for they test especially when they are pregnant, for them it is done compulsory……

**R:** they tell them to bring their husbands to be tested…….

**M:** yes they tell her…..

**R:** but still they may discover him when positive……..

**R:** the challenge, for us we are more involved in issues of money, we are ever busy looking for money, that is what I told you that try and come to our respective workplaces as you have come like now and come in that particular time when we are not so busy when someone is done with work…..

**M:** like at what time …….

**R:** the evening time is better like now people are busy, they are going to search for their debtors, if you come at midday and you are ending at 9pm am sure someone can create time to come for testing

**M:** testing…….

**R:** but if you stop at 5pm or 6pm by that time one is still busy looking for debtors…..

**R:** they fear going there, even though you put in the compound that there is free testing, there are those who will not go and they will decide to stay in their conditions, they don’t want to test to cause worries, because there are some people who can be there when they are not sure whether he is negative or positive, so such people fear to test, they say they don’t want to get worries that I am positive, so people have that fear

**R:** so it should be compulsory…….

**R:** if they tell me that I am positive, I will start to worry, so that is why even though there was testing they fail to……[left hanging]

**M:** to come….

**R:** because one will say if am discovered that am positive I will start to worry, that is what most them fear…..

**R:** they have not taken our rights…….

**M:** and still we don’t want to constrain with human rights, but all these come for easy organization, now let me ask another question; if someone brings a new method when these testing strips are readily there can you do self-testing? [noise from the background], have you ever heard about it…., please come closer so that we can discuss…..[all laughs] because on that very agenda we are, because I was about to ask you, have you ever heard about that method; for you have said that you have ever heard about it……

**R:** me I have ever heard about it on the radio…..

**M:** yes, first explain to us, there could be others who never heard about it

**R:** I hear you place that stripe in the mouth, and it looks like a thermometer [**M:** uuuuh], so it will indicate whether you are positive or negative [**M:** uuuuh] now for me I say if they are to establish such a method in public it will help, because you know that if I give it to someone here it will help determine this one is positive, this one is negative, this one needs treatment or doesn’t need treatment; but for me I supported that method because I heard it over the radio

**M:** actually I wanted to elaborate more on that method, that method; now like people are not coming for testing, so the government has a suggestion that if anyone could bring these stripes as it is in women; if you are not sure whether you are pregnant or not, if you want you can go to the health facility, but even though you move to the shop, you can buy your stripe, you go back and test then realize that am pregnant, so if someone can establish such a method, that is the one the other gentleman was explaining; that stripe is used in the mouth you rub it on the upper gum and lower gum then place it in a certain bottle then it will read, but it is read in the same way the pregnancy stripes are read, hope we all know how the pregnancy stripe is reads……

**R:** one line and…….

**M:** that you are not pregnant…….

**R:** two lines then…..

**M:** you realize that I conceived this time, so even the other stripe works in the same way, who else has ever heard about it except hajji

**R:** me I know it, I can even use it…..

**M:** so what do you think about it

**R:** but it also works privately what we have talked about, so if it is to be used, it will not have a name well written on that say Le, I can use it and place it there though it will not indicate that Le is positive or it has indicated two colors, unless you tell me that you have write your name on it [some laughs]

**M:** but is your alone……

**R:** that is the problem it makes no sense when it is mine alone, because let me tell you, we people we are not easy, I will leave here and know that yes I have used it and I have known that am positive but the other will not know, from the heartedness I have I will go and infect Mrs. Ma when I actually know my status; even though I may not love Ma’s wife I may love any other lady, even my other friends will come and love her but when I slept with her and they don’t know that I loved her also, that is why HIV is in a chain, you can acquire it when it from your daughter; I have one lady I know she is positive but she is so healthy, she is so nice, she is my relative though she is pregnant now; and the person who impregnated her wasn’t the first or the second, implying she doesn’t mind whether you have used a condom or not she doesn’t care, but she is so nice and healthy than us all who are here, and if you find her you cannot fail to propose to her she is so nice, so that privacy is bad, still that method of the mouth stripe is still private; unless you are to make it compulsory; you do it when it is compulsory, you have all to do it and know, because HIV disease is like any other diseases say malaria; but you love someone not knowing she is positive; but you only know it personally; there is no other reason why you hide it because it is your alone; the method is good and when established many can afford to do it but how does it work, the privacy is what I hate that it is bad, it increasing HIV spread; the public one is the only secure way and the world will know that Lawrence is positive so that you can protect yourself and stop having multiple partners, you will die early; you may find that maybe Me knows some herbal and he can help [**M:** and take], and I take so it can help me, but if I do it alone, and I find that I am positive, of the ten people you find four are positive; is it not true; but as we are seated here and you find that I Le am positive [**M:** even that one I will do some elaboration] when you know that Me is positive, Ronald is positive, it doesn’t affect so much as knowing alone that you are positive

**R:** that would be very advantageous as you test yourself and know that you are positive, you go to the health facility and get treatment……

**R:** but to me I will not know whether you are positive

**M:** now that……, ok others what do you have to say about this method if it was established, because you find it difficult at the health facility

**R:** me I would request that those people who get their short term relationships [**M:** uuuuuuuha] I think it can help such people so they can use it with the person he has got. So you can discover your statuses, like those who just meet on the road at any time, so they can use it at that time, so it can be helpful to know whether one is positive or not as you can easily test at that moment

R we should appreciate that they are advantageous because if you get an instant date, there those who get dates and they have sex there and then so if they have that stripe, it can help them to know as one maybe positive when another is……

**M:** negative

**R:** so you can know immediately, that is why I say that it is very important, because if you meet now, then you can test immediately then you know this person is positive or not and you separate or even her to know that maybe you are positive and she can abandon you so that method is good, me I support it….

**R:** they should supply them in plenty, because they are expensive [some laughs]

**R:** they should be even in the pharmacies…..

**R:** they should give them to women, as you said that gentlemen fear to test themselves, so when the women know there status, they will be giving them to the gentlemen,

**M:** if you can be strong and test yourself

**R:** if she is positive and you find her she will tell you that I forgot it at home, you will not test, so we should all have them so not only women……

**M:** yeah everyone should be able to test individually

**R:** you will not test alone because you will not know whether am negative or you are negative, so you should do what…… [**M:** test] so that methods becomes important and it was being used, it wouldn’t be bad….

**R:** and still to test I maybe positive but I don’t know the status of my partner and I have not told her so even though you are test she will fear she will tell you I got it but I don’t want or I have my own challenges…..

**M:** so one can tell you the truth

**R:** she can tell you the truth before so you go ahead knowingly, but there are many women you can find when she is on ARVs but when the gentleman is not taking them she want to increase her CD4 but the gentleman wants to produce, so the wife tries to increase them very fast; because women are so smart, so as you said that if you give them to people; someone will date her knowingly that I am positive then maybe you could just say we have met I am also positive…….

**R:** like those who advertise themselves in newspapers that for me I want a positive woman who is on ARVs because am also taking them [**R:** it becomes very important],

**M:** now let me ask; ooh, you had a suggestion

**R:** they should distribute them in plenty as they do for the condoms

**R:** and they should them out to people for free

**M:** let us talk about that issue, if they are being established and they are for purchasing, you as gentlemen, can you afford to go to the shop and buy them….

**R:** as you condom for that one who minds about his life, he should do what……[left hanging] as I said that when one is hearted then he starts spreading, they say I will not die alone, but if you know that you love your life, so still have to buy it

**M:** have to buy it….., now another thing……

**R:** now still you help us, if they are to be at the shops, as you there are Indians and Chinese [laughs] [**M:** laughs], even Ugandans are bright, they are going duplicate them [**R:** the Chinese], yes they are those to be fake or invalid, it can be invalid and it cannot tell whether you are positive or negative, the major thing is to teach people those about stripes, so if it turns invalid it becomes not correct; it has not indicated whether you are positive or negative, when they find whether you are positive or not, because for me I know, hope you getting what am telling you; me I know but those who don’t know, please teach them so that they can get to know about those stripes, like you say about condoms, you teach them how to wear a condom; but teach people that this is how the stripe works and how it appears in different statuses; and the storage conditions [**M:** you have brought very strong reasons], yes they should be known not just a matter of finding them in the shops, then they will make more fake ones [some laughs]

**M:** still on that very issue; I have got a thought, if they are established, you as gentlemen, where would you like to find them…..

**R:** where we find the condoms, the same way I to the clinic and requests for a condom so even that one when I can go to the [**R:** health centers] and say please give some condom [**R:** give me a condom] so where they sale condoms from…

**M:** it is where you want to find them

**R:** because condoms are no longer a new thing…..

**M:** yeah it is not new…

**R:** those days someone could come at the shop when in need of a condom and he would wait till people are done [all laughs] but now one comes and say please give me condoms…..

**R:** you see they even take them in schools, even in toilets we find them even in the markets you can get some places and place them there…

**R:** that maybe whoever pushes a five hundred shilling coin then gets it, because there is nothing for free, because even the condoms for free are not there

**M:** so if we can make those machines when can just push in [laughs]…….

**R:** he just pushes a coin then it comes [some laughs], let us say five hundred, that method also can be better

**M:** now the stripes you have talked about many advantages about it, are all the advantages about it done [some laughs] because I have a question as we have gathered here, how many knows how that stripes functions, do you think people know how that stripe functions…….

**R:** that is what I told you…..

**R:** these training sessions as you did for condoms, this is how you wear the condom if you have finished using it, you remove it like this…..

**R:** you place it here or you just burn it after use; it should just be disposed of anyhow, so they should also teach it to the public, because me personally I know it but…….

**R:** for me I have ever heard about it and I don’t it, and I don’t know how it operates….

**R:** and some of us are brave, maybe if you had carried one, I would be able to go ahead and teach it to the rest,

**R:** people should learn about, it should be on TVs,

**M:** still these are the suggestions that we are gathering and we are still continuing; if the method has been appreciated by the public, so then we organize so that we extend that method and people come to know it, so let me think……..

**R:** but it also has challenges [**M:** yes…], let me tell you, if someone has just acquired the infection when the infection has not yet spread, it may not be able to detect that that person is positive, yet the person is infected, so be aware, you may put all you trust in them

**M:** what about the blood, what do you have to say about it…..

**R:** but this blood test is better than that of the gum stripe; blood test sometimes it is good, but it also has some challenges, that is why you should go for at least three times while testing; because if you have just acquired it, it means it has not yet spread into the body; you may find that the person is infected and it will tell you that he is not negative; so you may be eager and say now it is done and you continue being infected; so that is why when you have just seen someone, then……

**M:** it doesn’t mean……

**R:** that that person is negative, still use a condom; as you continue being together still test and confirm that you are negative after a period of time then you can confirm but not on the first time test

**R:** me what I say, even that stripe; as they say that it requires to test for at least three times, so even this it should be better when used for at least three times

**M:** you can still bring it at the end, I will do more elaboration

**R:** it is true, you will test after the three times; but still my partner that trustworthiness will not be there; still you will find that you have tested then used a condom and your partner just goes like that….

**M:** so such a thing, you as gentlemen, what do you think about it….

**R:** they should add more health education sessions…

**R:** as I said it is good…

**M:** let me first listen to this gentleman….., yes

**R:** what I say, bring it, but also strengthen the health education, because the major cause of problems like you women; after being taught here many establish it as their job, that I may leave here now when we have tested and we know our statuses, but as soon as we have just separated, then you go to another person [**R:** you go to Peter], at Peters place because he is waiting for you; that is your role as women, so if you continue teaching about it and encourage people, because these things of testing are there and we do test ourselves and the positives are there, but the issue is that we have pressure; So after knowing am positive I feel that my life has ended and all plans are gone and soon this year I will be dead; but that issue has not been taught well within the community to reduce that pressure, even though you will establish them and do what…..

**M:** meaning that pressure is there and…..

**R:** even though you do what….

**M:** wait a minute and we hear his suggestion…..

**R:** I have told you pressure, as this one said that you test while from work, what if you inform me that am positive; that care is what we are in need of, when you tell me that am positive; because I know you…..[left hanging] but those heath education sessions is what you should strengthen so that you encourage people that even though you have known that you are infected by the virus, but you are not going tomorrow or the following day, remain doing your usual work and we shall be providing you with treatment; that is what I told earlier that while still in the village we could see some vehicles coming to check on the patient and they were looking for such people but now days you have to mobilize yourself and go for treatment

**M:** that issue of health education, you have talked about so much, but what I may ask that if one would wish to bring you such information regarding this stripe, regarding testing, how best can it reach you, which channels can easily reach you as gentlemen; because when we reached on this issue some said they had ever heard about, others had never heard about, so how can one reach you with the information and it can reach the majority

**R:** now most of us; like today do you that by the time I leave here I have learnt something; sometimes I go back and switch on the radio though when am tired but when you reach me where I am like as you came here in the market; I have learnt something that I am going with implying if you are to tell another person that you can hear it from the radio, yeah they are correct; but when you speak with your fellow human face to face unless you don’t have the brain but they can understand. But someone who has explained to you face to face, even though you are stupid still you can grasp, yeah those health education sessions, not only on radios, but to reach the real person as you are here getting our views

**M:** uuuuuh, and others what do you suggest

**R:** they should even start advertising…

**R:** even vehicles making drives can be used, if it stops somewhere all the people…….

**R:** even that which moves around that informs people about circumcision……

**R:** the one of Makerere

**R:** that come and get circumcised, it for free…

**R:** and still when it is moving around, some go speedy, but it should have a moderate speed while advertising, you can hear many people complaining that it should stop; may be like ten minutes while emphasizing that issue; where it finds many people it should stop and inform them and the other thing even TVs are very good, but they are full of no sense issues, but you should make such adverts that teach the public, so in every program the government should make it a must that every television should advertise them because televisions are watched by many people that is why……..

**R:** there is NBS, WBS……

**R:** yeah all televisions should make adverts and it should be compulsory imposed by the government, because many people watch TVs, so all the time in different programs, they should advertise

**M:** what about the phones

**R:** even phones……

**R:** those messages

**R:** messages should come, those on whatssap should get theirs, then our with no whatssap they should come and informs us in all languages in Luganda, English, Lutooro, all languages should be catered for so people will be able to get that information

**M:** we have talked about many things, but there is one that I have not heard about that I would like to ask, let me start like this, you as an individual, do you have that braveness to test yourself, are you not scared anyhow, and do you think that gentlemen will be brave or do you have any issues that you foresee if the testing mechanism is removed from the health facility and then remains on an individual to do it, do you have any difficult that you may think of that this may happen or any other any worry if there is any change…

**R:** madam to me I think we are now firm because me I have seen many times that they and test within the compound there, many people have tested and one will remain with his results; that we are no longer understanding….

**M:** that is what was confusing this gentleman….

**R:** but all that still, were always see those people come here, but you can see z gentleman enters there and get tested and he leaves when he is fine

**R:** he will say, yeah they are here so let me test myself, they are no longer like that those days ….

**R:** in addition to this gentleman’s suggestion the pressure has reduced, you all aware that people fear pregnancy than HIV…..

**M:** but the other gentleman talked about pressure

**R:** pressure [some laughs]

**M:** I don’t want us to ignore this point of pressure……

**R:** it maybe some percentage, but it reduced drastically, that is why even HIV has increased people now fear pregnancy [**M:** laughs], she can’t say I shouldn’t get HIV, they are only worried of getting pregnancy, so HIV is considered as any other disease because ARVs are readily available, so that pressure you are talking about is no longer too much, it reduced seriously

**M:** implying; in view if these stripes are established, it will not have any major challenge

**R:** yeah, they will not fear….

**M:** are you sure there will be no fear in all gentlemen…..

**R:** what I hate about those stripes; there are those who go for testing when they are sure that they are negative, now if he tests and……, yet after testing in the health facility the health worker seats you down and counsels you; but if he tests himself alone there may be some committing suicide; because there are those people who go when they are sure that they are negative with no hope of being positive, so just imagine when he tests alone without any counselling…..

**M:** that is why I want us all to make suggestions; what can we do…..

**R:** because me I know that after testing someone, the expert medics should counsel him and he will be strong [**R:** there should be more sensitization] but if the person is alone and he never expected to be positive he may even commit suicide immediately [**R:** that is why we are saying there should more sensitization]

**R:** the same thing madam that stripe if it is done that it can prevent; being a gentleman is so selective, you cannot say that I will have sex with you now and you give you stripe then it will provide me the results [some laughs] and I will gain any strengthen, my weakness starts immediately [some laughs] with all my annoyance that I came with, that is the way I will have to move back….

**R:** that stripe is like the way you see condoms now; that I can wear it now and then after remove it. That stripe, my emphasize is that establish it with increased sensitization…….

**R:** that stripe unless I will test and my partner also tests and we are all negative, that is when we shall get some happiness, but I have told you if it comes even though I had come with all the happiness towards you, once I put in the mouth and it tests me or either my partner that is positive, even the condom I will not wear it; we shall just separate, though if we are all negative then we can suggest that maybe we can use a condom; if it was preventing, then we would emphasize that it should be established but as we have said that sensitization should be increased

**R:** add on sensitization session…..

**M:** that issue I have heard it well it has been so much emphasized, yes others what are your suggestions…..

**R:** that is what I said that it is going to help us somehow, there are many people who test in privacy; what he is saying that if he finds his partner positive that he will not wear a condom now why to wear a condom when you have not agreed, if you love a lady and she is positive, she will inform you, there is a conduct that can easily explain the persons status, she will tell you that yes you have loved and I have loved you too, but I have my own problems, will you love me in that state; there are there who can say the truth; then you decide to just love her because of her beauty and use some other methods; so he enters the deal knowingly, so even though you are test when she is positive and you are negative, he will wear the condom when he is steady and you will have it accordingly because he will be knowing, there are many people who test and know their status, then they just keep silent…

**M:** so no that point therefore, this gentleman explained it well, let me first ask some questions about it, how we can mobilize gentlemen to speak with other people next to him if he has tested himself; so what can drive you as gentlemen to ensure that you can share with other people about your testing

**R:** please pardon what you have asked

**M:** what should be done to emphasize gentlemen to start sharing their testing results say with their wives or girlfriend, but any other person whom he may feel comfortable, and shares it to avoid silent death…

**R:** in fact silence…….

**R:** he informs others and they can help him…..

**M:** yeah and share with someone

**R:** most of the time they keep it as a secret [**R:** still it is secret] [**R:** even though he is negative, he can’t say it], [**R:** unless he found there one or two people] he cannot share that information

**R:** if he says that am positive, I tested myself….., I have ever seen one friend of mine here [**R:** still maybe when you went together] wait a moment

**R:** that is what this one is saying that the results comes and everyone knows [**R:** and to be sure you have to go together as a couple]……

**M:** so what should we do to ensure that……[**R:** now you see…, it requires when it is compulsory and should be public] [**R:** it can’t be, because even the government would have suggested that people should have demarcations] because it affects human rights…..

**R:** but that is what I told you that many people are testing themselves, and they know that they are positive, but the heart of people am telling you; that they feel I should not die alone, many are testing themselves even though you are to go to the health facilities now, many are getting their results, but we don’t have that humane heart, that I should protect others….

**M:** now let me ask, do you think if there is a system when it doesn’t require to go to the health facility when you do self-testing do you think it can help to reduce that belief

**R:** me I can do it if you have taught me, then I will say we were taught in some sessions that if you acquire HIV even though you are with your wife, you are not supposed to do this, and I can understand because you have sensitized me, but now there are those who don’t know so if they get to know that they are positive, then I am dead and I should be waving to all my relatives this year; and I should infect whoever comes near me

**R:** but there is another thing I don’t understand, they have tested me positive and the wife is negative; so how are we solving that issue, let me also ask you; does it meaning separating or we remain using condoms…..

**M:** what should be done when found positive…..

**R:** when I personally am positive and the woman is negative….

**M:** ok, keep me that question to, I have many that am going to answer only that I wanted to wind-up with this then handle them all at once, we shall remain talking about our questions

**R:** now you see the importance of that stripe….[left hanging]

**R:** but that is what I have told you, you are going to destroy all its merits, you just crying crazy [**M:** laughs]

**R:** so this research of yours is about prevention of HIV to ensure no spread of further spread, first answer me that…

**M:** the objective is to ensure reduced spread of HIV……

**R:** you see there could be your study when you aim at further spread…

**M:** no,

**R:** because many NGOs do ensure further spread and not prevention, because it is like you benefit it, by the way you benefit in it, because there is no way you can’t say that it is compulsory, so you will be affecting human rights, which rights yet I will be providing you with treatment; because when I know that you are positive, I will provide you treatment, yet people here complain that there are drugs in government facilities; so how can you tell me that you are positive and I want to provide you with treatment that I will be affecting human rights…., if really you aim is not further spread

**R:** if those stripes come, they will help somehow, because if say Mu has got a lady and they are distributed like condoms, there the lady will inform you the truth because she will be aware, [**R:** yes….] like the condoms are many, he will say I have it at home, so she will tell you on the way that we should go knowingly but am positive [**R:** am positive] those stripes will help so much

**M:** now another thing that I have not asked about as we come to the conclusion of our discussion, these stripes, who else do you think they can help apart from us here as we have been talking, what category of people do you think, that those stripes will help, the others that we should organize and inform them

**R:** even these prostitutes [some laughs], because for her she will be seated on the street with the aim to get a man and get money for food

**R:** and for us we don’t use condoms, but the prostitutes use condoms than us, me too am a researcher because you go for a prostitute when you don’t trust her so you have to use a condom and yet you may trust a lady you meet on the way that she is not a prostitute yet she is a prostitute in her own way

**R:** in all the ways of the prostitutes, the body make money for them; having live sex meanings a lot of money, don’t insist that they use condoms, they have different prices and she will tell you that if you want live sex then pay me twenty thousand, if you are using a condom then you pay me five thousand [some laughs]

**R:** you will know automatically that this one is positive[**R:** these stripes….]

**M:** let us listen to hajji

**R:** if you have worn a condom it is five thousand, so it depends on your capacity [**R:** but in your mind……], we are changing your idea that prostitutes use condoms

**R:** so because you are interested in something at that moment and you have your money, you will not think of a condom you will just say no

**M:** so except those ones who else do you think that these stripes may benefit

**R:** other people, you see whenever there are many people in the markets, health facilities in institutions [**R:** even on the landing site] in institution, on landing sites, in companies, but make errors and give them to school going children as you are proposing that school children should be provided with family planning contraceptives, it is very stupid, in fact it just causes further spread, [**R:** because after use he will just be have live sex], that is what we said that in Uganda today, they are afraid of pregnancy not HIV, don’t give them out to students, give us the mature people or those mature ones like at universities and other institutions but not secondary and primary [**R:** he is the one to teach the rest, meaning if you give me and give the other it means]

**R:** with all those suggestions at hand, people you should avoid sex, you will not have to suffer with diseases, just abandon sex [some laughs]

**R:** no si**R:** we have to have sex, [laughs] we have to have sex pakalast…..

**R:** but it natural by the way, even though you put your young child there two, a boy and a girl when they have not listened to a radio, not watched TV, but when they grow, they will do it naturally, it is normal even though they have not seen it anywhere, the body will demand by itself [**R:** because when you grow, you have to it] yeah

**R:** we should say people reduce on adultery

**M:** so now what other category apart from what you have stated before that we may encourage also to use these stripes

**R:** the fishermen [**R:** even the married] because HIV is more in married couples and the students, not so……, it is more on the landing sites but generally it is more in couples and students, so give them to married couple, give them in plenty, because you cannot know because they are telling you to go and do it but prevention is better than cure, yeah so provide the married in plenty, provide to people in markets, just target places with many people in industries, institutions, university, companies, even our MPs, if they are provided with condoms,

**M:** now as we are concluding, what other suggestions do you have about this stripe that we might have not talked about today, when it is very crucial that it cannot be ignored.

**R:** think about the preventive stripes, because even these we need them but please find some preventive measures; because sex is body driven; all these we just try, but you can also try and see, may be like if you take this particular tabulate, it will infect you because there where we usually test from they tell us there are those rich people who access those tabulates that if they are going to have sex with someone, they first swallow them you should not rest your brains, that is my suggestion….., let us try out as you also fight hard, [all laughs]…..

**M:** let us listen to this gentleman…

**R:** I think everyone should get these stripes even school children, they also have sex [**R:** but they should not be given], they should be provided to all people who have sex, because they also have sex, but its like you saying students should just go unknowingly, but if you see they have started having sex, they should, like you see him and then informing him about condom use, just that they should not be afraid of using the condoms, whenever you are going to have sex, wear a condom because the girl might have been born with the disease, because you have to inform him you can just observe from his behaviors yet he still at school, then you have to inform him never to leave the condoms behind [**M:** laughs], so they should be given to all the people, even though she is a girl and she has reached the age of engagement, she has to know about that stripe that she can use, but we cannot differentiate whom to give and not to give, for I know that whoever have sex should be given….

**M:** meaning that in your suggestions, there should be no limit on age

**R:** there should be no limit, if you see a boy has started dating women, why can’t you tell him to use a condom some students love women

**R:** sir there should be a limit on age, because you cannot say someone below 18 [**R:** now you will lead to infections], now even the primary children should be given

**R:** but there are those who are born positive, she can be at campus but when she was born infected, they are there, you may see that she is nice looking, so you should tell the child that you have to wear a condom and if you see that he so attractive to ladies, then you have to inform him about it….

**M:** what was your suggestion…..

**R:** that there should be continued sensitization

**M:** now it is like I have asked enough questions, are there those who had some questions today….

**R:** my first question…..

**M:** let us ask our questions, we answer them and then conclude, but they will be good if they are in line with our today’s topic

**R:** my first question……

**M:** first wait a moment, we are going into the question session, you are the ones to ask, we should not take much time, but let us first ask questions which are in line with today’s topic, after that then I will switch off this and we ask some other questions, [one respondent laughs] I will be here with you, now questions

**R:** we shall not separate it so much, the aim is to fight HIV virus is the reason why you have brought even these stripes, but even though you are bringing them while telling us that we should go for circumcision that you will not easily contradict with HIV, still you need to teach more about that issue, you should explain clearly to us, because after circumcision me I will think…., [**R:** and many have died so much simply because they say they are circumcised]

**M:** you want it to more elaborated,

**R:** yes, they should elaborate more about it, they stated it wrongly, that if you are circumcised, you don’t easily get HIV, they stated it wrongly, they should elaborate that it is like this and this

**R:** even the advert they made is not good, [**R:** it is not good]

**R:** meaning that whoever is circumcised is not infected with HIV, yet it isn’t that whoever is circumcised doesn’t get infected [**R:** yes, that advert is wrong]

**M:** I think you would have told us first that how would you like it to sound like

**R:** they would have stated that get circumcised and prevent HIV, not saying that if you are circumcised you have 90% chances of not contradicting with HIV, it is like saying if you are circumcised even though you don’t put on a condom there is no worry

**R:** and many have been circumcised only because of that

**R:** as you are telling us to circumcise, explain the way you do it for a condom that this is how you tear it then wear it hold it well, so even the circumcision please explain that this how you will have to have sex with the woman, there will shall accept that the circumcised one is 99% protected against HIV

**M:** ok

**R:** another issue, we want to prevent HIV, but really if that is our objective; the government and other NGOs, if your aim was to prevent further spread, then why don’t you put some demarcations on the patients; maybe not in the forehead, but it can be on the abdomen, on the thigh, whoever is no treatment should have at least some mark on them if you are aiming at preventing; you are considering young children., many young people are at IDI seeking treatment they will spread to the mature and their fellow young ones. For that case I have just talked about young children but even the mature ones, those studying in secondary, many are infected, but if you could put some demarcation and you could only see it when undressed [all laughs] you see how I undress and you see

**M:** we have understood that idea, let me put it back for discussion, what do you have to say about it….

**R:** it will be discriminating people

**M:** let us listen……

**R:** if it is like maybe one with HIV, they can chop off one finger [some laughs]

**R:** but you are the one who went in for her if someone can be identified whenever he goes, then you have constrained his rights; this is discrimination [**M:** and [people will not come out to test] it is not good

**R:** no sir then we shall be protecting ourselves so much…..

**R:** they will no longer fit in public, everyone will be discriminating them so it is not good

**M:** others what do you think about this, yes hajji…. [some laughs] what would you like to be done

**R:** because within themselves they start to fear if you put that mark,

**R:** it is like you have…… [left hanging]

**R:** the person may even die early

**R:** there should be clear sensitization, that when you bring them; ity doesn’t mean that if I test today when we all negative, it is sensitization which is much important……

**M:** let me also here from this one….

**R:** that issue he is proposing of using marks, it is real discrimination; even though you will see that mark, it is there other fear that I have explained [**R:** that means you have been saved]

**M:** let us first listen….

**R:** and still in the world, not every woman you have sex with that you are infected, sometimes you have sex with a positive one and the Lord just protects you, but if you tell me marking, you will find the entire world having marks on them but what I have told you is the sensitization that if you get treatment you can even produce children and you even have your partner and protect yourselves, but that issue of demarcation, the entire world is infected

**M:** now in summary what are you suggesting….

**R:** the entire world is infected and what he is saying then you should not even bring those stripes because the entire world is infected already

**R:** that is ignorance among my fellows, but if you can travel and ever enter Mulago, then you can understand what I am talking about, if there is anyone with a positive woman; when the gentleman married a women unknowingly that she was positive, but when she is aware herself, when the woman is on treatment and the gentleman is not on treatment, then you would realize that it is very important, she is getting medication and having that mark, because those drugs are very expensive, that demarcation is very important, you have not discriminated a person, as you may say about identification, it is like a national identification, like we have similar identification so we are compatible, because there are very few who can expose that they are on ARVs and the woman too is on ARVs, few people can publically say it, or if it is kept privately then it is aimed at further spread, but if it not for further spread, you have a mark, I also have it, then we just have live sex with no worries as we shall be the same

**R:** and still they say if am positive I should not have much sex, but if I get to know, I should be having live sex [**M:** but that is not good also] yet it is also not good,

M now gentlemen, I see we have used much time, let us end there, then I switch off this recorder I thank you so much for all your questions and your discussion, it was so nice, you have suggested ideas, and we shall ensure that those ideas are put into considerations, because as you have suggested them so that they can help you as gentlemen, ok thanks so much gentlemen….
