# Supplementary material for: Prevalence, associated factors and perspectives of HIV testing among men in Uganda
Source: PLoS One. 2020 Aug 7;15(8):e0237402. doi: 10.1371/journal.pone.0237402 (PMC7413494; doi:10.1371/journal.pone.0237402)
Supplement: S1 File — (ZIP) [file pone.0237402.s002.zip › manuscript data/FGD men6-Eng.docx]

**M:** am glad meeting you all gentlemen, we shall come closer so that we can easy capture all the voices, my names are AA, and am grateful to meet you here today in our discussion today, the aim of our gathering here is to get your ideas regarding the testing for HIV virus as gentlemen and what modifications can be made, maybe before we continue I would like us all to introduce ourselves so that we can know each other

**R:** my names are Af

**M:** Af….

**R:** my names are Rd

**R:** my names are Hy

**R:** my names are Ad

**M:** let us try to be louder so we can get the voices clearly…

**R:** am Ed

**R:** Nu

**R:** Gy

**R:** Hy

**R:** Ms

**M:** thank you so much all of you, I also remind you my name is AA, now before we go further the major issue, I wanted to know, you as gentlemen what do you think about testing for HIV virus

**R:** what I think about testing for HIV [**M:** you can speak louder] on the issue of testing for HIV, it would be good someone tests to know your status, but most of the time we gentlemen we lack time to go to the health facilities to test because we assume that we are ever busy that is what I can say about that issue….

**M:** and others what think…..

**R:** On my side I think it would so good to test especially the gentlemen who are in that category and they are responsible, but those people who are responsible for testing people, they should move around like in markets, they make a special day for gentlemen to test and in surrounding places where many people gather

**R:** they should have moved around those responsible for testing for HIV virus

**M:** others……

**R:** on my side I think it would be very good and be required, but those who test HIV virus are not trust worth because every time they come here in Wandegeya, I have never heard of any positive person, whoever brings there results are negative even the known positive, they say they are negative, so we the rest we lose hope

**M:** how do you know that they are negative….

**R:** iiiih, shaaa, even those on medication, they are taking drugs, and you indicate that they are negative, is the person healed or the virus is sleeping, me I see them not being serious [**M:** laughs]

**M:** others what is your suggestion, you had something you were saying…..

**R:** it would be good to test for HIV virus, but you find that we gentlemen, we don’t want to go to the health facilities, yet you see someone has multiple partners and he has one girl whom you also expect that her blood is positive, but as my friend talked about it, those people who are sent for us to test, they can come let us say here in Wandegeya market and they can test like 100 people and more and you cannot get a single positive [some laughs], so even us we are demotivated and ask why we should go there, if am to die I will die singularly, so we are not motivated to go to the health facilities to test and know our statuses

**M:** there is an issue that you are talking about that is bringing questions in me…..

**R:** it would be a good thing of testing for HIV but most of the gentlemen fear more than the women [**M:** why?] a woman can easily go to the health facility for testing for HIV and know there status, but the majority of the gentlemen when they realize that they are positive, they lose moral of doing what……

**M:** doing their work……

**R:** they lose so much that is why when gentlemen get to know that they are infected, it means their death has come closer which is not the case for women, so that issue, I think gentlemen still need a lot of sensitization to ensure that they go for HIV testing….

**M:** actually I was going to ask you, because I see you propose many reasons, we would have been testing; so what exactly prevents you from testing

**R:** gentlemen have a lot of worries or the different fears, counselling is supposed to be given to gents isn’t the one to be for the ladies, the gents should be given a separate counselling

**M:** add more light as we wait to hear from others, what would you like to be done to ensure that gentlemen go for testing

**R:** it is one of the reasons I was saying about counselling that if the gents have been called maybe one day separately only gents, so they come and teach them say here in Wandegeya, emphasizing the advantages of testing, many gents don’t go for such sessions, so they fear that if I am found when am positive yet they have sex always, but he has not got such knowledge that someone can be positive and still live for some time {**M:** some time,] so that is why gentlemen fear to go for testing, but women have got a chance that when they are pregnant, they cannot provide her with services unless they have tested her

**M:** am grateful for you suggestion, you had something that you were saying, what was it….

**R:** [laughs] what I was saying…..

**M:** yeah….

**R:** still I was at that point of testing for HIV, but with more refining what I suggest, I think as the government came up that it decided to help [ the public that everything is for free even the drugs, so they should add more sensitization as gents only as you have done it that you discuss with us gentlemen only, but they should add more efforts in sensitization

**M:** still on that point that is why I asked a question that you as gents, how would you like to be sensitized, this one had some suggestion, though others what do you suggest

**R:** what we suggest is separate sensitization, the counsellor to sensitize only gentlemen [**R:** so they counsel you even though you have tested you can spend even 80 years when you are alive, it doesn’t mean that whoever acquires HIV that is the end of life, so it is not yet clear to people, but they should know that even though I have acquired it I have gone, that is the end of my life, so we need sensitization

**M:** so that you can be strengthened…..

**R:** so we can be strengthened, that is how it should be…..

**M:** ok

**R:** maybe what is missing among gentlemen is one [**M:** uuuuh], gentlemen fear and as we are here if it was testing for HIV, you would only get one person [some laughs] or two

**M:** and why…..

**R:** because most of the gents when they know that they are positive, majority think of further spreading but not starting on treatment, because in most health facilities that offers HIV care majority are women, that gentlemen are not going…..[**M:** and that is where there is a major challenge, that is why we want to know exactly why], but the reason for not going is not being sensitized, not getting enough counselling that you as a gentleman you can live as a woman does what….. [**M:** lives…], lives and even produce your child when she is healthy, the thing that they are…. [**M:** not aware] not aware, so that is what we request that there should be more sensitization, the counsellors should come to gentlemen and seat them as here, they should come and counsel the gents that someone can still live for over 30 years with the HIV virus, that is my suggestion….

**M:** what was your suggestion sir….

**R:** maybe on that issue that gentlemen don’t go for testing, in fact even the government and other NGOs, they have foregone the gentlemen, they have tried to gather women almost everywhere and in the process they get the chance of a sensitization, for us gentlemen we are ever busy, you think of leaving your work to go for testing, there is no food at home, so you find yourself [**M:** failing to go], failing to go for testing…..

**R:** another issue even the country is faced with a challenge, I call it a problem personally though they say it is so easy, they move around here while advertising that you go get circumcised and prevent HIV, is it that a circumcised person cannot be infected, yet we see Muslims dyeing every day, so even that was established to help people but it is very dangerous, in that situation like that

**R:** so it is like they are just pushing you [all laugh] that you have now survived [then laughs] so we need so clarification on that [all laughs], [**M:** I will do some elaboration] that a circumcised doesn’t acquire HIV.

**M:** so that question you will ask again towards the end, I will add more light that you may need, but there is something that you raised that cause me to think that you need sensitization for gentlemen only [all respondents, uuuuuuh] but where, which type of organization would you like to have where someone can appreciate that really you can come, one of you suggested that for us work, what….

**R:** they have to sensitize us [**M:** but from where….], they have to sensitize us clearly maybe from our respective workplaces, because we don’t stay home and still they should inform us that maybe you will go say like at Makerere hospital, that is what they will tell you of the true HIV status, but these people they send us on street or in the compound of the market here of Wandegeya, but as I told you that they can test over 100 people and they will not get any positive person, [**R:** but how does it happen….] how… , as we say the world is standing right now, because even us here we don’t trust our blood

**M:** so that is very interesting, because, it is like you don’t trust these tests….

**R:** now for I was saying, that that sensitization would be at workplaces as he suggested, but as we have gathered here, when we have just got permission that today we shall have a health workers, because majority we work from here, we gather and learn from here that can be easy for us, at our respective workplace….

**M:** like which jobs that gentlemen are more involved were we can find them

**R:** there are many jobs like boda-boda riders, meat sellers, they can be here in the market and they also gather here for a few minutes, and then they go for their work….

**M:** what other workplaces

**R:** like the taxi drivers….

**M:** but what about if you are outside the market like here, where else can you find gentlemen

**R:** the taxi drivers

**R:** so if you have not met them in the boda-boda stage, you can get them at the taxi stage, and the remaining people who are doing casual work, they just keep on moving around, others are not stationed that they are in one place, so such people you will not be able to gather them maybe at their workplaces, where you find ten, teach them from there that is how it should be;

**M:** ok….

**R:** then another thing moving also in the villages, but you just end here in towns, the virus has left town and now in the villages

**M:** that is why I asked you, that beyond here

**R:** now you should move around villages and educate them I will leave Kampala when am infected and go to the village, then I will get a secondary school student, infect her and she will infect others, so if you can sensitize in the villages, it will be better…

**R:** even schools, there are children, because girls no longer have standards in schools, they can date their fathers, grandfathers, they are after money, and she infects her fellows, you should try reaching the villages, because here in town you are always here

**M:** now if one would like to reach you with information regarding health and testing, when not health education sessions, what other channels can be used to ensure that this message reaches gentlemen, because you have mentioned that some are not aware, others do what…..[left hanging]

**R:** phones, televisions…

**M:** because we want to know what gentlemen mostly use…

**R:** but there is no perfect means, because if I say that I give you my phone number it will mine personally, but there is no one who doesn’t watch television, and doesn’t listen to a radio at home, if an advert passes over a TV, let say there was something first then the advert comes next, that today is for meeting gentlemen in Wandegeya in this particular place

**R:** even others consult from their fellows that what happened in the news

**R:** that health workers are coming to Wandegeya to teach and it is for gentlemen, so you can plan that tomorrow I will go for the sensitization

**R:** and those adverts are always put at some points next to programs that attracts almost everyone, so they can advertise the scheduled place for the sensitization

**M:** now if someone would set a mechanism where you can do self-testing, do you think that can benefit the gentlemen, so that they don’t have to go to the health facility

**R:** it can work…

**R:** it can work….

**R:** it can work, though how is it established; not these that I test someone then after test blood and just want to please me, but tell me the truth, whether it requires me to start ARVs, then I start early but not hiding my problems,

**M:** I was talking about a mechanism say like the present pregnancy testing stripes, that a woman can go to any shop, buy the stripe and do self-testing, so if there is that method, do you thin k it can help the gentlemen,

**R:** it may help them…

**R:** it may help them because, he can die with his secret alone,

**R:** you see the gentlemen, here in dating ladies it may be easy, but going to the health workers for testing, you may even first swallow some tabulates before, you fear of the health worker knowing about yet you may not have a chance to meet again

**R:** it is so helpful…..

**R:** that method will be good

**R:** it can be good, if we are sensitized

**R:** it can help [then laughs] though it can inform you that you are [positive and even break it, but after all you have known….. [some laughs] and still you will go for treatment because you have discovered that you are positive

**R:** the challenge I foresee in that method, even many people are going for testing, and they may not know how to read the results, so you will find that one has bought it but will fail to use it accordingly, but for me I think there should be counselling then somebody is just tested

**M:** now the situation is that gentlemen most of the time, there is no way of reaching them that is why I have asked you that question, because some time back I think a few months, there was a method that was published in news and it was about what I have just told you, but the stripe uses saliva, you rub around the jaw both upper and lower jaws then you place it in a bottles and it reads like the one I told you about of the pregnancy test, so you just read say if it indicate two lines then it implies that you are positive, if it is just one line then it means that you are negative, how have ever heard about that method

**R:** some we just here rumors….

**R:** me too I have ever heard about it

**R:** I heard it from the lady, as you said that women are going for testing….., but still if it is brought, then you should set some sensitization for us too, on how it is used

**R:** but its mechanism if it is established, it is not bad I think……

**R:** I want to remove this man’s thinking that by that time someone gets the courage to go and test, it means you are determined to use it, if you go and buy that stripe that you want to see if you will test, still you will test yourself, especially if you have got sensitization on its operation, nobody will buy it when he is not going to use it because you have paid you money

**M:** you had some suggestion here….

**R:** and still we request the government to help us as it decided that everything used on HIV is for free so they should provide those stripes for free, get it and you test yourself, the paying is so painful than life, so they can provide and people test themselves; as they provide tabulates and other drugs, then they should provide them for free and see if it will be possible

**R:** and maybe I want to take you back on the issue of gathering places, some time back they had started a method when we could gather at mosques on Fridays, they could send some people after prayers then they could test people, if they can do that even in churches then it can help somehow, the gentlemen may go for testing from there

**M:** should we expect that people are going there….

**R:** they come

**R:** because many people pray

**R:** if you reach the church there are many people, let us if they are 1000 people and you get like 500, it means a lot

**R:** another issue those people that they send to us, someone may go for testing and they draw a lot of blood, and you wonder what the agenda was, was it for collecting blood, yet they have come to test and they take our blood, so I don’t know how they measure it in the bottles, but to me it is too much because they drew it from me and I developed

**M:** [laughs], yes sir you had a suggestion

**R:** to me I cannot allow too much blood to be drawn from me, because they have ever drawn it from me that is it madam

**R:** me what I emphasize, is to ensure that government brings that stripe, people to get that stripe and they can test themselves, they will be able to test and know their status, because he will be alone, so he will know and realise that it is time for getting treatment in order to be alive

**M:** so meaning if there is that method it will attract gentlemen to go for testing,

**R:** it will be more utilized as someone can easily use it and maybe inform his close friend and tell him about his status, there are those friends when he can easily inform that am positive and it helps to ensure that a person can get treatment

**R:** also the wife at home, if it has come, she can tell you that my dear let us know about our status, the same way she can tell you to purchase for her the pregnancy stripe, you go get it from the shop and she tests herself, meaning if it is there she can propose that we should test our statuses, if they can be distributed in all areas,

**M:** and it was going to be my next question that like what areas would you like them to be…

**R:** now there is this mechanism me I move in that town, but there is one health worker he goes on discouraging modern medicines, I don’t know whether you have ever seen him but he says that now you see, as you pour into the medicine into glass with water that that it is acid because they water makes foam others are in town and they talk about plants, they move while pouring in water so if they can also move around with this stripe [**M:** while showing you…], yeah while showing us how it works, people can gather and even make a circle while the other is seriously teaching them that they have drugs, and this is poison to you, this works like this and he ever pours it in water and you can observe, so even this one should be done in the same way within the public, like here they can say we just wait to see any gathering, and they join that they are trying out to show how the HIV testing stripe works, that was my suggestion…

**R:** to me what I request the government or other agencies, before we go to that stripe, the first thing should be counselling, to the youth or adult men and women, few people know when their friends are infected and they have the ability to either run away or not, meaning there is still a challenge some people don’t know that HIV can infect anyone, so if that counselling is done and people know that getting infected is not a case, so they will be able to go and get treatment, but sometime back counselling was more than what is done today…..

**M:** actually that issue is very important and I would like to ask, as we are here we are generating ideas on many issues, so regarding counselling to gentlemen, how would you like this to be done, let us consider two sides, today what is available that you go to the clinics, but now if someone sets such a method that you are going to do self-testing, how would you like counselling to be carried out on such an issue, how should you be helped….

**R:** me what I would like since it is a government setting, these days whatever they do is in clubs, and on the ladies side they have been helped so much through those clubs than us gentlemen, for us you fight on your own, even in the bank, if I have a family I cannot borrow money if the wife has not signed, implying even the banks are just for women, but we also need to be mobilised, the gentlemen, the youths, even in sports, we should get that information from there, that HIV is real though it can be cured

**M:** that information, how would you like to get it, using what because it will also help us in the issue of counselling if it is to done, how do you want it to be……

**R:** most of the time health workers organize say at Kabaka’s birthday run, aimed at helping people, such organizations can mobilize people together and it can be used in that way and people get to know

**R:** this issue am going to say it in a joking way, that in addition to counselling, there should be some attracting actions like comedy, how the comedian comes in, we are of different ages, and to me I can be knowing that HIV is dangerous and kills, but we have the young generation who are not yet aware of HIV, so even on TV, and especially these TV, there should be someone indicating how he acquired it, but he has managed to stay alive; hoping you are getting me, that he has also managed to stay alive while taking treatment promptly, so such advert should also be included very well, maybe it may help….

**M:** and others what do you think…, this gentleman had brought in the issue of counselling, and I was asking as you gentlemen, how would you like it to be conducted, we have got one suggestion, what do others have to say….

**R:** to me counselling, still you the very counsellors to teach on TV, you should not just say that you have put it on TV, you should remain passing on the information to people, because there are many who have no TV sets [**R:** others don’t get time to watch them….] and others don’t get time to watch and the children we sent to schools, they don’t have televisions, so it is better to find them in schools like in secondary, say like a play he was talking about, you can take it to different schools and children can witness such conditions and they realize that this thing is good or bad, so they can learn something, even us the matures ones we can learn, when my children are back home, we can teach ourselves in that way, and the good thing also I can use that stripe and test my child and know their status, the same way you can test young girls for pregnancy before going to school

**M:** this one had another suggestion….

**R:** my suggestion was, still I was requesting that those school going children should be catered for so much, because there are some children you can be with and you cannot explain to him as a parent but when others can elaborate it to him

**M:** still on that point I get a question, that you as gentlemen, what other category of people that would benefit in that method of self-testing if it was established

**R:** that method will benefit many people if they are to self-test, the children women, the grown up children say those at university, they may fear to go to the health facilities to be tested, but they can decide to do self-testing even without the consent of the parents, so that child after testing, he will decide to go for medication in the health facility, because he has kept his secret minus anyone, though he has started treatment even though the stripe provided wrong results, but he should start on treatment [**M:** he should start from there……], he starts the treatment….., I see that method helping if you have sensitized the community on how it operates, as there other one suggested that it should go beyond the television, others may not be watching, but these stripes need to be taught about within the public in different groups, say today we are in Wandegeya, tomorrow we are in Bwaise, even that one who might have missed Wandegeya session, tomorrow he will be in Makerere and he will learn how it works, not only but also distributing them

**R:** also regarding that stripe, the advantage that I anticipate, if you test yourself within your house alone and you find that you are positive, when you go to the health facility seeking for drugs, they are not just to give them to you, they have to repeat the test and confirm to you, that will help people to be tested in health facilities, even those who were not going there, so that stripe will be of great advantage to government to fight against HIV disease in Uganda

**R:** me am still sticking on the issues of news channels like TV, not everyone here in Uganda has got a TV, I know, but I want you to move around and ask whether there is anyone not aware of “baby ndunya” and “big pig” adverts [**M:** laughs] if there is anyone not aware, then they don’t watch televisions, even though they don’t have them at home, so even that one will work on the news channels [all laughs]

**M:** others what are you suggestions, I have heard that one……

**R:** I think the suggestions are done,

**M:** they are done, now, this gentleman some suggestion, but one issue that I also feel that I have not understood well, except those different channels that you have put across, how best can one deliver the information and ensure that every gentleman who got this message understands it, because some may not have understood it on those channels as stated, but in which other method can the information reach them and they understands it especially for like that new method, so that everyone understands how it works,

**R:** now it would be like……., because the government the government has got everyone’s number so it should be like a wedding planning meetings, where they send messages, say at Wandegeya market on this particular day there is a meeting, so even that advert should pass on everyone’s phone including the women, like on 15^th^ there will be counselling or testing at Wandegeya, when it is passed on our phone contacts, almost everyone has got a phone, so they will understand it

**R:** still the government has the capacity like it does for the phones for electricity, you can call and be explained to everything, so they can call us too, because they even call us on fake issues, yet this issue is very important

**M:** now on the issue of places, if those stripes are brought, where would you like to find those stripes, that if I want it, I can find it here, because remember it is personal, you have not given your suggestions on that

**R:** on that point I will take Wandegeya for example, we would request that there be a clinic in Wandegeya, because those stripes are not to be kept anywhere, that say in this particular clinic, it is for free, because the government has the ability to pay the clinic owner but whoever wants the stripe should go there and get it, but after identifying a particular clinic, they are ideal to be in clinics

**M:** this one has something to say…..

**R:** also even though they have not identified a private clinic, the government can get any other place, when they either own it or rent, but when people know that they can find them there, say at Wandegeya market at this particular room I can get it or at any other public place that maybe known to everyone in the area,

**M:** others what do you suggest…., should we expect that those two areas can be accessed by all gentlemen,

**R:** me what I suggest personally, cant that stripe be in one’s business say a shop and it can be there, but in a big facility like Mulago, they can encourage people to be picking those stripes from there when it is for free, because these days spending is the major challenge [**M:** I wanted to ask you about it, that if it is for sale….] because we shall reach time when putting it somewhere it will require more expense and they may need some money before getting them and you may reach a time when you don’t have the money, but Mulago hospital is available, that stripe is to help all Ugandans not only people in Wandegeya, whoever would like to test can access it, I think that can also help, if we are accessing it from Mulago…

**M:** what about those who may not access Mulago…..

**R:** there are those government general hospitals, like in Masaka, there is a hospital called Mulago,

**M:** this gentleman has some suggestion,

**R:** my suggestion is, you cannot say tha Mulago, Mulago Masaka, Mbarara, there are many say one in Ibanda when they even don’t know about Mulago, it needs when every sub-county, there is somewhere they can access it, so like am from Sembabule, now I cannot drive to Masaka, because it costs 7000 to go and 7000 return journey, that is 14000, will you still be in that deal…. , yet you will still come back home and fall sick, implying in every sub-county, it can help

**R:** and the other things also as we disperse the suggestion of our fellow, I suggest that Mulago should be left, because now days Mulago the drugs have been for free, but now you have to pay, but we need these places where there are many people say here in wandegeya, even though there are three places which are known so that even though someone at his free time can rash their and get the stripe to know his status…., that can be more beneficial that considering Mulago

**R:** while leaving Mulago issue, HIV is like the way you may eat food that you may eat now, like I came satisfied but now I feel hungry as I have been talking here [**M:** laughs] so I will go to Mulago and get it test when am negative but after three days I get another women who can infect me, still should I go to Mulago, why can’t it be in Wandegeya, it should have like three or four places

**R:** another thing that I suggest, as they send us those people to test us on streets and other public places, why can’t they set particular days, they move around while informing people that we have come to wandegeya today with the testing stripes, so residents of wandegeya, coma and pick but also the rest of the community too, I see that being simple that getting it from the hospitals, because in the health facilities, you hear of different issues, but when you reach there, there are more protocol to follow, some of us, it falls us to go to the health facilities….

**M:** now we have talked about this stripe, what you think if this method is started, do you anticipate any challenge that may be associated with, we have not discussed it yet, what do you think could be the problem with self-testing and what should be done to prevent such challenges

**R:** the challenge may one and it is on counselling, we need that stripe but there should be counselling first to the public, reason being some people are not strong, after knowing that he is positive, then he can do something wrong….., which is not good, yet if he got the counselling before, it can strengthen him like my fellow is still alive and am not the first, that can strengthen him so the major thing first should be counselling then others follow…..

**M:** so that point of counseling, would you like it to be done in groups like here or on individuals

**R:** the counselling should be like this [**R:** in group] that if you have decided to come to wandegeya today call them to come, because you will not come for counselling, the counsellor will sit there and counsels everyone individually, in a group like when we are seated here, some are not open to the health workers, so I may want to tell you something and I will fear because we are in a group yet if it was one on one I can tell you and you even explain to me very well and I will leave contended and I can do the testing….

**M:** now if one gives you this stripe and there is a number which you can call….

**R:** that thing is good, but the major issues of all, there are those who are going to get that stripe to and test themselves and they will still be doubting, people’s minds, but if there in counselling…. [**M:** he knows where to run to….], and they tell if you have doubt in the stripe after getting, run to this particular clinic and inquire from a counsellor you will be strong and even the people you find, they will strengthen you

**M:** this one had a suggestion…

**R:** what I think about that, I stick to the government because it has more capacity, it can afford everything, counselling if it is to take place, it should be a joke, it should be included like food reason being; there are many born again here but they go there simply because of food [**M:** laughs] an African if he is to be seated to be educated for free, even though it is sensitization they will not come, but if there is lunch there you will get many [all laugh] you will speak you word with his heart on food, but still they will listen [**M:** laughs] when they know there is eating some food [all laughs] they will not be seen…

Re, me what I think personally, that counselling would be good, but I think people should first get the stripes first before going for counselling, after using it, there he just go to get confirmation [some laughs] to establish whether they were correct results, because like they said, they always come here in the compound and test like 100 people and it indicates they are all negative, but after using this then I go to the health workers who come in the compound and I get what they tell me also, it would also be good, after using it…

**M:** let us listen to what your neighbours is suggesting

**R:** I oppose your suggestion, because you cannot first get the stripe before getting the sensitization, how are you going to use it then, counselling has two or three components; it is going to teach you how to use the stripe, it will strengthen you when you have confirmed that you are positive and the third is how you will use the drugs, you cannot use the stripe when you have not been taught and those to counsel you are the ones to teach about that stripe…

**R:** now what I also say, they always test us here and you see we are 300 and you have not got any positive one, [**R:** we have understood that……] what am saying why can’t I first test myself before going to them and confirm whether their tests are correct…

**M:** let us listen to this gentleman’s suggestion, I would be so much different to this respondent reason being to benefit from anything, you have to first understand it before, if you have not understood it, it means you cannot do it, so that stripe should come after getting sensitization about it then we go ahead to do what we have understood from what they have taught us, counselling a person is very important but all those, the stripe to come, there should be counselling before providing us with these stripes, am not so much different from this discussant, that is the most important…

**M:** now I want us to go back a bit to the issue we were discussing, what problems do you oversee this method, we only got one suggestion that brought all those ideas that we have got, others what do you think, is there any challenge that you anticipate in this method if it is brought and what should be done to ensure we prevent those challenges

**M:** to me the challenge I see is one, that were we have left before someone knows why he has got that stripe, and give it to him if he discovers that he is positive he can do harm to himself and second many people are nursed by their parents after the youths impregnated them after knowing that they are positive they run away, so that is why there is need for more sensitization so that they can understand, but maybe because of time madam as you know we are……[then laughs]

**R:** maybe we can get your suggestions

**M:** my suggestions you are going to get [then laughs] maybe, what I have been asking you, those are my suggestions from my side always, and I was going to ask is there anything that I have not asked about and you feel it is very crucial on these issues we have discussed today, anyone with a remaining suggestion or question

**R:** my question is, now you people are more aware than us, now on the issue of circumcision, that the chances of contradicting with the virus is reduced, that is what I want you to elaborate to us

**M:** that is one, the next…

**R:** me what I what you have not asked about yet, there are signs of any preventive measures of the HIV when one cannot infect the other

**M:** the virus…..

**R:** yeah….

**M:** ok, thanks so much for the questions, now let me first……, do you have any question sir

**R:** no

**M:** ok…..
