# Supplementary material for: Prevalence, associated factors and perspectives of HIV testing among men in Uganda
Source: PLoS One. 2020 Aug 7;15(8):e0237402. doi: 10.1371/journal.pone.0237402 (PMC7413494; doi:10.1371/journal.pone.0237402)
Supplement: S1 File — (ZIP) [file pone.0237402.s002.zip › manuscript data/FGD men 1-Eng.doc]

**FGD MEN 1**

**Identifier guide:** Moderator (M), Participant (P), Participant number (P#), Participant interjection (Pi), Moderator interjection (Mi), Participant Cross Talk (PCT)

**M:** *(sound of passing automobiles in the background).* Aah hmmn, may be am going to introduce myself again. I am glad to meet you all gentlemen. My name is **“M”**. Before we start our discussion, I would like to introduce the purpose of our discussion today. We are gathered here, for a reason as I explained before, I am from Mulago at the college of Health Sciences. We are conducting a research on matters relating to testing for HIV infection especially among men so as to improve health planning for them. So we are going to discuss and share ideas on testing for HIV infection especially among men in this community. This is also going to be done in several other places so as to obtain people’s views and opinions so as to appropriately plan for them.

Before we start, I request each of us to introduce ourselves. Each can tell us their preferred name or one which they would like to us for this discussion today.

**P1:** Can we start?

**M:** Yes

**P1:** I am called **“P1”**.

**M:** Please speak up

**P:** I am **“P1”**, I am **“P2”**

**M:** Please come closer sir so we can hear the voices. That’s why I was asking you to come closer.

**P: “P3”**, **“P4”**, **“P5”**, **“P6”**, **“P7”**.

**M:** I am also called **“M”**. So like I explained, the issue of testing for HIV must be something you are all familiar with. That is what we are going to talk about and that’s where we are going to start.

Today among you who are gathered here, what to do you think about testing for HIV? We are going to discuss and I encourage everyone to give their opinion. My role is to …

**P3:** It is very good for it enables you to know how relationship life has been fairing as well as your personal health status and life and how to live. In case you realize you’re sick, you can easily start to seek care and treatment.

**PCT#P2:** Yes, you can start form there.

**M:** Anyone else? It would be best if each of us give our opinions so we can discuss. What do the others say about this issue?

**P5:** It is helpful for someone to know his HIV results; it is not a crime. Yes… you see. To know your HIV status, it is your life, be it you’re infected or not. Every time you know your results you can decide on whether to stay safe or ….

**M:** What do the others say? *(Participants giggle in the background and some shy away from sharing their views)* Yes…..

**P7:** Testing, Testing for HIV is not bad. Testing is good for one to know the status of your life because if you can know how your life is. In case you find out how you’re doing, let’s say you, find that you are infected, you can look around for some treatment to take so as to prolong your life for a while so that you can fend for your family in the remaining years that you still have. But testing for is not bad.

**M:** *(M sighs “ummn”).* How about the others? What do you have to say about testing for HIV infection?

**Pi#P3:** Most importantly…..

**P2:** Testing is not bad, testing is good. However the challenge we find is … *(sighs)* that one can be very shocked (a lot of pressure) in case they are found. *(Giggling and laughter in the background from other respondent;* ***P3****)* You are filled with fear and say ‘eeeh’ for instance when medical personnel come to your area …

**PCT#P3:** *(Continues laughing).* They (medical personnel) care and prepare you. *(Continues laughing)*

**P2 continues:** … and say you come for testing. They might ask you; in case we find that you are infected, what will you do? *(****M*** *sighs “ummmn”)*. You tend to be filled with fear in your heart and for some of us that what makes …. us avoid testing *(M mentions in agreement “to test” and sigh “ummn’)*. So it is very helpful if God helps you and you find that you are safe then with that you are able to even take better care of yourself to remain negative because you fear interjecting the other pressure.

For me I once went, the testing was being done from just there in the compound. *(****M*** *sighs “ummmn”)*. So I said let me go and take the test, but the nurse first told me that they check for diabetes, blood pressure and HIV infection. So, she began by measuring blood pressure. *(****M*** *gives compelling sigh “ummmn”).* But she tied my hand, my pressure was so high, I could feel my heart pumping … and the nurse wondered so she asked me “why I are you so scared?”. *(****M*** *laughs lightly and gives a compelling sigh “ummn”,* ***P2*** *also attempt to laugh but cuts it short to continue his story, other participants giggle in the background)* So when she asked why I was so scared, she told me to go and sit on the side *(****M*** *gives compelling sigh “ummmn”)* so I can first relax on the side so that she could come back to measure and check me a little later. *(****M*** *continues to give a compelling sigh “ummmn”).* For me I just proceeded to the area of testing for HIV. *(****M*** *gives a filler “umm”)* So that fear can be overwhelming. *(****M*** *gives a sigh in agreement “ummmmn”).* After testing, I got my results and I was able to eventually relax and normalize. So that’s the challenge we find.

**M:** How about the others, what do you say about testing for HIV?

**Pi#P3:** *(saying to his neighbor)* You give you opinion, don’t just look on. *(and laughs)*

**P1:** Aaaah, testing is not bad, actually with the prevailing situation, the truth is that HIV infection is very high and widely spread. It is almost everywhere. It would be very good if everyone went and tested maybe. But sometimes you can find someone thinking …but today, there are so many lies within testing. You could go to test today and test and you even get the results but someone can come and … *(pauses)* and discourage you, saying … *(hangs for a while) that* “aaah” where you went … *(leaves sentence hanging).* For example I was told that those medical outreaches in the compounds basing on that person … *(pauses a bit)*, he did not get…. *(hangs a bit)*, he went there expecting to find himself infected …*(pauses)* but he went there about three times and got negative results each time so he wondered *(sighs “eeeh”),* if the testing materials they were using at that place were really genuine. So even you when you just go like that, we wonder *(expresses the wonder “iiiheeeh”)*, we tend to doubt the results you receive. So however much you get results, you still remain with “ummmn” very doubtful, very doubtful indeed *(****M*** *mentions the word “doubtful” in agreement with* ***P1****)*. So we request that in that method of testing, *(****M*** *gives a compelling sigh “ummn”)* is that … *(pauses)* they provide a transparent and trusted method …. *(hangs for a while)* whereby *(pauses)* if you have tested, you are contented with whatever results you get. Otherwise, let them educate people about testing; that if you test these can be all the possible situations so that people are free from that kind of fear. Because if you to go and test when you have “moved around among say three people; here there, there… and you receive contrary results …. *(leaves sentence hanging).* In someone’s life, some people is are serial “movers” I hope when I say “mover” you can understand *(****M*** *sighs in agreement ‘ummmn”)* serious “movers” but when they are even also worried about their lives. But if they go to test and … *(pauses)* always get “that” kind of results, they doubt and say ‘umn’, because they know they “moved” somewhere where they themselves suspected was dangerous and when it is the “fact”. *(****M*** *sighs in agreement ‘ummmn”)*. So in that instance, I request that when researching … (pauses) let them *(hangs a bit)* design a system whereby someone is contented with the outcome of the testing. *(****M*** *sighs in agreement ‘ummmn”)*.

**M:** That’s true indeed. We shall have a session closely ahead to ask some questions in case someone has some questions to ask and I will be giving them some answers and explanations. So, now, in this area other than you who are gathered here today, what to the people say about testing for HIV infection especially your fellow men? Because this arrangement is primarily targeting to improve services for the men. What do the fellow men there say about testing for HIV infection? Youth?

**P7:** Most of them support it but some don’t support it. Some especially fail to support it because they fear to find out that they are infected with HIV, most prefer not to know the truth. This is because they think if they discover the truth they will just begin to “feast” mercilessly and greedily on “everything” because after all they are already infected and might even start behaving in more undeserving ways. *(****M*** *laughs lightly and sighs ‘ummn”)*. But since that has kind of reduced these days compared to how it was at the start of the epidemic when we used to test *(****M*** *sighs ‘ummn”)* from Boman, when Boman still existed there. Nowadays people, most people are more aware of it, most of them would like to test to know their HIV status. Because even when an outreach is staged to call people to test, many people come out to test except that we have concerns with the kind of testing available and done. *(****M*** *sighs ‘ummn”)* Long ago for us we were tested with blood collected from here *“points to the hand vein — referring to venous blood”. (****M*** *verbalizes the pointing made by P7; “hand vein”).*

**P7 continues:** *(responds to* ***M****; yes)* They would tell us to go back home return after one week to pick the results *(****M*** *sighs ‘ummn”)* but today when we go to these tents here… they just use a small thing to pick the blood and in just a few minutes; five minutes they tell you you’re results after picking the blood from here *(points to the finger)* *(****M*** *sighs ‘ummn”)* yet before they would pick the blood from here *“points to the hand vein again” (****M*** *sighs ‘ummn”)* so we are not contented with that. We are not contented that is why my friend here *(point to* ***P1****)* said *(****M*** *sighs ‘eeh”)*, that is why he said that “someone tested three times yet they were know they are HIV positive.” Like for us, for me I can know, here many of us have indulged with “many” as we are here. *(****M*** *sighs ‘ummn”).* So for me I can know that this one is sick, he has so far lost about three wives *(****M*** *sighs ‘ummn”)* but he will still go out there to the tent and will be tested only for him to return and tell you “aaaah….. For me I am safe, do you see? Here is the card”, *(****M*** *sighs ‘ummn”)* yet for us who have lived with him here long enough with know that …. he is infected. *(****M*** *affirms “you know that he is infected”).*

**Mi:** But how did you get to know? That is something I am wondering about.

**P7 continues:** You see…., we could have buried his wife *(other participants including P7 burst out and laugh, members all start to giggle about the point made)* when they died from AIDS….

**PCT:** Despite not fading, when he is also already sick.

**P7 continues:** or when we know someone … *(pauses a bit) (****M*** *sighs in agreement “eeeeh … anyway”)* “eh” that “so and so” is already positive, yet our friend just goes ahead to have an affair with her *(Giggles and laughter continues from all amidst the talk.*

Mi: Anyway, it is possible. *(Still amidst giggles and laughter).*

**P7 continues:** ‘iiihh’. So do you see … *(hangs a while)*

**Mi:** But you see, these matters of HIV infection are very funny, *(P7 sighs in agreement ‘ummn”)* some people…. *(leaves sentence hanging).* I also have some who I worked with, someone would “get involved” with another who is already infected but this person would remain negative. *(Several participants sigh in agreement “iiiihh*”). So, that I why you see, we should not just guess. As you have been discussing, testing is good. It is very good because that the only way you can prove.

**PCT#P3:** You never know…

**M continues:** There are some people who do not even get infected with HIV at all *(****P*** *sighs in agreement “iieeh”)* but those ones, … *(hangs a bit)* we do not know how many are. Among every ten people you can’t know if you will even find any.

What do the other men out there think about testing for HIV infection? Does anyone else have a different opinion?

**P1:** For me, for me I will talk more about the youth because they are the ones I am more familiar with and among whom I spend most of my time. *(****M*** *gives compelling sigh ’ummn’).* Many youth …

**Mi:** Youth of which ages?

**P1:** From about 17, 18 up to about 25 years, *(****M*** *gives compelling sigh ’ummn’)* they are the ones among whom I spend most time. But one can come and tell you that he got a partner. *(****M*** *gives compelling sigh ’ummn’).* That even if he has got a partner those “tickets” they give after testing… what do you call them certificates *(****M*** *gives compelling sigh ’ummn’),* given after testing, that he can also just go across the street and have one made for him yet it purely a lie. So when he thinks about all that, some decide to go and test but in case you bring me that ‘thing’ when I have got my partner and they also bring me that ‘ticket’ but won’t accept that we go and test together it means I will also doubt that ‘ticket’. Do you understand? *(****M*** *gives compelling sigh ’ummn’).* So, some have that kind of thinking that even if you bring me a ‘ticket’ when we have not tested together they just abandon the entire program. I also hear some stories though I don’t know how wrong or right they are; that even if you have found a partner and you choose to go and test together they can swallow certain drugs ‘tablets’ even if it is instant or by ambush say in five minutes and the virus will hide such that even if they test, they will still return with negative results. *(****M*** *gives compelling sigh ’ummn’).* So, the youth also have that kind of mindset that even if you go together to test, someone can be already infected and still have negative results because no one will be ever come out in the open and boldly say that they are infected even if it is a fact that they are infected. So do you see, *(****M*** *gives compelling sigh ’ummn’)* but since everyone desires to spend some quality time with their partner ‘those drugs’ someone swallow can confuse since they hide the virus and then go ahead to enjoy but in the long run you just end up infecting each other. So, that issue also makes many youth reluctant to engage in testing. *(****M*** *sighs in agreement ’ummmmn’).*

**M:** So, for those who often test, where do they go? Those men who decide to test, where do they go? I have heard this gentleman *(points to* ***P1****)* saying that ….. *(leaves sentence hangings).*

**P1:** Most times, they tend to put outreaches in different places like they can come and stage here in the compound, or sometimes you can walk out there at Kampala road, in those kind of yards where they offer free HIV testing services. For me most people I know are very active in attending such outreaches but there are also other kind of people who for example if worried about their health can just walk to a pharmacy and pay maybe UGX 10,000= or 15,000= and get the test done. Yes madam.

**P2:** But that point you are talking about mostly works if you have ever tested before and even got to know your results and you get someone who you desire to ‘be with’ so much. But it is very hard if say that person stay on Entebbe road and for you, you stay in Nansana for example, and have come to ‘meet’ but we last ‘together’ about four months ago but now you want to ‘unite’ again in that case you all have to test again. Because for someone who ‘sleeps outside’ it is very had to be sure that they are secure and abstain to wait for you up to when you will return to them since you both sleep in different places. In about every four months, it would be good if you tested again but that is exceptional for this one with whom you share the bed every night here. Because you know that …..

**Mi:** So, for that one with whom you share the bed every night, what do you have to say? *(another participant laughs in the background).*

**P2 continues:** You see, the one with whom I share a bed every night, *(background laughter continues)* today, if for me I was tested and I want to sleep with her *(****M*** *gives compelling sigh ’ummn’)* then I have to take her so that we are tested again. *(****M*** *re-echoes ‘tested’).* So that we leave when we know what our statuses are. However beyond that point, only God knows and decides what happens but when for me I have confirmed for that moment. *(****M*** *echoes ‘for only that moment’).* Yes. *(Other participants continue to laugh).*

**M:** How about the others, what do you have to say? *(Laughter still continues).*

**P3:** For testing, like I said earlier there are some centres where you can go like in Kisenyi, the KCCA hospitals and other hospitals where there is free testing. But the only problem with that is the women, the women really disappoint us most. You can go to test and even get the results but these days women love money so much and there is no way to control or even hinder it. At one point you can say that ‘we have finished with the testing’, maybe one, two or three months or even twice a year but she will still cheat since you can’t have the same steamy love all through as it was in the beginning. In case you have a disagreement for example, it is very easy for women to ‘move out’ of the relationship because men are always asking her out.

**Mi:** Let me ask a question while you continue to give your views. For you who are here, how many times do you think someone should test? Let say in a year.

***P3:*** In a year whenever you have time

**All participants CT:** whenever you have time, whenever you find time

**P7:** Whenever you have time, you could be passing by a place where they are testing like city square and choose to branch and test too but testing every week can be hard.

**P2:** Whenever you have time, because for me I have told you I may have a girlfriend and we get tested today and find that we are all safe then proceed to have a good time then we split for say four months and then I call her again wondering where she is. So you will ask her if it is possible to meet and she will say yes. So if she comes, last time she had made her hair and it looked so nice but today if I see otherwise I will say ‘eeeh’, let us first go and test *(other participants burst into laughter)* since it is a while since we were together. It is only after testing and we are told that we are both safe. For me I just go back to doing the same thing as before even when you come back, we shall do the same thing until we part ways that is how it will be.

**PCT:** But concerning … (hangs)

**Mi:** Concerning? So you mean …. (pauses)

**P1:** I have something small to add to what he has just said especially among the youths and also among some married couples especially the newly married ones. These days things are a bit simplified; if your wife is going to deliver during antenatal care they often test and test. But it is true that youths when told that their wife or partner has been found negative they will wonder why they have to bother testing themselves too. Do you understand? That also exists among the youth.

**Pi#P5:** But when the women go for antenatal they are told, ‘bring you husband’. If you refuse they do not attend to you.

**P1 continues:** “iiiihh” they always treat and attend to them irrespective these days.

**Pi#P5:** They send them back. They send them back that ended.

**P1:** They attend to them. They actually attend to them. For me I have a friend with whom we recently went to the hospital. His wife delivered. After his wife delivered, they told them to both come for testing. The man just disappeared. But what stopped the lady form being tested is that the woman …. *(pauses)*

**PCT#P5:** That is different. You see when a woman is pregnant, when she is pregnant …

**PCT#P7:** It is just antenatal not delivery.

**PCT#P5:** Yes, it is antenatal. You have to come together both of you to be tested then she can start accessing all the other antenatal care services.

**Mi:** But now, according to you, how many men escort their wives for antenatal services?

**PCT#P5:** ‘Waaah’ are there any?

**PCT#P2:** Very few

**P5:** You just give her money and tell her to go to the hospital for antenatal. The men never go there. If she says that you are also needed he just says ‘aaaaahhh’, ‘Am I also sick?’, *(Other participants burst out and laugh),* yet the nurses want to find out if the child is truly safe. That’s what they want.

**M:** According to you, do you think it is right for the man to also test at that time?

**PCT#P5:** It is right. “eehhh’ it is right. It is very right if the woman is pregnant even if she is not, it is still right. It is right for both of you to test.

**P2:** It is right to test, both of you, because by the time the woman comes to call you, she would have been tested already.

**P7:** But still even if we go back to our first issue, you might find that one is infected yet the other is not. So at that time they could test and find that the wife is infected when for me I am fine or even when the wife is okay and for me I am infected

**PCT#P6:** Now, how do they do that?

**PCT#P4:** That is the end of that marriage.

**PCT#P5:** That is the end. So by the time you go you have your pregnancy and the only uniting factor is the unborn child. But still for a man it is still so hard because he will wonder ‘iiiih’, the woman is sick, so that child even though you know his/she is yours, they become … (hangs a bit), you raise them doubtfully. You believe that the mother cheated while you were with her that’s why she was infected while you were safe. Do you understand? So that child you are going to raise, honestly speaking in the name of God, some people can even deny that they are not their children just because of that scenario. For me I can believe because in case I go there and I am told that I am negative while the mother of the child is sick. I will wonder were the infection came from. That means she cheated. Do you see that?

**M:** But you see, this infection comes at different phases, and sometimes it takes a while before it can detected in blood that is why it would best if everyone knows their HIV status. So, in case it was your wife, would you escort her if she came for you to go and test together?

**P7: *(start listening at minute 18:43)***

**R:** to me the time I have spent on earth and the years I have spent I have tested for more than one hundred times, I don’t test because I have met them sometimes when donating blood, many times I just know if am negative, then the wife automatically she is…., and I have never took her and she has never been tested

**M:** and now your children, have you not been delivering them in the health facility?

**R:** this conditions came in when I am done, I had no young child [laughs] I no longer producing….

**M:** ok now let as proceed, now generally, what do you think are the major reasons preventing gentlemen from testing for HIV/AIDS, generally what prevent gentlemen from testing for HIV….

**R:** what prevents them majority from testing for HIV….

**Mi:** for HIV/AIDS….

**R:** most of us fear because of the reasons I tried to tell you, I fear being informed that I am positive, now I tell you that I developed high blood pressure, they even put me on drip [some laughs], the heart was seen by the health worker that it was pumping so fast, fear not because of what, but scared of being told that I am positive, so I shouldn’t go there, but still I was going while scared, I was shaking, and I decided to go bravely, I placed my hand, but from there the health worker recognised my challenge, and she was like, you as you are scared! So she advised me to first take a seat [all participants laughed], now many people fear you may say maybe where I dated, it was not safe so I will not go…

**R:** most people don’t go for testing, fearing, me I even lost my elder brother my elder had a wife, but the wife was aware that she was positive, but my brother was not aware that the wife was positive, now when she left, the wife told my brother that maybe, they had a child that they were nursing at home, that maybe as we were nursing the child, I think we were infected in the process, when my brother went for testing, he was positive, he tested like today on Monday, by Thursday we were burying him

**M:** oh sorry…

**R:** he even died before the wife

**M:** because of fear….

**R:** he feared so much and even stopped eating, he drank alcohol nonstop, aaaah and he ended up dying, yet the women lived for like three more years and she also died, so that is the issue in testing, most people have weak hearts….

**R:** weak….

R …because my brother was not good at cheating, he could tell us his siblings, he could say that we shall die and leave the burden of children for him because we had multiple partners [someone laughs], but what is surprising is that he died before us all, that is the challenge with testing, some have weak hearts but others become courageous

**R:** you have to be strong, if you are going for testing…..

**R:** the small addition to that, we the youth, if you have not yet produced, you have your plans in the mind, but now you have been informed that you are positive, eeeh, you have not yet produced, not yet constructed, even the parents are looking at you, how will you go to inform your father [laughs] that I acquired HIV, so when you look through all those situation, you say, please to hell with testing, let’s continue with life…

**M:** so you see it disturbing life….

**R:** that is very true, it disturbs life, because if you are told, you cannot remain the same and at least these days things have changed, it is no longer like those days, you can pretend, you live while on your medication, but still it will not change the condition, your life will change, that is why those with weak hearts, you find there life has changed, they remain there to consume alcohol, smoking marijuana, so they just live like anything, they no longer have an agenda in the world, so when you add up all that, then you put aside the issue of testing, God keeps you and you continue with life

**M:** and others who have not yet discussed, what prevents gentlemen from testing for HIV, there are those who have not yet discussed

**R:** to me I think pressure…

**M:** which pressure [participants laugh], apart from pressure, what else

**R:** because seriously those who are weak hearted cannot test, they can just inform you that you are positive and die immediately

**M:** so now, what can be done to improve in this system of testing to ensure we combat all these challenges that you have talked about that gentlemen face….

**R:** truly let me also say something now, [other participants laugh], what I would beg to be done that at least in every area, even in private clinic, testing should be for free, as long as you are in the preparation of fighting HIV, you cannot fight when one of you is not armed meaning we all need to fight, when in private clinics, it was set the government states it that whoever comes to test for HIV, because HIV someone to test is like committing suicide [some participants laugh], but if someone decided to test, some can say they cannot stand in the tents, they are there, you may find that Museveni cannot come into the tent, he may come to mobilize people but there are some who cannot stand there, someone will say my clinic is Habbi, but to go to Habbi to test, they will request for fifteen thousand shillings yet…

**M:** I don’t have it…

**R:** but if he knows that it is free in all health facilities, you just present your identification as a Ugandan or as any other person and then he….

**M:** tests

**R:** and we also request that, though there is some doubtfulness, they should give us correct results

**M:** ok we have heard that one also, any other person we another suggestion…

**R:** I would think that the government should implement compulsory testing….

**M:** among gentlemen, because we are looking at gentlemen, we want to improve these issues, but majorly among gentlemen, all people find challenges in testing but as you see ladies have their own challenges and the gentlemen also face their own challenges, and all the arrangements, the way you will organize for the ladies, it is not the same way you will organize for the gentlemen, it is the aim of these improvements that we are seeking, that is why I am asking that what should be done, because now we want to improve the process but while considering the gentlemen [noise from moving vehicle]

**R:** now my suggestion, testing should be forceful, because people have clubs, if you get to know that you are positive then you look for the club to join, so when you are there, you are like celebrities [noise from children playing], you know that I am like this so like Philly came up publically and announced, and he became the first Ugandan to declare

Pi, and that is how his fruits are reaped

**R:** you even wonder that time, he coming up there was no hope, but he came up publically, so if it is made compulsory, it has not started with you, because if you don’t get HIV, you will get diabetes, if you don’t acquire diabetes, you will acquire TB, so this should be compulsory and we get used to it

**M:** now on that issue, some days back there were news with a new method of testing for HIV that uses saliva, how many watched that news

**R:** me I watched….

**M:** you watched it, who has ever heard about that method, let us start from there, the method of testing for HIV using saliva

**R:** that [hangs up] I have ever heard it…

**R:** it is like they test for DNA, they test blood…, either the child is yours or not, if they test even the saliva even that may workout,

**M:** me too that is, I can explain to you, in that method, they bring a stripe, that stripe you rub at around the jaw outside, upper and lower gum, then you place in the bottle, the bottle is very small. Then you wait for like 15-20 minutes…

**R:** as the others do for pregnancy….

**M:** yeah, it was all over the news last week, like two weeks back, now like in 20 minutes you can know your status, but when you can do it personally

**R:** now those stripes are for sale or are free of charge

**M:** now all those are the methods that are coming up to ensure improvement and it is what I wanted to ask you about, what do you think about that method which you can test yourself personally, now it uses saliva, there is no pricking you…

**R:** it would be good….

**M:** because it tests for the HIV using saliva

**R:** you just spit on it….

**M:** no you get the stripe and rub it around the gum

**R:** now it is in the tongue….

**M:** the tongue is the source of the saliva at its bottom it generates it but the saliva circulates all around the mouth, it has no particular part that it is dry or it is

**R:** now it is how much…

**R:** the first thing….

**R:** is it for free of charge also….

**M:** they were showing it in the news but as I told you, it is still research, but still it is the government to decide that we shall put such a thing at this particular price, to enable people to access them when they are not so much expensive, how many heard about that method

**R:** I heard it, it was a program over the radio [noise from moving vehicles], but since it was research I never read it so much further and after I had watched a program over the television it was Dr….., who was on research on HIV, he was looking for some people who would commit themselves to be volunteers, they go and get some blood from them and see really whether the medicine that they had just discovered, whether it can be applicable in people, they were requesting people, they test them and then they are injected with that medicine, because they first tried it in rats, then to monkeys, and it was good, now then bring it to people now people should volunteer….

**R:** there is committing when you are positive already…

**R:** we don’t know what outcome will be…. [ooooh, One participant laughs]

**R:** they inject you with the HIV vaccine, and it is the one to fight against HIV [some participant laughs]

**M:** others on the method that I talked about, have you heard about it and what do you about it…

**R:** me, I have never heard about it and I know nothing about it….

**R:** the truth is even me I have never heard, but from your explanation, it wouldn’t be bad, but the government should take the responsibility to teach people how it operates, let me give you an example in these days there are some things, in fact even though you for testing that is what they will put on you, they have colors, I don’t know how they are called, where they place the blood, but we don’t know how to use them people don’t know them yet people should be buying and place them at home and test themselves at their convenience….

**M:** it is also going to work in the same way…..

**R:** but we don’t know them. you get it, so as the come the government should ensure that they teach the public, next you were talking of the measures we can avoid, the government should teach people that HIV is not a crime, why should I tell you that, I had my friend he was positive and he was my classmate he is still alive, but whenever it came to the time of taking drugs, he could just hide, he could say please accompany me, so such things on a positive person, the government should mind so much especially among students to teach them that the disease came and it is no longer going or it cannot be avoided, it came, but it is not a crime, people should not be discriminated, because many times you find even the mature people discriminating those people even before young children [phone rings], so it is a disease which came and we have to accept, nobody wishes it to come, but in that matter if the public realize that it is not a crime, that a person is free whenever you are, as you said that I can be there and present myself, I know my results and the status, that is what I request that the government should put more efforts in sensitization, to inform the whole country that this disease isn’t a crime

**M:** now it makes me to ask my last question I think as we come towards the end of the session; do you think if there is a way where gentlemen, because they want to improve for the gentlemen, even though there are other people, but now, if one can have self-testing for HIV, do you think that method can improve the way people may know there status, someone can know that this is my status

**R:** it is possible…

**M:** what do you think if there is a method where you can test yourself, you go and buy it from somewhere and you test yourself…

**R:** it have advantages and disadvantages…

**R:** automatically…

**R:** you will find after testing, he fears…

**R:** testing should be public

**R:** the person after buying, he will be scared and gets more and more scared without any counselling…

**M:** what if you get your partner won’t it be helpful, you decide and go for it

**R:** if he is going to get it in the pharmacy, they won’t tell him that please come along with your patner so we can sell to you this stripe, you get it alone, to get it alone and go for testing yet you have some fears and them the following day you…..

**R:** but for me I think, that after testing and knowing your status because most of the time a positive person, they want to spread the disease, most of the time

**R:** so he may not die alone…

**R:** so they want to spread, so if a person after testing and know you are positive and I wake up in the morning when I want a wife to marry, I will have to take her to the machine, so not to spend on this that I test myself then I come and lie to you that I have already tested and an negative yet am just aiming at infecting you, but if we go to the doctor and tells us that I will prick her so how much that fifteen thousand shillings, when I have it and I love my partner then I pay, and me telling you within not a long period I got my date an d I took her for blood testing, that is what I first told her before going to their home, I told her I am coming but we first go for blood testing

**M:** now as you, would you second the idea that someone can do self-testing, even though the hospital method is still available

**R:** it is very good…

**M:** would you like even this method where you personally can test yourself

**R:** I can test myself and know my status, it helps me to make my security so I will not have live sex, I will know like, eeeeeh the person I maybe going for maybe positive, so that help me to put on the condom to prevent, because I will be making security, if I know that I want to overtake and become mine, I suggest that we go for testing

**M:** uuuuum what other benefits do you see it,

**R:** me, from what he said that you cannot know that I have diabetes except when they have tested you, so if you go for the first time with your partner when there is no one who knows from that point and he will tell you the truth, because you test and know you status, even these stripes have advantages, they save time, it can help in hurry when you have just got someone, what should we do, you just go and buy your stripe [noise of vehicles] when everyone trusts each other because you will be knowing your status

**R:** for me there is one I know when HIV/AIDS was so much by that time, he was so rich, he knew that I have been infected, that gentleman starting enjoying life, you see, they become bad-hearted, he enjoyed life, but HIV has not killed him but now he is suffering from poverty because he spent all the money, he knew there was no life after so now these things which can enable us easily to know and you protect yourself

**R:** like you have got un abrupt date, you just get your stripe and use it and even give it to your partner other than just going live, most of the gentlemen just go live…

**R:** just go live…

**R:** to me I would second the method, it is good, but let it be to that one willing to use it, you get it!

**M:** now that brings me another question….

**R:** it should be self-decided why, that even though I have got my partner the truth in God I have gone for tests with many different people, but when going, before you tell her or before she tells you, you will be the first to go there, there is no way you will go for testing together when you have never been there, you get it!, you have to first go there alone and know, in this case you go when you are firm somehow, you know already your status….[some participants laugh]

**R:** that is what I said

**R:** you test, but if you say you are going direct, aaaaaaah, I come to you when I know

**M:** implying this stripe can be helpful…

**R:** yaaaah that is how it can be beneficial…

**M:** now if we talk about that issue that it can help you like this, according to you, like in which places these stripes can be placed to ensure mostly gentlemen that they can access them easily, because at first you were suggesting that gentlemen fear it prevents them from going for testing, in which places can we put these stripes to know that people will find them…

**R:** those stripes should be in every pharmacy because I may leave here and go, I have got a lady, either in Katanga you may have some condoms, yes, it is no longer strange, yet we used to hear it, if someone gets a condom in your pocket, it was strange, but because they have been distributed…

**M:** still it is our objective in this method also to ensure that…..

**R:** even this method, the condoms were strange, but now if you go to the health facilities in the rural setting…., the box is there if any feel interested you just pick and go protect yourself, so even these ones, we want them to be cheap…

**R:** or eve for free

**R:** that you can find it anywhere…

**R:** it shouldn’t be strange, you get it! You go and finish up your plan, you go meet her maybe in the evening, but I go with my two stripes, because one time I will not lie to you we came to testify facts, I got a lady [noise of moving vehicles] then I didn’t inform her that we are going for the testing, but because I knew my status, we reached at the entrance of the health facility and she was asking like where are taking me again, I was like we go and do some test, she was like eeeeh, you don’t first tell me, [some participants laugh] so I was questioning within me that do you first have to prepare [all laughs], does it require testing, but after I realised that she was an enemy to me and she wanted to infect me just, so if we can get such quick methods, it will help some people not to die, because if I did propose to go to the health facility and or she refuses and I come to know, is she not a murderer to me I see more advantages if they are accessible, they can safe…

**R:** they will safe people

**M:** and you sir what is your suggestion

**R:** don’t just bend your head [all participants laughed]

**R:** what I add on that, those stripes should be made when they are not confusing, in that if you put in, or if it indicates the saliva say red colour I just assume that red you are positive and blue you are negative, that one, but some things that they bring what, they over check,

**R:** but in this, I personally in my heart, I don’t think really that am sure that am negative but what am going to copulate with, I don’t trust, and I don’t really go to the doctor to test us, why should I buy this stripe that I cannot trust, let me go and they tell me the amount of money but when I get the joy of it all when I know….. [some participants laughs]

**R:** let me tell you one thing my brother what you should know still….

**R:** why should I put my life to test….

**R:** not all the time that whatever you trust, it can also be wrong, they can judge the book by its glittering cover but inside…

**R:** but the doctor has confirmed to me, he has told us, so why should I doubt again, while the doctor betray me,

**R:** for that I have understood, but….

**R:** too me, it is individually to know your status…

**R:** that is what I have told you…

**R:** what you need to know….

**R:** but why should I put my life to test

**M:** now the method of the health facility is still there, but as I have told you, we are aiming at improving, that is why we think of starting even this other method, both should be available and it is your decision

**R:** true…

**R:** me I will take it like this that as the girl may say that you write a letter and it spends months on the way without reaching its destination, now the world has changed, if that is the easy way, to simplify life, like a telephone call, if you want now, you can call in England, that is what they have brought for us, so it can easy in removing that satan

**M:** whom else do you think that they may benefit from this method of self-testing if it is started….

**R:** sorry repeat…

**M:** which other people can also benefit from this method especially among the gentlemen, because there are many categories, now I have met you here, which other people…

**R:** like the prostitutes

**M:** they can also benefit, what of among gentlemen, like among the gentlemen, because these jobs differentiate you, which category, doing which jobs that they may be improved for….

**R:** they have to improve for the long distance employees…

**R:** and the teachers [then laughs]

**R:** those on long distance employment, my wife is in the village [some participants laughs], I take like a month without going back, so whenever I go there I have to go prepared, because I will have to test her and myself, you get it!

**R:** the fishermen…

**R:** so those gentlemen who do long distance jobs, this method will help them so much, because you will not make it after the month that you are taking her to the health facility every time you are coming back, she will

**M:** be frustrated

**R:** she will be frustrated and think the husband doesn’t trust her, calls me a prostitute or what, so if she can do it herself, you can even put it on the table

**M:** what about in your community here, what other people do you think can benefit from this method….

**R:** there are many like business

**R:** now like the food vending women, and those food vendors get a lot of temptations from men who drive, she can meet a gentleman of a special hire, she doesn’t know, only that he is driving….

**R:** he is driving and he has sat her in a vehicle

R… yet she works for say three thousand or five thousand shillings, if she is given ten thousand or just a lift so if they are taught, they can be helped, in that style, it may help to reduce on the speed of disease spread

**M:** the way it is spread, what about others, and what do you think?

**R:** that gentleman talked about fishermen only that he was shying, at the landing sites, landing site at the lakes, HIV spread is very high, so that method I think it will benefit them the more at the landing sites…

**R:** yeah it is true

**M:** and others do you have any other suggestion on that issue, we are about stopping

**R:** on that stripe….

**M:** yeah, what about the challenges, one gentleman here talked of the challenges that may be as a result of this self-testing method, is there any person, what challenges do you foresee if this method is brought, one gentleman said you need to know how they will test, another was like they may commit suicide, what other challenges do think may result from this method of self-testing yourself alone if it is established…

**R:** the problem in that, the method which has been there, testing you first go through the counsellors, they give you strength, so you go when you are strong….

**M:** stronger…

**R:** but here you just go alone, you are like, you may even commit suicide [laughs] now that is the challenge that I see in it, that in this one you first get that counseling and become strong…

**R:** you have be strong…

**R:** they strengthen you and tell you what will be next, then you will say they have told me

**M:** and how do you think we can prevent such challenges that may be from self-testing especially among women, what should we prepare to prevent, to prevent challenges that may be like those ones

**R:** the challenge, you have to prepare as you said gentlemen and prepare the ladies also, because if you just prepare one side, another side will be wrong, because if the lady knows that she is positive, but she may refuse to go yet you feel you love her or sometimes you may even just meet once, because there are some who are in a relationship, but when your friend has been promising you for some time, you don’t have an opportunity to meet her but that day, God makes meets you people, you see how one infects the other…

**R:** you make the cross….

**R:** you just say the Lord who created me You the only one who knows my life [all participants laughed]

**M:** amen,

**R:** you see! You regret afterwards [all participants laugh], that what was killing me, when you are finished [all laughed] so you have to prepare all the sides, if the wife is found negative, find a gentleman and tell him please let us go for testing, if I am found negative, I tell the lady we go for …..

**M:** testing

**R:** those don’t work….

**M:** now….

**R:** my ideas is, what will help in preventing all; teaching all gentlemen in all the groups, so they know that if you are infected, life is still continuing, but still many even though all these methods came, some if they k now they are positive, he knows that is the end of life, so if these people are taught and they are given hope in the future, however much they are positive, they will be free to test all the time they like, but before you remove that obstacle [pauses for a while] testing I am positive, so the hope, the government should be more strategic to give hope to the HIV positive patients, that life is still there, that even the HIV positive can live up to 70 or 75 years, you get it! So all those things, the government should teach people

**M:** thank so much for your discussion…

**R:** now that a person to be tested, it will be easy, you get it! For us we do business there are things we do to ensure the client comes again, by giving him some bonus, so the government can say if a person comes when he is negative [noise of vehicles] we shall get you employment or if you are educated we shall employ you in a bigger position, if you are negative, the government will get you a million shillings, so even people will be protecting themselves, he is like maybe after this period of time the government will be testing, I will go with my paper and get such a thing, so the government should be thinking like that, I thought of it like a young child but it is the best, because if he is going for testing just to know his status, my dear it is not easy….

**R:** now in addition to what he said, that those people should be motivated, so even though the person is positive, but he is in a good life…

**R:** but if the government says that he is negative, he is on free medical care for diseases like malaria for the period that they can provide, so if you say the person is negative….

**M:** in that there should be a gift….

**R:** we shall say within me that….

**R:** it is a motivator… [laughs]

**R:** but if you look at the life of our fellows, we also need them to be alive, if they die, they we shall be ending it slowly, we should all live but even those who are remaining should be….

**M:** negative…

**R:** when they are negative, that is someone comes when he is negative, he will come and get free medication, and counsel him that since you have been found negative, please protect yourself, you have been offered free medication for a period of six months, but if you come back negative, you will pay for your bills of malaria, we shall be putting you on our treatment for HIV, but if you come when you are negative we shall put you on this treatment for diseases like malaria, influenza, headache, you will be coming here on government bill and get the medication, and it will be fast because we want you when you are negative and even the positive one, we have not chased you, we shall be giving other medication, we shall remove you from this treatment and take you to your appropriate treatment, the ARVs without any confusion, you will be providing him with his drugs, but for the case of malaria, there you go to another health facility for treatment

**M:** ok is there any other with another suggestion, I have asked so many questions, if you have any question for me also on this topic that we have been discussing, I also welcome it

**R:** my question, we hear that the medicine was discovered, others that it is not yet discovered, my question is that is the medicine there….

**M:** for the HIV virus?

**R:** yeah…

**M:** the medicine for HIV has not yet been approved, this gentleman brought one suggestion sometime back, there were some sessions that came and they wanted to try out, now this medicines moves in stages, by the time they bring it for use by people, it should have been applied on a number of stages, from animals, and from these small animals like rats, they move above to medium animals like monkeys, even in humans they start with a small number we can start with say three people, small number or children even the age, as you have seen this disease affects in different ways, but as per today, there is no medicine, what we have that can help our people is to prevent the disease and to test to know your status

**R:** now the other issue, aren’t there other preventive method that our people who are still negative as our country Uganda loves them there is no any vaccine that those who are still negative, that even though they have sex with the positive ones, they cannot be infected

**M:** that vaccine is available but still under trails, but it is there especially if you were raped, it was an accident, you were uprooting the tooth, it is there but what it contains is one thing, they should give it to you before elapsing of certain number of hours, you should not beyond let us say 72 hours, those are the three days, why, because they want to prevent the virus before it moves to circulation in the blood, now that medicine, it has worked for some, but as you see, it is still under research and some when they come, they lie and don’t say the truth, you ask say I have not refused, you had gone for a party and you over drank, so you didn’t understand what occurred next, but when they ask you, when did it take place, the aim being to help you count these hours so that we can know if you are given the medicine, it will benefit you, they want to help you but for you decide to lie the days, it was today morning, yet within you, you are aware that it was on Sunday evening, don’t you see that you will be the one lying, so that medicine is there to help them to ensure that they don’t acquire the virus, if they can get such instances, that maybe I was not well aware, or I had used something at that moment, it was not intentional, it was an accident, I was pricked by a needle that I was not sure of, they lie, but it is available if you speak the truth, they can give it to before elapsing of 72 hours after that instance has occurred

**R:** and it is very expensive….

**M:** but in that method, it is available in the government hospitals, they can offer it to you….

**R:** but it is very expensive

**M:** in government hospitals like those of KCCA that you talked about, if you and explain to the health workers very well, they can give you the medicine, but most of the time people lie….

**R:** lie…

**M:** yet as you see this medicine, it is the very one that prevents the virus, so if you don’t use it correctly, even you, you will be causing trouble to yourself, it is like if you decide to take the remaining dosage of coatem for a young child so if you are infected by the mosquitoes, coatem will no longer be effective to you, whenever you decide to take the remaining whenever the child gets sick that you are preventing, you will not be preventing, you will be causing more harm to yourself, so if you are bitten by mosquitoes yet the treatment is coatem by the time you will be using it, it will not be effective to you, so gentlemen, let me appreciate you all…

**R:** I have one more question, this HIV, how long does it take to enter into the body, how long does it take to be tested and confirm that you have it

**R:** how long does it take if I have sex with a positive person today, it take months, weeks, to go and test and they will tell me that it is positive, to circulate in the blood

**M:** depending on the methods that are available today, that is why they tell you if you have tested that come back after three months, meaning from the day…, anything may occur meaning that before the elapse of three months, it doesn’t mean the HIV is not there, but we don’t have a mechanism today that can reach people when it can confirm that you are positive before elapse of three months, but what it means that if you test today and you are negative, come back after three months and retest, but it doesn’t mean that you cannot test every month, because now you personally in your heart, you understand yourself better

**R:** that is true…

**M:** so you decide to go and test, so that is, ok members
