# Supplementary material for: Prevalence, associated factors and perspectives of HIV testing among men in Uganda
Source: PLoS One. 2020 Aug 7;15(8):e0237402. doi: 10.1371/journal.pone.0237402 (PMC7413494; doi:10.1371/journal.pone.0237402)
Supplement: S2 File — (ZIP) [file pone.0237402.s003.zip › Study tools/FGD topic guide.doc]

**FGD topic guide – SSI (Barriers and facilitators of HIV testing among men)**

**Topic:** Barriers and facilitators of HIV testing among men in Mpigi district

**Guiding instructions**

**Purpose:**

We are having this focus group to find out your views and thoughts about the barriers and facilitators of HIV testing among men in Mpigi district.

**Consent:**

Copies of informed consent and confidentiality forms should be provided to each participant and read aloud for the benefit of those who cannot read. Participants should be provided an opportunity to ask any questions. ***Verbal agreement should be taped.***

**Ground rules for the discussion:**

Before we start I would like to remind you that there are no right or wrong answers in this discussion. We are interested in knowing your thoughts and opinions, so please feel free to be frank and to share your point of view, regardless of whether you agree or disagree with what you hear. It is very important that we hear all your opinions. One of the grounds rules is that what I said here remains here so please do not make reference to who said what.

**To the moderator:**

Try to ask all the questions below in the order given, but it is more important to maintain the flow of discussion. Suggested probes have been included.

You should try to encourage participation of all group members in the conversation.

**Starting the dialogue**

**Member Introductions:**

Let's start by going around the circle and having each person introduce himself ***(Mention one name only). (Members of the research team should also introduce themselves and describe each of their roles.)***

Once again, the purpose of our discussion today is to find out your thoughts and opinions about oral HIVST among men in this community.

**Questions:**

1. There is a lot of promotion of HIV testing. What are your views about HIV testing and what do people in this community think about it?
2. More women than men go for HIV testing. Why do you think this is so?
3. In your opinion, what are the benefits of men testing for HIV?
4. In your opinion, what motivates men to test for HIV?
5. In your opinion, what challenges preventing men from testing for HIV?
6. In your opinion, what can be done to improve the increase the number of men testing for HIV testing?
7. We have discussed a lot of things; do you have any comments or questions about the topic?
