# Supplementary material for: Prevalence, associated factors and perspectives of HIV testing among men in Uganda
Source: PLoS One. 2020 Aug 7;15(8):e0237402. doi: 10.1371/journal.pone.0237402 (PMC7413494; doi:10.1371/journal.pone.0237402)
Supplement: S2 File — (ZIP) [file pone.0237402.s003.zip › Study tools/Survey Qnaire.doc]

**HIV testing among men in Mpigi district: Barriers and Facilitators**

**Baseline Survey Questionnaire**

**Guide Instructions:**

Check if the client consented to participate in the study (verify this from a signed consent form). Client’s informed. Consent status (Tick where applicable):	Yes □	No □

Administer a single questionnaire in a face-to-face interview to each eligible participant in any selected household at a time.

**Brief introduction:**

My name is *(mention it and fill in your initials)* ___________. As you know, a team of researchers from Makerere University are seeking information on HIV testing among men with an aim to develop ways to improve the delivery HIV testing services for the men in Mpigi and Uganda at large. Your contribution will help us assess the potential social impacts and develop mitigation measures.

Section1.0: Participant socio-demographics

| **##** | **QUESTIONS AND FILTERS** | **RESPONSE** | **VARIABLE NAME** |
| --- | --- | --- | --- |
| 1.1 | Date of interview (DD/MM/YYYY) | ☐☐ ☐☐ ☐☐☐☐ | INDAT |
| 1.2 | What is your name? |  | NAME |
| 1.3 | What is your age in complete years? | ☐☐☐ | AGE |
| 1.4 | What is your religion? | ☐1=Moslem ☐2=Anglican  ☐3=Catholic ☐4=Born-again  ☐5=Others | RELGN |
| 1.5 | In which Sub-county do you live? | ☐1=Nkozi ☐2=Mpigi T.C  ☐3=Kammengo ☐4=Kituntu  ☐5=Muduuma ☐6=Kirengente  ☐7=Buwama | CTY |
| 1.6 | Where do you live (Village)? |  | V ILL |
| 1.7 | What is your current marital status? | ☐1=single with a regular partner  ☐2=single with no partner  ☐3=currently married-monogamous  ☐4=currently married-polygamous  ☐5=cohabiting/living together  ☐6=widowed  ☐7=divorced/separated  ☐8=Never married | MARIS |
| 1.8 | Are you currently in school? | ☐1=Yes ☐0=No | SCHgoing |
| 1.9 | What is the highest level of education you have attained? | ☐1=No formal education  ☐2=Primary level (P1-P7)  ☐3=O-level (S1-S4)  ☐4=A-level (S5-S6)  ☐5=Tertiary & University | EDUC |
| 1.20 | Your do you do for a living *(occupation)*? *If response 1, jump to Qn. 1.23* | ☐1=I don’t work at all  ☐2=I do many things, nothing specific  ☐3=I am self-employed, *Specify* _______  ☐2=Employed, *Specify* ____________  ☐7=Others, Specify _______________ | OCCP |
| 1.21 | Household**^1^** ID  (daily incremental: DD-MM-####) | ☐☐ ☐☐ ☐☐☐☐ | HID |
| 1.22 | How many people**^2^** do you live with in the household? |  | HMNum |

**^1^**A **household** is defined as a group of people who routinely eat out of same pot and live on the same compound (or physical location).

**^2^** Include all routine household members in the table below. Include household members who are temporarily away, working outside the home, or at boarding school, but exclude visitors who have stayed for less than 3 months

**Section 2.0: HIV testing History**

| **##** | **QUESTIONS AND FILTERS** | **RESPONSE** | **VARIABLE NAME** |
| --- | --- | --- | --- |
| 2.1 | Do you know any place in this community where you can test for HIV?  *(If No, jump to Qn.3.5)* | ☐1=Yes ☐0=No | TESPLC |
| 2.2 | Which are the places where one can test for HIV in this community? *(Tick all possible options mentioned)(Probe the respondent)* | ☐1=Health centre/facility  ☐2=Private clinic  ☐3=Pharmacy/drug shop  ☐4=Community out-reach  ☐5=Others, specify ________________ | TESPLCS |
| 2.3 | Have you ever gone to test for HIV at any of the places you mentioned?  *(If No, jump to Qn.3.5)* | ☐1=Yes ☐0=No | TESGO |
| 2.4 | Which place did you go to? | ☐1=Health centre/facility  ☐2=Private clinic  ☐3=Pharmacy/drug shop  ☐4=Community out-reach  ☐5=Others, specify _________________ | TESGOPLC |
| 2.5 | In life, have you ever tested for HIV?  *(If No, jump to Qn.3.14)* | ☐1=Yes ☐0=No | TESEVER |
| 2.6 | Have you tested for HIV in the past 12 months? | ☐1=Yes ☐0=No | TESLASYR |
| 2.7 | When was the last time you tested for HIV? | ☐1= ≤ 3months ago  ☐2= 4-6 months ago  ☐3= 7-9 months ago  ☐4= 10-12 months ago  ☐5=>12 months ago  ☐6=I don’t remember | TESLAS |
| 2.8 | Where did you take the HIV test from? | ☐1=Health centre/facility  ☐2=Private clinic  ☐3=Pharmacy/drug shop  ☐4=Community out-reach  ☐5=Others, specify _________________ | TESPLC1 |
| 2.9 | Did you wait to receive your HIV test results? | ☐1=Yes ☐0=No | TESWAIT |
| 2.10 | Did you disclose your HIV results to anyone close to you?  *(If No/I don’t remember, jump to Qn.3.14)* | ☐0=No  ☐1=Yes  ☐2=I don’t remember | TESDISCLO |
| 2.11 | To whom did you disclose your test results? | ☐1= Partner ☐2= Workmate  ☐3= Friend ☐4= Mother/Father  ☐5= Sister/Brother ☐6= Other relative | DISCLOPER |
| 2.12 | What were the results from your last HIV test? | ☐0=Not willing to disclose  ☐1=Negative ☐2=Positive | TESRES |
| 2.13 | In a given year, how many times do you usually test for HIV? | ☐1= Once a year ☐2= 2-3 times a year  ☐3= >3 times a year  ☐4= Sometimes I don’t test at all | TESYEAR |
| 2.14 | Are you aware of your partner’s HIV status? *(If No, jump to section 4.0)* | ☐1=Yes ☐0=No | PATHIV |
| 2.15 | Have you ever taken an HIV test and received the test results together with your current partner? | ☐1=Yes ☐0=No | COUPTES |
| 2.16 | In the past 12 months, have you and your current partner taken an HIV test and received the test results together? | ☐1=Yes ☐0=No | COUPTES1 |
| 2.17 | What was your partner’s HIV status? | ☐1=Positive ☐0=Negative | PATHIV1 |

**Section 3.0: HIV risky behaviors**

| **##** | **QUESTIONS AND FILTERS** | **RESPONSE** | **VARIABLE NAME** |
| --- | --- | --- | --- |
| 3.1 | Are you circumcised? | ☐0= No ☐1= Yes | CIRCUM |
| 3.2 | How many sexual partners have you had in the last 6 months? (If you are uncertain, you can estimate.) | ☐☐☐ | PASTSEXPAT |
| 3.3 | How many sexual partners do you still have presently? | ☐☐☐ | CURSEXPAT |
| 3.4 | In the past 6 months, have you had sexual intercourse with someone who was not your wife (partner) / not your regular partner? | ☐0= No ☐1= Yes  ☐2= I don’t remember | NONPAT |
| 3.5 | Do you always use a condom when having sexual intercourse? | ☐0= No ☐1= Yes  ☐2= Sometimes | COSCOND |
| 3.6 | Did you use a condom in the last sexual intercourse you had? | ☐0= No ☐1= Yes  ☐2= I don’t remember | LASTCOND |
| 3.7 | Have you ever traded anything like money, housing, drugs, transport, or any other goods in exchange for sex in the past 12 months? | ☐0= No ☐1=Yes | TRANSEX |
| 3.8 | Do you take any alcoholic beverages? *(If No, jump to Qn.4.13)* | ☐0= No ☐1=Yes | ALC |
| 3.9 | Which type of alcoholic beverage do you take? | ☐1= Beer ☐2= Spirit/ whisky  ☐3= Vodka/gin/waragi  ☐4= Local brew  ☐5= Other, *specify* ___________ | ALCTYP |
| 3.10 | At what time of the day do you usually take the alcoholic beverage? | ☐1= Early in the morning  ☐2= During my lunch break  ☐3= In the evening after work  ☐4= All day as I work  ☐5= Other, *specify* ___________ | ALCTIME |
| 3.11 | How about in a week, how many times do you take an alcoholic drink? | ☐1= Once a week  ☐2= 2-4 times a week  ☐3= Every day of the week  ☐4= Once a month  ☐5= Other, *specify* ___________ | ALCWEEK |
| 3.12 | Do you use any intoxicant besides alcohol? | ☐0= No ☐1=Yes | INTX |
| 3.13 | Which intoxicant do you use? | ☐1= Marijuana ☐2= Kubba  ☐3= Petrol ☐4= Khat  ☐5= Injecting drugs, __________  ☐6= Other, *specify* ___________ | INTXTYP |
| 3.14 | Do you have thoughts of engaging in sexual intercourse after taking/using alcoholic or an intoxicant? | ☐0= No ☐1=Yes  ☐2=Sometimes | ALCSEX |
| 3.15 | After you have taken alcohol and/or intoxicant, do you always use a condom during sexual intercourse? | ☐0= No ☐1=Yes  ☐2=Sometimes | ALCINTXSEX |

**Section 4.0: Barriers and facilitators to HIV testing**

| ***Now, I want to ask a few question about the challenges you have found or what has hindered you from testing for HIV as well as what has motivated or what or could motivate you to test*** | | | |
| --- | --- | --- | --- |
| **##** | **QUESTIONS AND FILTERS** | **RESPONSE** | **VARIABLE NAME** |
| 4.1 | What motivated you or could motivate you to go and test for HIV? *(Tick all possible options mentioned)(Probe the respondent)* | ☐1= My friends encouraged me  ☐2= I wanted to be circumcised  ☐3= I had a new partner  ☐4= Free testing was/are brought near me  ☐5=There are many clinics, I can pay to test  ☐6= My lifestyle involves risky behavior  ☐7= I just suspected I could be sick  ☐8= Someone close to me was had test positive  ☐9= I was too sickly (Being symptomatic)  ☐10= I know there is treatment  ☐11= I am someone who is positive and has lived long but on treatment  ☐12= Other, *specify* _________________ | TESMOTIV |
| 4.2 | What hindered you or could hinder you from testing for HIV? *(Tick all possible options mentioned)(Probe the respondent)* | ☐1= I am strong enough to without knowing  ☐2= I fear to know the truth  ☐3= I know I am safe  ☐4= As long as my wife is safe, I am safe  ☐5= I can’t bear the thought of having HIV  ☐6= I prefer not to know my status  ☐7= I don’t trust the testing of these days  ☐8= With my life I am sure I am safe  ☐9= It is expensive to pay for a test  ☐10= I can’t be seen lining up for free tests  ☐11= In hospital you wait for too long to test  ☐12= Health workers are rude, they just shout  ☐13= The health centre is very far from here  ☐14= When people see you testing, rumors start  ☐15= With my work I don’t have time to go test  ☐16= The health centre and clinics close up early  ☐17= Hospitals are not comfortable for men  ☐18= I need privacy to test  ☐19= No one has encouraged to test  ☐20= Honestly, I don’t want to be pricked  ☐22= Other, *specify* __________________ | TESBARR |
| 4.3 | What do you think can be done to improve the provision of HIV testing services in this community *(Tick all possible options mentioned) (Probe the respondent)* (especially for the men)? | ☐1= Make free testing more available  ☐2= Testing prices should be reduced  ☐3= Bring testing services closer to people  ☐4= Increase opening time of clinics  ☐6= Bring new tests on board  ☐5= Increase awareness of HIV testing services available in the area  ☐14= Other, *specify* ___________ | TESIMPRV |
